# Supplementary material for: Assessing the Effects of Rotifer Feed Enrichments on Turbot (Scophthalmus maximus) Larvae and Post-Larvae Gut-Associated Bacterial Communities
Source: Microorganisms. 2023 Feb 17;11(2):520. doi: 10.3390/microorganisms11020520 (PMC9962078; doi:10.3390/microorganisms11020520)
Supplement: Supplementary file 1 [file microorganisms-11-00520-s001.zip › Supplementary File S1.pdf]

Assessing the Effects of Rotifer Feed Enrichments on Turbot (Scophthalmus maximus) Larvae and Post-Larvae Gut-Associated Bacterial Communities

Antonio Louvado[1], Carolina Castro[2], Davide A. M. Silva[1], Vanessa Oliveira[1], Luís E. C. Conceição[3],

Daniel F. R. Cleary[1] and Newton C. M. Gomes[1]*

[1]Department of Biology and Centre for Environmental and Marine Studies (CESAM), University of Aveiro, 3810-193 Aveiro, Portugal

[2]Flatlantic—Actividades Piscícolas, SA 3070-732 Praia de Mira, Portugal

[3]SPAROS Lda., 8700-221 Olhao Portugal

Email: gomesncm@ua.pt

List of representative sequences of deleted ASVs

>0058a381e13c111622df567cc7af962f

tgaggaatattggacaatgagggaaaccttgatcctacgatactgcgtgaatgaatgaagactctttgagttgtaaaattctttcattaaggaag
ataatgacgttacttaaaaaagaagttccggctaactccgtgccagcagccgcggtaagacggagggaactagtgttattcggaataactgggc
gtaaagggtatgtaggcggtttattaagttttaggtgaaagtttaaagcttaactttaaaattatttaaaatacttttaaacttgagtttaatagagg
ataatggaatttttagtgtagaaataaaatttgtaaatattaaaaggaactccagaagcgaaggcgattatctggattaacactgacgctgaggt
acgaaaggatggggagcaacca

>00963ec1f216fdf2e4d47372061ec17e

tggggaattttccgcaatgggcgaaagcctgacggagcaatgccgcgtggaggtagaaggcccacgggtcatgaacttcttttcccggagaaga
agcaatgacggtatctggggaataagcatcggctaactctgtgccagcagccgcggtaatacagaggatgcaagcgttatccggaatgattggg
cgtaaagcgtctgtaggtggctttttaagtccgccgtcaaatcccagggctcaactctggacaggcggtggaaactaccaagctggagtacggta
ggggcagagggaatttccggtggagcggtgaaatgcgtagagatcggaaagaacaccaacggcgaaagcactctgctgggccgacactgaca
ctgagagacgaaagctaggggagcgaatgggattagataccccctgt

>011a4a80f145da3138a59a20262629c4

aaacaagctgtaaacacaacattgacatatcgtcactttccgaagttataaaaaaacttcgtagcctaatcagctagtggctgattttagttcttat
gagaactttttggaacaacagccgtctgctgtgtccaacaatgatatcgatgagagccaggagagcgaacaaaatcaatacatttgctggcca
gaaaagcaaaatgaagggatgacgaaagatccacgtgatctattgtctatctgatctgccaatgaagatgt

>0198b2c0e198373d78e1237c3a42d248

taaggaatattgggcaatggaggcaactctgacccagccatgccgcgtgaaggatgacggccctatgggttgtaaacttcttttatacgggaata
aacccgggtacgtgtacccggctgaatgtaccgtaagaataaggatcggctaactccgtgccagcagccgcggtaatacggaggatccaagcg
ttatccggattcattgggtttaaagggtgcgtaggcggatttgtaagtcagcggtgaaatcctacagcttaactgtagaactgccgttgatactgc
aagtcttgagtacatttgatgtgggcggaatgtgtcatgtagcggtgaaatgcttagatatgacacggaacaccgattgcgaaggcagctcaca
aaactgtaactgacgctgaggcacgacagcgtggggatcaaaca

>01c17cc458e8abbcc102b808e45ffa54

tgaggaatattgcacaatgggggaaaccctgatgcagcaacgccgcgtggaggattgacggtctttggattgtaaactccttttctgtgggacga
gcgaggacggtaccacaggaatcagtcacggctaactacgtgccagcagccgcggtaatacgtaggtggcgagcgttatccggaattactggg
cgtaaagcgcaagcaggcggctcgttaagtttgacgtaaaatctcctggcttaactgggaggggtcgttggagactggcgagcttgaggcgatg

agaggggcatggaattcccggtgtagtggtggaatgcgtagagatcgggaggaacaccagaggcgaaagcggtgccctggcattggcctgac
gctcatgtgcgaaagcgtggggagcgaacg

>01f2b94783ffc09866d2d8bd4d14d8a3

tggggaatattggacaatgggcgcaagcctgatccagcaatgccgcgtgggtgaagacggtctgcggattgtaaagccctttaggcggggagg
aacgtcgtcgtgcgaacagtgcggcgatctgacgttacccgcagaataagcaccggctaactccgtgccagcagccgcggtaatacggagggt
gcgagcgttaatcggaattactgggcgtaaagcgtgcgtaggcggatgggtcagtcagccgtgaaagccccgggctcaacctgggaacggcgg
ttgagacggcccgtctcgagtgggctagaggatcgtggaattccgggtgtagcggtgaaatgcgtagagatccggaggaacaccgatggcgaa
ggcagcgatctggggccacactgacgctgaggcacgaaagcgtggggagcaaaca

>0238e0e03ffd3faa629954545d336e61

taaggaatattggtcaatggacgaaagtctgaaccagccatcccgcgtgcaggatgactgccctatgggttgtaaactgcttttagtagagaaga
aacgcagtcatttatgattgcctgacggtacctacagaataagcaccggctaactccgtgccagcagccgcggtaatacggagggtgcgagcgt
tatccggaatcactgggtttaaagggtgcgtaggcggggaattaagtcagtggtgaaatcttgtcgcttaacgataaaattgccattgatactgat
tttcttgaattgggttgaggttagcggaatgtgacatgtagcggtgaaatgcatagatatgtcatggaacaccaattgcgaaggcagctagctgg
gccttgattgacgctgaggcacgaaagcgtgggtagcgaaca

>02880bca12c185a77358ab268fc9d74a

tggggaattttggacaatgggcgcaagcctgatccagcaatgccgcgtgcgggaagaaggccttcgggttgtaaaccgcttttggcaggaacga
aacggtgtccgctaataccgggcgctaatgacggtacctgcagaataagcaccggctaactacgtgccagcagccgcggtaatacgtagggtg
cgagcgttaatcggaattactgggcgtaaagcgtgcgcaggcggttgtgtaagacaggcgtgaaatccccgggctcaacctgggaatggcgctt
gtgactgcacggctggagtgcggcagagggggatggaattccgcgtgtagcagtgaaatgcgtagatatgcggaggaacaccgatggcgaag
gcaatcccctgggcctgcactgacgctcatgcacgaaagcgtggggagcaaaca

>02f91d244dc4a89c804a57e3a4a2012b

tagggaatcttccgcaatggacgaaagtctgacggagcaacgccgcgtgtatgaagaaggccttcgggttgtaaagtactttcagtcgtgagga
aggcatttgcgttaatagcgtaagtgtttgacgttagcgacagaagaagcaccggctaactccgtgccagcagccgcggtaatacggagggtgc
gagcgttaatcggaattactgggcgtaaagcgcatgcaggtggttcgttaagtcagatgtgaaagcccggggctcaacctcggaattgcatttga
aactggcggactagagtgctgtagaggggggtagaatttcaggtgtagcggtgaaatgcgtagagatctgaaggaataccggtggcgaaggcg
gccccctggacagacactgacactcagatgcgaaagcgtggggagcaaaca

>031470c2d1c07877ef85cc2b5b3b3ba3

tgaggaattttccgcaatgggcgaaagcctgacggagcaataccgcgtggaggatgacggcctgtgggtcttaaactccttttcttagagaaga
agttctgacggtatctaaggaataagcatcggctaactccgtgccagcagccgcggtaatacgggggatgcaagcgttatccggaatgattggg
cgtaaagcgtctgtaggtggctttccaagtcaactgtcaaaaatcagggcttaaccttgagccggcagttgaaactatgaagcttgagttaggta
ggggcagagggaattcctagtgtagcggtgaaatgcgtagatattaggaagaacaccaatcgcgaaagcactctgctgggccatgactgacac
tgagagacgaaagctaggggagcgaatgggattagataccccggtagt

>032acccb1c0145513294dfd19d8ad7e9

ttaggaattttccacaatgggcgaaagcctgatggagcaatgccgcgtgcaggatgaaggccttagggttgtaaactgcttttatacgtgaagaa
tatgacggtaacgtatgaataaggatcggctaactccgtgccagcagccgcggtcatacggaggatccaagcgttatccggatttactgggcgta
aagagttgcgtaggtggtaatttaagtgaatagtgaaatcttgtggctcaaccataaggctattattcaaactgagatacttgagaatgagagag
gtcactggaattccctgtgtaggagtgaaatccgtagatatagggaggaacaccaatggcgtaggcaggtgactggctcatttctgacactgag
gcacgaaagcgtggggagcgaacg

>039aa10851da48633b422764ba7a8659

tggggaatattggacaatgggcgcaagcctgatccagccatgccgcgtgtgtgaagaaggccttttggttgtaaagcactttaagcgaggagga ggctacttggattaatactctaggatagtggacgttactcgcagaataagcaccggctaactctgtgccagcagccgcggtaatacagagggtgc gagcgttaatcggatttactgggcgtaaagcgtgcgtaggcggcttcttaagtcggatgtgaaatccctgagcttaacttaggaattgcattcgat actgggaagctagagtatgggagaggatggtagaattccaggtgtagcggtgaaatgcgtagagatctggaggaataccgatggcgaaggca gccatctggcctaatactgacgctgaggtacgaaagcatggggagcaaaca

>03b6ba062eff1aacc8b3fe788054afa0

tagggaatcttccgcaatgggcgaaagcctgacggagcaacgccgcgtgagtgatgaaggtcttcggatcgtaaaactctgttattagggaaga acaaatgtgtaagtaactatgcacgtcttgacggtacctaatcagaaagccacggctaactacgtgccagcagccgcggtaatacgtaggtggc aagcgttatccggaattattgggcgtaaagcgcgcgtaggcggtttttttaagtctgatgtgaaagcccacggctcaaccgtggagggtcattgga aactggaaaacttgagtgcagaagaggaaagtggaattccatgtgtagcggtgaaatgcgcagagatatggaggaacaccagtggcgaaggc gactttctggtctgtaactgacgctgatgtgcgaaagcgtggggatcaaacaggattagataccctggtag

>03c6444fe94266556629f3c77d76d543

ccaccttacctacggggggctgcagtgaggaattttccgcaatgggcgaaagcctgacggagcaataccgcgtggaggatgacggcctgtgggt cttaaactccttttcttagagaagaagttctgacggtatctaaggaataagcatcggctaactccgtgccagcagccgcggtaatacgggggatg caagcgttatccggaatgattgggcgtaaagcgtctgtaggtggctttccaagtcaactgtcaaaaatcagggcttaaccttgagccggcagttg aaactatgaagcttgagttaggtaggggcagagggaattcctagtgtagcggtgaaatgcgtagatattaggaagaacaccaatcg

>03d13d566b47a0b83fd1cd1294416e57

taaggaatattggtcaatggacgaaagtctgaaccagccatgccgcgtggaggatgaaggtcctctggattgtaaacttcttttatatgggacga aaaaagggttttctaactcgtctgacggtaccatatgaataagcaccggctaactccgtgccagcagccgcggtaatacggagggtgcaagcgt tatccggattcactgggtttaaagggtgcgtaggtgggttggtaagtcagtggtgaaatctccgagcttaacttggaaactgccattgatactatc agtcttgaatattgtggaggtcagcggaatatgtcatgtagcggtgaaatgcttagatatgacatagaacaccaattgcgaaggcagctggctac acatctattgacactgaggcacgaaagcgtggggatcaaaca

>03d8671072e521e192d538f851889518

tggggaatcttccgcaatgggcgaaagcctgacggagcaatctcgtgtgagggattagtctcgtgagggacggaaagtctacggattgtaaacc tctttttcagggaggaatacaatgacgtgtatctgaagaataagcatcggctaactccgtgccagcagccgcggtaagacggaggatgcaagt gttatccggaattattgggcgtaaagcgtttgtaggtggttaaataagtcaattgttaaatcttgaagcttaacttcaaaattgcaatcgaaactat tagactagagtatagtaagggtaacaggaattttcagtggagcggtgaaatgtgtagatattgaaaggaacatcagtggcgaaagcgttttact aggttaatactgacactcagaaactaaagctaggggagcaaatg

>03d8ff08c34d9401cd05db90944cc4f5

tcgagaatcattcgcaatgggcgaaagcctgacgatgcgacgccgtgtgagcgatgaaggccttagggtcgtaaagctctttcgcttgggaaca agagaagctagttaatacctagctaatttgagggtaccaggtaaagaagcaccggctaactccgtgccagcagctgcggtaatacggagggtg caagcattaatcggatttattgggcgtaaagggcgcgtaggctggtttgtaagtcagatgtgaaatcccggggctcaacctcggaacagcatatg aaactacatacctagagggtagaaggagaaaacggaattccacaagtagcggtgaaatgcgtagatatgtggaagaacaccggtggcgaagg cggttttctattttattcctgacgctgaggcgcgaaagcaaggggatcaaaca

>040c3e2578ae8716f5f66274b670f9a2

tggggaatattgcacaatgggggaaaccctgatgcagccatgccgcgtgtgtgaagaaggccttcgggttgtaaagcactttcagttgtgagga aaggttagtagttaataactgctagctgtgacgttagcaacagaagaaggaccggctaactccgtgccagcagccgcggtaatacgggggatg caagcgttatccggaatgattgggcgtaaagcgtctgtaggtggcttttcaagtccgccgtcaaatcccagggctcaaccctggacaggcggtgg aaactaccaagctggagtacggtaggggcagagggaatttccggtggagcggtgaaatgcattgagatcggaaagaacaccaacggcgaaa gcactctgctgggccgacactgacactgagagacgaaagctaggggagcaaatg

>049abd9f0f9a0d26b4205dbad6e7ed89

ttaggaatattcgtcaatggaggaaactctgaacgagcaatgccgcgtgagtgatgaaggtcttatagattgtaaaactctgttgtatggaaaaa
acgactaggttaggaaatgaacttagtgtgatggtaccataccagaaagccccggctaactacgtgccagcagccgcggtaatacgtaggggg
caagcgttatccggatttattgggcgtaaagcgtgtgtaggcggtttattaagtttaagattaaagcccggggctcaactccggtaagtcttaaaa
actggtagacttgagtacggtagaggcaaacggaatttctagtgtagcggtgaaatgcgtagatattagaaggaacaccagtggcgaaagcgg
tttgctgggccgttactgacgctgaggcacgaaagcgtggggagcaaata

>04b0f4595a4859b9ca2619890f26cb74

tggggaattttccgcaatgggcgaaagcctgacggagcaatgccgcgtggaggtagaaggcccacgggtcatgaacttcttttcccggagaaga
agcaatgacggtatctggggaataagcatcggctaactctgtgccagcagccgcggtaatacagaggatgcaagcgttatccggaatgattggg
cgtaaagcgtctgtaggtggctttttaagtccgccgtcaaatcccagggctcaactctggacaggcggtggaaactaccaagctggagtacggta
ggggcagagggaatttccggtggagcggtgaaatgcgtagagatcggaaagaacaccaacggcgaaagcactctgctgggccgacactgaca
ctgagggacgaaagctaggggagcgaatg

>04be702b895fe5ed0568344daf564276

tagggaatcttccgcaatgggcgaaagcctgacggagcaacgccgcgtgagtgatgaaggtcttcggatcgtaaaactctgttattagggaaga
acatatgtgtaagtaactgtgcacatcttgacggtacctaatcagaaagccacggctaactacgtgccagcagccgcggtaatacgtaggtggc
aagcgttatccggaattattgggcgtaaagcgcgcgtaggcggtttttttaagtctgatgtgaaagcccacggctcaaccgtggagggtcattgga
aactggaaaacttgagtgcagaagaggaaagtggaattccatgtgtagcggtgaaatgcgcagagatatggaggaacaccagtggcgaaggc
gactttctggtctgtaactgacgctgatgtgcgaaagcgtggggatcaaaca

>063ec35700f6d12e12c9dda2991550e1

tggggaatcttgcgcaatgggcgaaagcctgacgcagcaacgccgcgtgcgggatgaaggccctcgggtcgtaaaccgctttcgacagggaag
aaaatgacggtacctgtagaagaagccccggccaactacgtgccagcagccgcggtaatacgtagggggcgagcgttgtccggatttattggg
cgtaaagagctcgtaggcggttaactaagtcagatgtgaaaactcagggcctaactctgagcctgcatttgatactggtttgactagagtcaagt
aggggagtgtggaattcccagtgtagcggtgaaatgcgcagatattgggaggaacaccagcagcgaaggcggcactctgggcttgtactgacg
ctgaggagcgaaagcatgggtagcaaaca

>06f8df735f9242b4613f84c5c6cbab5c

tggggaatattgcgcaatgggcgaaagcctgacgcagcgacgccgcgtgagggatgacggtcttcggattgtaaacctctttcagcagggacga
aattgacggtacctgcagaagaagcaccggctaactacgtgccagcagccgcggtaatacgtagggtgcaagcgttgtccggatttattgggcg
taaagagctcgtaggcggtttgtcgcgtcggatgtgaaaccccaaggcttaaccttgggcctgcattcgatacgggcagactagaggatggcag
gggaaactggaattcctggtgtagcggtggaatgcgcagatatcaggaggaacaccggtggcgaaggcgggtttctgggccattactgacgctg
aggagcgaaagcgtggggagcaaaca

>073e48b2f66e03194e0f964f0686864f

tagggaatcttccgcaatggacgaaagtctgacggagcaacgccgcgtgtatgaagaaggttttcggatcgtaaagtactgttgttagagaaga
acaaggataagagtaactgcttgtcccttgacggtatctaaccagaaagccacggctaactacgtgccagcagccgcggtaatacgtaggtggc
aagcgttgttcggaattactgggcgtaaagcgcgcgtaggcggactggtcagttgggggtgaaatcccgaggcttaacctcggaactgccttca
atactgccagtcttgagatcggaagaggtgagtggaattcctagtgtagaggtgaaattcatagatattaggaagaacaccagtggcgaaggcg
gctcactggtccgatactgacgctgaggtgcgaaagcgtggggagcgaaca

>075d92a9826e0ac375cd0d532f68499f

tggggaatcttgcgcaatgggcgaaagcctgacgcagcaatgccgcgtgagggaagaaggtcttaggattgtaaacctctttcagcagggaag
aaaacagacggtacctgcagaagaagccccggctaactacgtgccagcagccgcggtaatacgtagggggcgagcgttgtccggaatcattgg
gcgtaaagagcgtgtaggcggactggaaggtctggtgtgaaagcccggggctcaaccccggagggtcaccggaaaccgacagtcttgagtccg
gaagaggagagtggaattcctggtgtagcggtggaatgcgcagatatcaggaggaacaccaatggcgaaggcagctctctggtacgtgactga
cgctgagacgcgaaagcgtggggagcgaaca

>0760e3bc01b20d91c54587bcd8064543

tggggaattttccgcaatgggcgaaagcctgacggagcaatgccgcgtggaggtggaaggcctacgggtcgtcaacttcttctctcggagaaga
aacaatgacggtatctgaggaataagcatcggctaactctgtgccagcggccgcggtaagacagaggatgcaagcgttatccggaatgattgg
gcgtaaagcgtctgtaggtggcttttcaagtccgccgtcaaatcccagggctcaaccctggacaggcggtggaaactaccaagctggagtacgg
taggggcagagggaatttccggtggagcggtgaaatgcattgagatcggaaagaacaccaacggcgaaagcactctgctgggccgacactga
cactgagagacgaaagctaggggagcaaatg

>07c3160551922faa7be5740ea3adc189

tggggaattttggacaatgggggcaaccctgatccagcaatgccgcgtgtgtgaagaaggccttcgggttgtaaagcacttttgtccggaaaga
aatcgcacttactaatattaggtgtggatgacggtaccggaagaataaggaccggctaactacgtgccagcagccgcggtaatacgtagggtcc
aagcgttaatcggaattactgggcgtaaagcgtgcgcaggcggttgtgcaagaccgatgtgaaatccccgggcttaacctgggaattgcattggt
gactgcacggctagagtgtgtcagaggggggtagaattccacgtgtagcagtgaaatgcgtagagatgtggaggaataccgatggcgaaggca
gccccctgggataacactgacgctcatgcacgaaagcgtggggagcaaaca

>08b5edd3f9f582d34a2f26eb8fa5819d

tggggaattttccgcaatgggcgaaagcctgacggagcaatgccgcgtggaggtggaaggcctacgggtcgtcaacttcttttctcggagaaga
aacaatgacggtatctgaggaataagcatcggctaactctgtgccagcagccgcggtaagacagaggatgcaagcgttatccggaatgattgg
gcgtaaagcgtctgtaggtggcttttcaagtccgccgtcaaatcccagggctcaaccctggacaggcggtggaaactaccaagctggagtacgg
taggggcagagggaatttccggtggagcggtgaaatgcattgagatcgtaaagaacaccaacggcgaaagcactctgctgggccgacactgac
actgagagacgaaagctaggggagcaaatg

>090a919b28608a194dfe6738a8a3fda5

tggggaattttccgcaatgggcgaaagcctgacggagcaatgccgcgtggaggtggaaggcctacaggtcgtcaacttcttttctcggagaaga
aacaatgacggtatctgaggaataagcattggctaactctgtgccagcagccgcggtaagacagaggatgcaagcgttatccggaatgattgg
gcgtaaagcgtctgtaggtggcttttcaagtccgccgtcaaatcccaatggctcaaccctggacaggcggtggaaactaccaagctggagtatg
gtaggggcagagggaatttccggtggagcggtgaaatgcattgagatcggaaagaacaccaacggcgaaagcactctgctgggccgacactg
acactgagagacgaaagctaggggagcaaatg

>09206a00841a5c45ee6369cdc14800b0

tggggaattttccgcaatgggcgaaagcctgacggagcaatgccgcgtggaggtggaaggcctacgggtcgtcaacttcttttctcggagaaga
aacaatgacggtatctgaggaataagcatcggctaactctgtgccagcagccgcggtaagacagaggatgcaagcgttatccggaatgattgg
gcgtaaagcgtctgtaggtggcttttcaagtccgccgtcaaatcccagggctcaaccctggacaggcggtggaaactaccaagctggagtacgg
taggggcagagggaatttccggtggagcggtgaaatgcattgagatcggaaagaacaccaacggcgaaagcactctgctgggccgacactga
cactgagagacgaaagctaggggagcaaatgggattagataccctggtagt

>0a1582d42cf13d17b5c34eb87d9edf8a

tggggaatattgcacaatggaggaaactctgatgcagcaacgccgcgtggaggatgacacatttcggtgcgtaaactccttttatatgggaagat
aatgacggtaccatatgaataagcaccggctaactccgtgccagcagccgcggtaatacggagggtgcgagcgttatccggaatcattgggttt
aaagggttcgtaggcgggtcaataagtcagaggtgaaatcttgcagcttaactgtaaaatggcctttgatactgttgaccttgaattactgtgaag
tggttagaatatgtagtgtagcggtgaaatgcttagagattacatagaataccgattgcgaaggcagatcactaatagtatattgacgctgatga
acgaaagcgtgggtagcgaacg

>0a1f5ed70dcb37a86924dbd5fde981af

tggggaatattggacaatgggggcaaccctgatccagccatgccgcgtgtgtgaagaaggcctttttggttgtaaagcactttaagcagggagga
gaggctaatggttaatacccattagattagacgttacctgcagaataagcaccggctaactctgtgccagcagccgcggtaatacagagggtgc
gagcgttaatcggaattactgggcgtaaagcgagtgtaggtggctcattaagtcacatgtgaaatccccgggcttaacctgggaactgcatgtga

tactggtggtgctagaatatgtgagagggaagtagaattccaggtgtagcggtgaaatgcgtagagatctggaggaataccgatggcgaaggc
agcttcctggcataatattgacactgagattcgaaagcgtgggtagcaaaca

>0a60c01d1b75bb107d1aa0e919e048b6

tggggaattttccgcaatgggcgaaagcctgacggagcaataccgcgtgagggatgacggcctatgggttgtaaacctctttttcagggagga
ataaatgacgtgtacctgaagaataagcatcggctaactccgtgccagcagccgcggtaagacggaggatgcaagtgttatccggaatcactg
ggcgtaaagcgtctgtaggtggtttaataagtcaactgttaaatcttgaggctcaacctcaaaatcgcagtcgaaactattagactagagtatagt
aggggtaaagggaatttccagtggagcggtgaaatgcgtagatattggaaggaacaccgatggcgaaggcactttactgggctattactaaca
ctcagagacgaaagctagggtagcaaatg

>0a7a72cf0cbf9f815d62c85dfa0b269a

tggggaatcttagacaatgggggaaaccctgatctagccatgccgcgtgatcgatgaaggccttagggttgtaaagatctttcagatgggaaga
taatgacggtaccatcagaagaagccccggctaactccgtgccagcagccgcggtaatacggagggggctagcgttattcggaattactgggcg
taaagcgcacgtaggcggatcagaaagtcagaggtgaaatcccagggctcaaccttggaactgcctttgaaactcctggtcttgaggtcgagag
aggtgagtggaattccgagtgtagaggtgaaattcgtagatattcggaggaacaccagtggcgaaggcggctcactggctcgatactgacgctg
aggtgcgaaagcgtggggagcaaaca

>0bea95fe73cab86e2f8520bd5095878f

tgaggaattttccgcaatgggcgaaagcctgacggagcaataccgcgtggaggatgacggcctgtgggtcttaaactccttttcttagagaaga
agttctgacggtatctaaggaataagcatcggctaactccgtgccagcagccgcggtaatacgggggatgcaagcgttatccggaatgattggg
cgtaaagcgtctgtaggtggctttccaagtcaactgtcaaaaatcagggcttaaccttgagccggcagttgaaactatgaagcttgagttaggta
ggggcagagggaattcctagtgtagcggtgaaatgcgtagatattaggaagaacaccaatcgcgaaagcactctgctgggccatgactgacac
tgagagacgaaagctaggggagcgaatgggattagataccccagtag

>0cf3a2c867b5632992f47d06a363fa6e

tagggaatcttccacaatggacgcaagtctgatggagcaacgccgcgtgagtgaagaaggttttcggatcgtaaagctctgttgttggtgaaga
aggatagaggtagtaactggcctttatttgacggtaatcaaccagaaagtcacggctaactacgtgccagcagccgcggtaatacgtaggtggc
aagcgttgtccggatttattgggcgtaaagcgagcgcaggcggaatgataagtctgatgtgaaagcccacggctcaaccgtggaactgcatcgg
aaactgtcattcttgagtgcagaagaggagagtggaactccatgtgtagcggtggaatgcgtagatatatggaagaacaccagtggcgaaggc
ggctctctggtctgcaactgacgctgaggctcgaaagcatgggtagcgaaca

>0d60e090dd8bc66c7a72007827a11981

tggggaattttggacaatgggcgaaagcctgatccagcaatgccgcgtgcaggatgaaggccttcgggttgtaaactgcttttgtacggaacga
aacggttctttctaataaagagagctaatgacggtaccgtaagaataagcaccggctaactacgtgccagcagccgcggtaatacgtagggtgc
aagcgttaatcggaattactgggcgtaaagcgtgcgcaggcggttatgtaagacagttgtgaaatccccgggctcaacctgggaattgcatctgt
gactgcatagctagagtacggtagagggggatggaattccgcgtgtagcagtgaaatgcgtagatatgcggaggaacaccgatggcgaaagc
aatcccctggacctgtactgacgctcatgcacgaaagcgtggggagcaaaca

>0ee0776f049d07206e50c7bf26ea15e2

taaggaatattggacaatggccgcaaggctgatccagccatccagcgtgcaggaagaaggccctatgggttgtaaactgcttttatcagggaat
aaacctctggattcactccggagctgaaggtacctgaagaataagcaccggctaactccgtgccagcagccgcggtaatacggagggtgcgag
cgttatccggaattactgggtttaaagggtgcgtaggcggctttgtaagtcaggagtgaaagtttgcggctcaaccgtaaaattgcttctgatact
gcatggctagaattaggatgaggtcagcggaatgtggcatgtagcggtgaaatgcatagatatgccatagaacaccaattgcgaaggcagctg
gctagacctggattgacgctgaggcacgaaagcgtggggagcgaaca

>0ee9aeb8bb3ebbccdb9cfebf3afa610a

tagggaatcttccgcaatggacgaaagtctgacggagcaacgccgcgtgtatgaagaaggttttcggatcgtaaagtactgttgttagagaaga
acaaggataagagtaactgcttgtcccttgacggtatctaaccagaaagccacggctaactacgtgccagcagccgcggtaatacgtaggtggc
aagcgttgtccggatttattgggcgtaaagcgcgcgcaggcggtcttttaagtctgatgtgaaagcccccggcttaaccggggagggtcattgga
aact

>0f56078109a37d2c8d5d5fb471f699e8

tggggaatattggacaatgggcgaaagcctgatccagccatgccgcgtgtgtgaagaaggtcttcggattgtaaagcactttaagttgggagga
agggttgtagcttaatacgctgcaaccttgacgttaccaacagaataagcaccggctaactctgtgccagcagccgcggtaatacagagggtgc
aagcgttaatcggaattactgggcgtaaagcgcgcgtaggtggcaaagtaagatgggtgtgaaatccccgggctcaacctgggaactgcatcc
ataactgcttcgctagagtacggtagagggtagtggaatttcctgtgtagcggtgaaatgcgtagatataggaaggaacaccagtggcgaaggc
gactacctggactgatactgacactgaggtgcgaaagcgtggggagcaaaca

>101637aba474a6614786486ad42acb11

tagggaatcttcggcaatggacggaagtctgaccgagcaacgccgcgtgagtgaagaaggttttcggatcgtaaagctctgttgtaagagaaga
acgagtgtgagagtggaaagttcacactgtgacggtatcttaccagaaagggacggctaactacgtgccagcagccgcggtaatacgtaggtcc
cgagcgttgtccggatttattgggcgtaaagcgagcgcaggcggttagataagtctgaagttaaaggctgtggcttaaccatagtacgctttgga
aactgtttaacttgagtgcaagaggggagagtggaattccatgtgtagcggtgaaatgcgtagatatatggaggaacaccggtggcgaaagcg
gctctctggcttgtaactgacgctgaggctcgaaagcgtggggagcaaaca

>10dcede07fbb5f3a7a229d97beae316e

tggggaattttccgcaatgggcgaaagcctgacggagcaatgccgcgtggaggtggaaggcctacgggtcgtcaacttcttttctcggagaaga
aacaatgacggtatttgaggaataagcatcggctaactctgtgccagcagccgcggtaagacagaggatgcaagcgttatccggaatgattgg
gcgtaaagcgtctgtaggtggcttttcaagtccgccgtcaaatcccagggctcaaccctggacaggcggtggaaactaccaagctggagtacgg
taggggcagagggaatttccggtggagcggtgaaatgcattgagatcggaaagaacaccaacggcgaaagcactctgctgggccgacactga
cactgagagacgaaagctaggggagcaaatg

>11a1aed0371e3bdfb03d87bdb449a4c4

tggggaatcttggacaatgggcgaaagcccgatccagcaatatcgcgtgagtgaagaagggcaatgccgcttgtaaagctctttcgtcgagtgc
gcgatcatgacaggactcgaggaagaagtcccggctaactccgtgccagcagccgcggtaagacgggggggcaagtgttcttcggaatgact
gggcgtaaagggcacgtaggcggtgaatcgggttgaaagttaaagtcgccaaaaactggtggaatgctctcgaaaccaattcacttgagtgag
acagaggagagtggaatttcgtgtgtaggggtgaaatccgcagatctacgaaggaacgccaaaagcgaaggcagctctctgggtccctaccga
cgctggagtgcgaaagcatggggagcgaacg

>120540d18ed88a6b75d9f796dce95beb

tagggaatcttccgcaatggacgaaagtctgacggagcaacgccgcgtgtatgaagaaggttttcggatcgtaaagtactgttgttagagaaga
acaaggataagagtaactgcttgtcccttgacggtatctaaccagaaagccacggctaactacgtgccagcagccgcggtaatacgtaggtggc
aagcgttgtccggatttattgggcgtaaagcgcgcgcaggcggtcttttaagtctgatgtgaaagcccccggcttaaccggggagggtcattgga
aactggaagactggagtgcagaagaggagagtggaattccacgtgtagcggtgaaatgcgtagatatcgggaggaacaccagtggcgaaggc
ggcctcctggaccaagactgacgctgaggtgcgaaagcgtggggagcaaaca

>123b5897f78e93e51758e1a210ec6a85

tagggaatcttccgcaatggacgaaagtctgacggagcaacgccgcgtgtatgaagaaggttttcggatcgtaaagtactgttgttagagaaga
acaaggataagagtaactgcttgtcccttgacggtatctaaccagaaagccacggctaactccgtgccagcagccgcggtaatacggagggtgc
aagcgttaatcggaattactgggcgtaaagcgcgcgtaggcggcttgttaagtcggatgtgaaatccccgggctcaacctgggaactgcattcg
atactggctggctagagtgtggtagagggaagtggaattccaggtgtagcggtgaaatgcgtagatatctgggggaacatcagtggcgaaggc
ggcttcctggaccaacactgacgctgaggcgcgaaagcgtggggagcaaaca

>128854112a231f5cd34bceb6b0446ca9

tggggaatattgcgcaatgggcgaaagcctgacgcagcgacgccgcgtgagggatgaaggccttcgggttgtaaacctctttcagcagggacg
aagcgaaagtgacggtacctgcagaagaagcaccggctaactacgtgccagcagccgcggtaatacgtagggtgcaagcgttgtccggattta
ttgggcgtaaagagctcgtaggcggtctgtcgcgtcggatgtgaaaacttggggctcaaccctgagcttgcattcgatacgggcagactagagg
atggcaggggagactggaactcctggtgtagcggtggaatgcgcagatatcaggaagaacaccggtggcgaaggcgggtctctgggccattac
tgacgctgaggagcgaaagcgtggggagcaaaca

>12d6b1ac93111f354e16a73df44f9fce

taaggaatattggacaatggaggcaactctgatccagccatgccgcgtgaaggattaaggccctatgggttgtaaacttcttttagacgggacaa
aacccctttacgtgtaaagggttgatggtactgtcagaataagcatcggctaacttcgtgccagcagccgcggtaatacgaaggatgcaagcgtt
atccggaattattgggtttaaagggtgcgtaggcggactaataagtcagtggtgaaagcctgcagcttaactgcagaattgccgttgatactgtta
gtcttgagtacagttgaggtaggcggaatgtgtcatgtagcggtgaaatgcttagatatgacacagaacaccgattgcgaaggcagcttgctaa
gctgtaactgacgctgaggcacgaaagcgtggggagcaaaca

>12da874e4a73d0a98c1ea0a23957bd44

tggggaatattggacaatgggcgaaagcctgatccagccatgccgcgtgagtgatgaaggtcttcggatcgtaaagctctgtgggaagggacg
aaaaagcctaacctaatacgttaggtcttgacggtaccttcttagcaagcaccggctaactccgtgccagcagccgcggtaatacggagggtgc
aaacgttgctcggaattattgggcgtaaagcgcacgtaggcggtccgctaagtcggatgtgaaagccctcggcttaaccgaggaagtgcatccg
aaactgacgggctagagtacggaagagggtttcggaattcccggtgtagaggtgaaattcgtagatatcgggaggaacaccagtggcgaaggc
ggaaacctgggccgatactgacgctgaggtgcgaaagcgtggggagcaaaca

>13008322aeaf6d05249ad9682125b366

tggggaatattgcacaatgggcgcaagcctgatgcagccatgccgcgtgtgtgaagaaggccttcgggttgtaaagcactttcagtcgtgagga
aggtggtagtgttaatagcactatcatttgacgttagcgacagaagaagcaccggctaactccgtgccagcagccgcggtaatacggagggtgc
gagcgttaatcggaattactgggcgtaaagcgcatgcaggtggtttgttaagtcagatgtgaaagcccggggctcaacctcggaattgcatttga
aactggcagactagagtactgtagagggggggtagaatttcaggtgtagcggtgaaatgcgtagagatctgaaggaataccggtggcgaaggcg
gccccctggacagatactgacactcagatgcgaaagcgtggggagcaaaca

>135b1d618f7e68209c8d7e135e4b43b9

taaggaatattggtcaatggacggaagtctgaaccagccatcccgcgtgcaggatgaaggtcctatggattgtaaactgcttttagcagagacg
aagacccatcatttatgatggagtgacggtatctgcggaataagcaccggctaactccgtgccagcagccgcggtaatacggagggtgcaagc
gttatccggaatcactgggtttaaagggtgcgtaggcggagaattaagtcagtggtgaaatcctgtcgcttaacgatagaactgccattgatact
gattttcttgaattgggttgaggttagcggaatgtgacatgtagcggtgaaatgcatagatatgtcatggaacaccaattgcgaaggcagctaac
tgggccttgattgacgctgaggcacgaaagcgtgggtagcgaaca

>13a5db768c0e55c55824b0d5980fbf90

tcgagaatcttcggcaatgggggaaaccctgaccgagcgacgccgcgtgcgggatgaaggccctcgggttgtaaaccgctgtcgtaagggaac
aaatctatgagggtactctctcatagttgagtgatcttaggaggaaggacgggctaagttcgtgccagcagccgcggtaagacgaaccgtccga
acgttattcggtatcactgggcttaaagagttcgtaggcggcccgacaggtgaggtgtgaaatccctcggctcaaccgaggaattgcgcttcaaa
ccgtcaggcttgagggagatagaggtgagcggaacagatggtggagcggtgaaatgcgttgatatcatctggaacaccggtggcgaaagcgg
ctcactggatcttttctgacgctgaggaacgaaagctagggtagcgaacg

>14a7f75d4fa5795e78aec05501b5d4c1

tggggaatattgcgcaatgggcgaaagcctgacgcagccacgccgcgtgtgtgatgaaggccttcgggttgtaaagcactgtcgggagggacg
aagaaatgacggtacctcccaagaaagcaccggctaaccctgtgccagcagccgcggtaatacagggggtgcaagcgttgctcggaatcactg
ggcgtaaagcgtacgcaggcggaccgttaagtcggatgtgaaagcccggggctcaaccccggaacggcattcgaaactggcggtcttgaatac
gggagaggtgaatggaattcctggtgtagaggtgaaattcgtagatatcaggaggaacatcggtggcgaaagcggttcactggcccgatattga
cgctcatgtacgaaagcgtggggagcaaaca

>15e409cec6365290b44b6ac6c6aad668

tggggaatattggacaatgggggaaccctgatccagccatgccgcgtgtgtgaagaaggccttttggttgtaaagcactttaagcgaggagga
ggctacctagattaatactttaggatagtggacgttactcgcagaataagcaccggctaactctgtgccagcagccgcggtaatacagagggtg
cgagcgttaatcggatttactgggcgtaaagcgtgcgtaggcggccaattaagtcaaatgtgaaatccccgagcttaacttgggaattgcattcg
atactggttggctagagtatgggagaggatggtagaattccaggtgtagcggtgaaatgcgtagagatctggaggaataccgatggcgaaggc
agccatctggcctaatactgacgctgaggtacgaaagcatggggagcaaaca

>168c7040965c754f79d642c92f79809f

tagggaattttccgcaatgggcgaaagcctgacggagcaatgccgcgtggagggtgaaggcctattgggttgttaactccttttcccagagaaga
aacctgacggtatctggggaataagcatcggctaactccgtgccagcagccgcggtaatacgggggatgcaagcgttatccggaattattgggc
gtaaagagtccgtaggttgttatcaaagcctattgttaaatatcaaggcttcaccttgaatctgcagtaggaacctgataacttgagtgcggtagg
agtaggggggaattcccggtgtagcggtgaaatgcgtagatatcgggaagaacaccggaggcgaaagcgccctactaggccgacactgacact
aagggacgaaagctaggggagcaaacg

>19a9e24a3794be248d52e98ec10e1c6b

tggggaatattggacaatgggcgcaagcctgatccagccatgccgcgtgagtgatgacggccttagggttgtaaagctctttcaacggtgaagat
aatgacggtaaccgtagaagaagccccggctaacttcgtgccagcagccgcggtaatacgaagggggctagcgttgttcggaattactgggcgt
aaagcgcacgtaggcggatacttaagttaggggtgaaatcccgaggctcaacctcggaactgcctttgatactgggtatctcgagtatggtaga
ggtaagtggaactccgagtgtagaggtgaaattcgtagatattcggaagaacaccagtggcgaaggcggcttactggaccattactgacgctga
ggtgcgaaagcgtggggagcaaaca

>19b7c685c65baea7222e6b58c5ec2469

tgaggaattttggacaatgagctaacgcttgatccagcaatactacgtgggtgatgaaggctaatttagttgtaaaaccctttttacagtgacgat
aatgacggtagctgtagaataagccccggcaaatttcgtgccagcagccgcggttatacgagaggggctagtgttattcaaaattattgggcgta
aagggtacgtaggtggaaaaacaagttaaaaatcaaatatttaggcttaaccttaatatagtttttaatactatttttctagagtaaagtagagga
tagtagaatttctaatgtaacgatggaatgtttatatattagaaggaataccgtagcgaaggcagctatctggacttatactgacactgaggtac
gaaagcataagtagcaaatg

>1a24c2a441deac742f6c047eb37d5887

tagggaatcttccgcaatggacgaaagtctgacggagcaacgccgcgtgtatgaagaaggttttcggatcgtaaagtactgttgttagagaaga
acaaggataagagtaactgcttgtcccttgacggtatctaaccagaaagccacggctaactacgtgccagcagccgcggtaatacggagggtgc
aagcgttatccggaatcactgggtttaaagggtgcgtaggcggcgctataagtcagaggtgaaaggccaccgcttaacggtgggactgcctttg
atactgtagtgcttgaatcaggttgaggtcggcggaatgtgacatgtagcggtgaaatgcttagatatgtcatagaacaccaattgcgaaggcag
ctggctagaccttgattgacgctgaggcacgaaagcgtggggagcgaaca

>1a2ba4587ee37d8984eab739d1445c19

tggggaattttccgcaatgggcgaaagcctgacggagcaatgccgcgtggaggtggaaggcctacgggtcgtcaacttcttttctcggagaaga
aacaatgacggtatctgaggaataagcatcggctaactctgtgccagcagccgcggtaagacagaggatgcaagcgttatccggaatgattgg
gcgtaaagcgtctgtaggtggcttttcaagtccgccgtcaaatcccagggctcaaccctggacaggcggtggaaactactaagctggagtacgg
taggggcagagggaatttccggtggagcggtgaaatgcattgagatcggaaagaacaccaacggcgaaagcactctgctgggccgacactga
cactgagagacgaaagctaggggagcaaatg

>1a48aa666133ff5cf4bcabe1fb2830c1

tggggaatattgcacaatgggcggaagcctgatgcagcgacgccgcgtgagggatgacggccttcgggttgtaaacctctttcgaccctgacga
agcgtgagtgacggtagggggagaagaagcaccggccaactacgtgccagcagccgcggtaagacgtagggtgcgagcgttgtccggatttat
tgggcgtaaagagctcgtaggcggctcgtcgcgtcgaccgtgaaaacttggggcttaaccctaagcttgcggttgatacgggcgggctagagttc

ggtaggggagactggaattcctggtgtagcggtgaaatgcgcagatatcaggaggaacaccggtggcgaaggcgggtctctgggccgatactg
acgctgaggagcgaaagcgtggggagcgaaca

>1b404f71123067723c2fa8772ff67647

tgaggaatattgggcaatggaggcaactctgacccagccatgccgcgtgcaggaagactgccctatgggttgtaaactgcttttatacaggaag
aaacagttctacgtgtagaaccttgacggtactgtaagaataaggaccggctaactccgtgccagcagccgcggtaatacggggggatgcaagc
gttatccggaatgattgggcgtaaagcgtctgtaggtggcttttcaagtccgccgtcaaatcccagggctcaaccctggacaggcggtggaaact
accaagctggagtacggtaggggcagagggaatttccggtggagcggtgaaatgcattgagatcggaaagaacaccaacggcgaaagcactc
tgctgggccgacactgacactgagagacgaaagctaggggagcaaatg

>1be4852d442a272814aaef6d6f981e99

ccaccttacctacggggggctgcagtgaggaattttccgcaatgggcgaaagcctgacggagcaataccgcgtggaggatgacggcctgtgggt
cttaaactccttttcttagagaagaagttctgacggtatctaaggaataagcatcggctaactccgtgccagcagccgcggtaatacggggggatg
caagcgttatccggaatgattgggcgtaaagcgtctgtaggtggctttccaagtcaactgtcaaaaatcagggcttaaccttgagcc

>1c1cf920678e035ad553becf12c8205a

tgaggaattttctgcaatgggcgaaagcctgacagagcaatatcgcgtgagggaggaaggcctacgggttgtaaacctcttttctcaaggagga
aactatgacgttacttgaggaataagcatcggctaaccctgtgccagcagccgcggtaatacaggggatgcaagtgttatccggaatcattggg
cgtaaagcgtctgcaggctggcttttaagtcttttgttaaagttctaggctcaacctagaaaatgcaaaggaaactaaaagccttgagtatggtag
aggtaaagggaatttccagtggagcagtgaaatgcttagatattggaaagaacaccaacggcgaaggcactttactgggccagcactgacgct
gagagacgaaagctaggggagcaaacg

>1d69806f9c912f325cc581d0b5d122b8

tggggaattttggacaatgggggcaaccctgatccagccatgccgcgtgagtgaagaaggccttcgggttgtaaagctctttcggcggggaaga
aatcgcatctcctaatacggggtgtggatgacggtacccgaataagaagcaccggctaactacgtgccagcagccgcggtaatacgtagggtgc
gagcgttaatcggaattactgggcgtaaagcgtgcgcaggcggttttgtaagacagatgtgaaatccccgggctcaacctgggaactgcatttgt
gactgcaaggctagagtatggcagagggggggtggaattccacgtgtagcagtgaaatgcgtagagatgtggaggaacaccgatggcgaaggc
agccccctgggccaatactgacgctcatgcacgaaagcgtggggagcaaaca

>1d69dd92369c5a1b4620c86f79748f41

tggggaattttccgcaatgggcgaaagcctgacggagcaatgccgcgtggaggtagaggctcctgggtcatgaacttcttttcccggagaagaa
aaaatgacggtatccggggaataagcatcggctaactctgtgccagcagccgcggtaagacagaggatgcaagcgttatccggaatgattggg
cgtaaagcgtctgtaggtggctttttaagttcgctgtcaaataccagggctcaaccctggacaggtggtgaaaactactaagctagagtacggta
ggggcagagggaatttccggtggagcgatgaaatgcgtagagatcggaaggaacaccaacggcgaaagcactctgctgggccgacactgaca
ctgagagacgaaagctaggggagcgaatg

>1dfe95826824dbd649020bf872431102

tggggaatttttcgcaatgggcgaaagcctgacggagcaatgccgcgtggaggtggaaggcctacggttcgtcaacttcttttctcggagaagaa
acaatgacggtatctgaggaataagcatcggctaactctgtgccagcagccgcggtaagacagaggatgcaagcgttatccagaatgattggg
cgtaaagcgtctgtaggtggcttttcaagtccgccgtcaaatcccagggctcaaccctggatatgcgacggaaactaccaagctggagtacggta
ggggcagagggaatttccggtggagcggtgaaatgcattgagatcggaaagaacaccaacggtgaaagcactctgcggggtcgacactgaca
ctaagagacgaaagctaggggagcaaatg

>1e2e0b8dc088d288945341e0dde5439a

tggggaattttccgcaatgggcgaaagcctgacggagcaatgccgcgtggaggtagaggcccctgggtcatgaacttcttttcccggagaagaa
aaaatgacggtatccggggaataagcatcggctaactctgtgccagcagccgcggtaagacagaggatgcaagcgttatccggaatgattggg
cgtaaagcgtctgtaggtggctttttaagttcgctgtcaaataccagggctcaaccctggacaggtggtgaaaactactaagctagagtacggta

ggggcagagggaatttccggtggagcgatgaaatgcgtagagatcggaaggaacaccaacggcgaaagcactctgctgggccgacactgaca
c

>1e49ea05196d01db42590d502dd85e3a

tggggaattttggacaatgggcgcaagcctgatccagccatgccgcgtgcgggaagaaggccttcgggttgtaaaccgcttttgtcagggaaga
aaaggttctggctaatacctgggactcatgacggtacctgaagaataagcaccggctaactacgtgccagcagccgcggtaatacgtagggtgc
aagcgttaatcggaattactgggcgtaaagcgtgcgcaggcggttatgcaagacagaggtgaaatccccgggctcaacctgggaactgcctttg
tgactgcatggctagagtacggtagagggggatggaattccgcgtgtagcagtgaaatgcgtagatatgcggaggaacaccgatggcgaaggc
aatcccctggacctgtactgacgctcatgcacgaaagcgtggggagcaaaca

>1ebe0ee8d42dc4f5fe388e894d6c303c

tggggaattttccgcaatgggcgaaagcctgacggagcaatgccgcgtggaggtagaaggcccacgggtcgtgaacttcttttcccggagaaga
agcaatgacggtatctggggaataagcatcggctaactctgtgccagcagccgcggtaatacagaggatgcaagcgttatccggaatgattggg
cgtaaagcgtctgtaggtggctttttaagtccgccgtcaaatcccagggctcaactctggacaggcggtggaaactaccaagctggagtacggta
ggggcagagggaatttccggtggagcggtgaaatgcgtagagatcggaaagaacaccaacggcgaaagcactctgctgggccgacactgaca
ctgagagacgaaagctaggggagcgaatg

>1f6ca6657f9b8fa43db47a0f67ee8d98

tggggaatcttggacaatgggcgaaagcccgatccagcaatattgcgtgggtgaagaagggcaattccgcttgtaaagctctttcgtcgagtgcg
cgatcatgacaggactcgaggaagaagccccggctaactccgtgccagcagccgcggtaagacgggggggcaagtgttattcggaatgacta
ggcgtaaagggcacgtaggcggtgaatcgggttggaagtgaaagtcgccaacaattggcggggtgctttcggaaccaattcacttgagtgagat
aggggagagtggaatttcgtgtgtagggatcaaatccggagatctacgaaggaacgccaaaagcgaaggcagctctctgggtctacactgacg
ctggggtgcgaaagcatggggagcaaaca

>1fcbb6ac70fc87b66052a7c74bf363b3

tggggaatattggacaatgggcgcaagcctgatccagcaataccgcgtgaatgatgaaggccttcgggttgtaaagttcttttagtagggaagat
aatgacggtacctacagaaaaagcaccggctaacttcgtgccagcagccgcggtaatacgaagggtgctagcgttgttcggaatcactgggcgt
aaagcgtgcgtaggcggcttggtaagtcagttgtgaaagcccggggctcaacctcggaattgcagctgaaactacctcgctagagtatggttgg
ggatagtagaattcctagtgtaggggtgaaatccgtagatattaggaggaataccggtggcgaaggcgactatctaagccattactgacgctga
ggtacgaaagcgtggggagcaaaca

>2041b05a2f9eff7bf328119b31c4dd67

tggggaatattggacaatgggcgcaagcctgatccagcaatgccgcgtgggtgaagacggcctgcgggttgtaaagccctttggcggggagg
aacggcgtcgcgcgaacagggcggcgctctgacgttacccgcgaaacaagcaccggctaactccgtgccagcagccgcggtaatacggagggt
gcgagcgttaatcggaattactgggcgtaaagcgtgcgtaggcggtccggtcagtcagccgtgaaagccccgggctcaacctgggaacggcgg
ttgagacggccggactggagtgggctagaggatcgtggaattccgggtgtagcggtgaaatgcgtagagatccggaggaacaccgatggcga
aggcagcggtctggggccacactgacgctgaggcacgaaagcgtggggagcaaaca

>2123fabe9329206256ba554350586bcd

taaggaatattggacaatggacggaagtctgatccagccatgccgcgtgaaggatgaaggcgctcagcgttgtaaacttcttttggatggggac
aaaccacttcatttatggggtgctgagggtaccatcagaataagcaccggctaactccgtgccagcagccgcggtaatacggagggtgcaagcg
ttatccggaatcactgggtttaaagggtgcgtaggcggtctgataagtcagttgtgaaattcctcggcttaaccgagtggactgcgattgatactg
ttagacttgaatcaggttgaggtaggcggaatgtgacatgtagcggtgaaatgcttagatatgtcatagaacaccgattgcgaaggcagcttgct
gggcttcgattgacgctgaggcacgaaagcgtggggagcaaaca

>2164e40cb22143f4fb8ebe25ee8bb325

tagggaatcttccgcaatggacgaaagtctgacggagcaacgccgcgtgagtgaagaaggttttcggatcgtaaaactctgttgtcagagaaga acaagttggagagtaactgctccagccttgacggtatctgaccagaaagccacggctaactacgtgccagcagccgcggtaatacgtaggtggc aagcgttgtccggatttattgggcgtaaagcgagcgcaggcggttccttaagtctgatgtgaaagcccacggctcaaccgtggaaggtcattgga aactggggaacttgagtgcagaagaggagagtggaattccatgtgtagcggtgaaatgcgtagatatatggaggaacaccagtggcgaaggc gactctctggtctgtaactgacgctgaggctcgaaagcgtggggagcaaaca

>216c57de85aa19814275de7ff3264e1c

tagggaatcttccgcaatggacgaaagtctgacggagcaacgccgcgtgtatgaagaaggccttcgggttgtaaagcactttcagtcgtgagga aggtggtagtgttaatagcactatcatttgacgttagcgacagaagaagcaccggctaactccgtgccagcagccgcggtaatacggagggtgc gagcgttaatcggaattactgggcgtaaagcgcacgcaggcggtttgttaagctagatgtgaaagccccgagctcaacttgggatggtcatttag aactggcagactagagtcttggagaggggagtggaattccaggtgtagcggtgaaatgcgtagatatctggaggaacatcagtggcgaaggcg actccctggccaaagactgacgctcatgtgcgaaagtgtgggtagcgaaca

>224cd3b2e0e33d8ecd351ab6da333d50

tggggaatattgcacaatgggcgcaagcctgatgcagcgacgccgcgtgagggatggaggccttcgggttgtaaacctcttttatcggggagca agcgtgagtgagtttacccgttgaataagcaccggctaactacgtgccagcagccgcggtaatacgtagggtgcaagcgttatccggaattattg ggcgtaaagggctcgtaggcggttcgtcgcgtccggtgtgaaagtccatcgcttaacggtggatccgcgccgggtacgggcgggcttgagtgcg gtaggggagactggaattcccggtgtaacggtggaatgtgtagatatcgggaagaacaccaatggcgaaggcaggtctctgggccgtcactga cgctgaggagcgaaagcgtggggagcgaaca

>22b156ab488a5a8dfa251815fb0c9b82

tggggaattttccgcaatgggcgaaagcctgacggagcaatgccgcgtggaggtagaaggcccacgggtcatgaacttcttttcccggagaaga agcaatgacggtatctggggaataagcatcggctaactctgtgccagcagccgcggtaatacagaggatgcaagcgttatctggaatgattggg cgtaaagcgtctgtaggtggctttttaagtccgccgtcaaatcccagggctcaactctggacaggcggtggaaactaccaagctggagtacggta ggggcagagggaatttccggtggagcggtgaaatgcgtagagatcggaaagaacaccaacggcgaaagcactctgctgggccgacactgaca ctgagagacgaaagctaggggagcgaatg

>23d531a8631db66781231820eb3d4a56

tggggaatattggacaatgggcgaaagcctgatccagcaatgccgcgtgagtgatgaaggccttagggttgtaaagctcttttacccgggatgat aatgacagtaccgagagaataagccccggctaactccgtgccagcagccgcggtaatacggaggggggctagcgttattcggaattactgggcg taaagcgcacgtaggcggctttgtaagtcagaggtgaaagcctggagctcaactccagaactgcctttgagactgcatcgcttgaatccaggag aggtgagtggaattccgagtgtagaggtgaaattcgtagatattcggaagaacaccagtggcgaaggcggctcactggactggtattgacgctg aggtgcgaaagcgtggggagcaaaca

>23f97de539d47efe31dab47adc172bd6

taaggaatattggtcaatgcccggaagggtgaaccagccatgccgcgtgaaggatgactgccctctgggttgtaaacttcttttggatgggacga aaaaacgggatttatcctggactgacggtaccatcagaataagcaccggctaactccgtgccagcagccgcggtaatacggagggtgcaagcg ttatccggaatcactgggtttaaagggtgcgtaggcggttttataagtcaggggtgaaagcatgcagcttaactgtataattgcctttgatactgta agacttgaataaggttgaggttggcggaatgtgacatgtagcggtgaaatgcttagatatgtcatagaacaccgattgcgaaggcagctgactg ggcctttattgacgctgaggcacgaaagcgtggggagcgaaca

>241693a03d133f908cb6af9fe172911a

tggggaatcttggacaatgggcgcaagcctgatccagccatgccgcgtgagtgaagaaggccttagggttgtaaagctcttttaccagggacga taatgacggtacctggagaataagccccggcaaacttcgtgccagcagccgcggtaatacgaagggggctagcgttgttcggaattactgggcg taaagcgcacgtaggcgggtcgttaagtcaggggtgaaatcccggagctcaactccggaactgcctttgatactggcgatctagagatcgagag aggtgagtggaattccgagtgtagaggtgaaattcgtagatattcggaagaacaccagtggcgaaggcggctcactggctcgatactgacgctg aggtgcgaaagcgtggggagcaaaca

>24224733872ea0131e2751d8b554d73c

tgaggaatattgcgcaatggaggaaactctgacgcagccatgccgcgtgaaggacgactgccctatgggttgtaaacttcttttgtacgggaata
aacccttgctcgtgagcgaggttgaaggtaccgtaagaataagcaccggctaactccgtgccagcagccgcggtaatacggagggtgcaagcg
ttatccggatttattgggtttaaagggtccgtaggcggatcgttaagtcagtggtgaaagcccacagctcaactgtggaactgccattgatactgg
cgatcttgagtgcgtgtgaagttagcggaatgtgtggtgtagcggtgaaatgcttagatatcacacagaacaccaattgcgaaggcagctggct
aacacgcaactgacgctgagggacgaaagtgtggggatcaaaca

>249f01a059913cd078a31dba3d5651ce

tagggaatcttccgcaatggacgaaagtctgacggagcaacgccgcgtgtatgaagaaggttttcggatcgtaaagctctgttgttggtgaaga
aggatagaggtagtaactggcctttatttgacggtaatcaaccagaaagtcacggctaactacgtgccagcagccgcggtaatacgtaggtggc
aagcgttgtccggatttattgggcgtaaagcgagcgcaggcggaaaaataagtctaatgtgaaagccctcggcttaaccgaggaactgcatcgg
aaactgtttttcttgagtgcagaagaggagagtggaactccatgtgtagcggtggaatgcgtagatatatggaagaacaccagtggcgaaggcg
gctctctggtctgcaactgacgctgaggctcgaaagcatgggtagcgaaca

>24a2a019f7af197ef711f8728c88c937

tggggaatcttgcgcaatgggcgaaagcctgacgcagcaacgccgcgtgcgggatgacggccttcgggttgtaaaccgctttcagcagggacg
aaaatgacggtacctgcagaagaagctccggctaactacgtgccagcagccgcggtaatacgtagggagcaagcgttgtccggatttattgggc
gtaaagagctcgtaggcggttccgtaagtcgagtgtgaaaaatctgggctcaacccagtggagcactcgatactgcggtgactagagtccggta
ggggagtgtggaattcctggtgtagcggtgaaatgcgcagatatcaggaggaacaccgacggcgaaggcagcactctgggccggtactgacgc
tgaggagcgaaagcgtggggagcaaaca

>24b4fb451c481ffdc9416946465da973

tgaggaattttccgcaatgggcgaaagcctgacggagcaataccgcgtggaggatgacggcctgtgggtcttaaactccttttcttagagaaga
agttctgacggtatctaaggaataagcatcggctaactccgtgccagcagccgcggtaatacgggggatgcaagcgttatccggaatgattggg
cgtaaagcgtctgtaggtggctttccaagtcaactgtcaaaaatcagggcttaaccttgagccggcagttgaaactatgaagcttgagttaggta
ggggcagagggaattcctagtgtagcggtgaaatgcatagataccataaggaacaccgatagcgaaggcattgtactggccataaactgacgc
tgaggcacgaaagcgtgggtagcgaaca

>24f50b16b37746b08a20a5eb90b4c73a

tgaggaatattgcacaatgggggcaaccctgatgcagcgacgccgcgtggatgatgaaggccttcgggttgtaaagtccttttgccactgacga
gaacggacggtaggtggcgaataagccccggctaactacgtgccagcagccgcggtaaaacgtagggggcgagcgttatccggagttactggg
cgtaaagggtgtgtaggcggtgctataagtggggtgtgaaacgctcaggctaaacctgggaggtgcgcctcagactgtaggactagagtgggat
agaggggcgtggaattccgggtggagcggtgaaatgtgtagagatccggaggaacaccagaggcgcaagcggcgccctgggtcccaactgac
gctgaaacacgaaagcttggggagcgaacg

>252f50a6f92d7a32582ad1a1ea8eee2a

taaggaatattggtcaatggagggaactctgaaccagccatgccgcgtgaaggatgacggccctctgggttgtaaacttcttttggatggggcga
aaaaacgggatttatcctggactgacggtaccatcagaataagcaccggctaactccgtgccagcagccgcggtaatacggagggtgcaagcg
ttatccggaatcactgggtttaaagggtgcgtaggcggttgtataagtcaggggtgaaaggatgcagcttaactgtataattgcctttgatactgt
atgacttgaataaggttgaggttggcggaatgtgacatgtagcggtgaaatgcttagatatgtcatggaacaccgattgcgaaggcagctgact
gggcctttattgacgctgaggcacgaaagcgtgggtagcgaaca

>25584125e3f1ee7c95c4b41e8c9accd8

taaggaatattgggcaatggaggcaactctgacccagccatcccgcgtgaaggattaaggccctatgggttgtaaacttcttttgcttgggaaga
aatctggacatttatgtgcagttgacggtaccttgcgaataagcaccggctaactccgtgccagcagccgcggtaatacggagggtgcaagcgtt
atccggaatcactgggtttaaagggtgcgtaggcggtttgataagtcagaggtgaaagctagcagcttaactgttaaattgcctttgatactgtca

gacttgaatcaggttgaggtaggcggaatgtgacatgtagcggtgaaatgcatagatatgtcatagaacaccaattgcgtaggcagcttgctgg
gcttgaattgacgctgaggcacgaaagcgtggggatcaaaca

>261757c8a6b3be6d75401cc317d6013b

tggggaattttggacaatgggcgcaagcctgatccagccatgccgcgtgagtgaagaaggccttcgggttgtaaagctctttcagccggaaaga
aaacgcacgggataataccctgtgtggatgacggtaccggaagaagaagcaccggctaactacgtgccagcagccgcggtaatacgtagggt
gcgagcgttaatcggaattactgggcgtaaagcgtgcgcaggcggttttgtaagacagatgtgaaatccccgggcttaacctgggaactgcattt
gtaactgcaaggctagagtacggcagagggggggtagaattccacgtgtagcagtgaaatgcgtagatatgtggaggaataccgatggcgaag
gcagccccctgggtcgatactgacgctcatgcacgaaagcgtggggagcaaaca

>27a09ac8f99de7f517bac356cb718ec1

tgaggaatattgcacaatggacgcaagtctgatgcagcaacgccgcgtggaggatgaaggcccttgggtcgtaaactcctttttcggggaaga
gcgaggacggtacccgaagaataagtcacggctaactacgtgccagcagccgcggtaatacgtaggtggcaagcgttatccggaattactggg
cgtaaagcgcacgcaggcggtcttgtaagtttgacgtgaaagctcccggcttaactgggagaggtcgttgaatactgcgagacttgagtttagga
gaggggtgtagaattcccggtgtagtggtggaatgcgtagatatcgggggaataccagtggcgaaagcggcaccctagcctttgactgacgct
catgtgcgaaagcgtggggagcgaacg

>27a8a5e5def204e1d3b37d9cad1b2163

tgaggaatattgggcaatgggcgcaagcctgacccagcaatgccgcgtgcgggatgaaggtcctcggatcgtaaaccgcttttgtgggggagta
gcaaggagagtaccccacgaataagtcacggctaactacgtgccagcagccgcggtaatacgtaggtgacaaatgttatccggaatcactggg
cgtaaagggtctgtaggcggttacttaggtctgatgtgacacctctcggcttaactgagagagcgcattggaaaccgggtgactagaatcacgtc
gaggtcggtggaattcccggtgtagtggtggaatgcgtagagatcgggaggaacctcgaaagcgaaggcagccggctagacgatgattgacgc
tgagggacgatagcgtgggtagcaaaca

>27ca45934f052c3f043c9b8c3e0fbf79

tggggaatcttggacaatgggcgaaagcccgatccagcaatatcgcgtgagtgaagaagggcaatgccgcttgtaaagctctttcgtcgagtgc
gcgatcatgacaggactcgaggaagaagccccggctaactccgtgccagcagccgcggtaagacgggggggggcaagtgttcttcggaatgact
gggcgtaaagggcacgtaggcggtgaatcgggttgaaagtgaaagtcgccaaaaagtggcggaatgctctcgaaaccaattcacttgagtgag
acagaggagagtggaatttcgtgtgtagggggtggaatccgtagatctacgaaggaacgccaaaagcgaaggcagctctctgggtccctaccga
cgctggggtgcgaaagcatggggagcggaca

>2a51faea8805a40017458dfed28abf95

tgaggaatattgcgcaatgggcgaaagcctgacgcagcgacgccgcgtggaggatgacggccttcgggttgtaaactcctgtcagtggggaag
agataggacggtacccactgaggaagcttcggctaactacgtgccagcagccgcggtaagacgtagggagcaagcgttgtccggaattactgg
gcgtaaagagctcgtaggcgggagcgtaagtctgaaaggaaatctcaaggctcaactttggggctttttcggatactgcgtttcttgaggttatca
gaggaaagtagaattcccggtgtagcggtgaaatgcgtagatatcgggaggaataccagtggcgaaggcgactttctgggatacacctgacgc
tgaggagcgaaagcgtggggagcaaacg

>2ae4521786b3fde8d5b03c2d7a969f05

tggggaatattgcacaatgggcgcaagcctgatgcagccatgccgcgtgtgtgaagaaggctctagggttgtaaagcactttcagtagggagga
aagcttgatgattaatactcattaagtgtgacgttacctacagaagaagcaccggctaactccgtgccagcagccgcggtaatacggagggtgc
aagcgttaatcggaattactgggcgtaaagcgcacgtaggtggtttgttaagttggatgtgaaagccccgggctcaacctgggaactgcatcca
aaactggcaaactagagtacggtagaggtgagtggaatttcctgtgtagcggtgaaatgcgtagagatgggaaggaacatcagtggcgaaggc
gactcactggactgatactgacactgaggtgcgaaagcgtgggtagcaaaca

>2bc7b9e573a2b2a6c4532b0201eb5202

tagggaatcttccgcaatggacgaaagtctgacggagcaacgccgcgtgtatgaagaaggttttcggatcgtaaagtactgttgttagagaaga acaaggataagagtaactgcttgtcccttgacggtatctaaccagaaagccacggctaactccgtgccagcagccgcggtaatacggagggtgc gagcgttaatcggaattactgggcgtaaagcgtgcgtaggcggtttcgtaagtcgggtgtgaaagccccgggctcaacctgggaattgcattcga gactgcggagctggagtgcggaagagggaagtggaatttccggtgtagcggtgaaatgcgtagatatcggaaggaacatcagtggcgaaagc gacttcctggtccagcactgacgctcaggcacgaaagcgtggggagcaaaca

>2cddfae297288d5ca73cbbb3d23bd150

tggggaatcttggacaatgggcgaaagcccgatccagcaatatcgcgtgagtgaagaagggcaatgccgcttgtaaagctctttcgtcgagtgc gcgatcatgacaggactcgaggaagaagccccggctaactccgtgccagcagccgcggtaagacggggggggcaagtgttcttcggaatgact gggcgtaaagggcacgtaggcggtgaatcgggttgaaagtgaaagtcgccaaaaagtggcggaatgctctcgaaaccaattcacttgagtgag acagaggagagtggaatttcgtgtgtaggggtgaaatccgta

>2d22082be15e55bfa7cddabbc4f4c48a

tgaggaatattgggcaatgggcggaagcctgacccagcaacgccgcgtggaggatgaaggccttcgggtcgtaaactcctttgacggggaag aggaaggacggtacccgtcgaataagtcacggctaactacgtgccagcagccgcggtaatacgtaggtgacaagcgttatccggaattactgg gcgtaaagcgcacgcaggcggctgcttaagtctgacgtaaaatctcctggcttaactgggaggggtcgttggaaactgagtagcttgaggcagt gagaggggtgtagaattcccggtgtagtggtggaatgcgtagatatcgggaggaataccagaggcgaaggcgacaccctggcactggcctgac gctcatgtgcgaaagcgtggggagcgaacg

>2e1d9bd34c1e9487db5b067664bd02db

tagggaatcttccgcaatggacgaaagtctgacggagcaacgccgcgtgtatgaagaaggttttcggatcgtaaagtactgttgttagagaaga acaaggataagagtaactgcttgtcccttgacggtatctaaccagaaagccacggctaactacgtgccagcagccgcggtaatacggaggggg ttagcgttgttcggaattactgggcgtaaagcgcgcgtaggcggactggaaagttgggggtgaaatcccggggctcaaccccggaactgcctcc aaaactatcagtctagagttcgagagaggtgagtggaattccgagtgtagaggtgaaattcgtagatattcggaggaacaccagtggcgaagg cggctcactggctcgatactgacgctgaggtgcgaaagtgtggggagcaaaca

>2ea64f17e205d8bb1f145b2b4990f5b6

tagggaatattgggcaatggacgagagtctgacccagccatgccgcgtgcaggaagacggcgttctgcgttgtaaactgcttttatctgggaaga aaatgcccctgcgggggaaattgccggtaccagatgaataagcaccggctaactccgtgccagcagccgcggtaatacggagggtgcaagcgt tgtccggatttattgggtttaaagggtgcgtaggcggctgtttaagtcagtggtgaaagactccggctcaaccggagcagtgccattgatactgga cagcttgagtattggaggggtacatggaattgatggtgtagcggtgaaatgcatagataccatcaggaacaccgatagcgaaggcattgtactg gccaataactgacgctgatgcacgaaagcatgggtagcgaaca

>2eff554abc8e8941585317c668a430ab

tccggaatcttggacaattggcgaaagccagatccagccatattacgtgaaggatgaagactttttggttgtaaacttctttactttttaaagtta aagagtataaaaagccccggctaaatctgtgccagcagccgcggtaatacagatggggcaagtgttattcggaatgattgggcgtaaagggta cgtaggtggttttattagtcagttgtcacaaccttttttagaaaagaatcgcagctttaaaagactacaaaacttgagtttgatagaggaaagcgg aattttgagtggagagatagaattcatagatattcaaaggaacaccgaaaacgaaggtagctttctgggtcaacactgacgctcatgtacgaaa gcatggggagtaaatg

>2fda61c8fb70794f30a9770ad9d6bdee

tggggaatattgcacaatgggcgaaagcctgacggagcaataccgcgtggaggatgacggcctgtgggtcttaaactcctttttcttagagaaga agttctgacggtatctaaggaataagcatcggctaactccgtgccagcagccgcggtaatacggggggatgcaagcgttatccggaatgattggg cgtaaagcgtctgtaggtggctttccaagtcaactgtcaaaaatcagggcttaaccttgagccggcagttgaaactatgaagcttgagttaggta ggggcagagggaattcctagtgtagcggtgaaatgcgtagatattaggaagaacaccaatcgcgaaagcactctgctgggccatgactgacac tgagagacgaaagctaggggagcgaatg

>319b1e53bf29bf9d5d4236924ecb2666

tggggaattttccgcaatgggcgaaagcctgacggagcaatgccgcgtggaggtagaaggcccacgggtcatgaacttattttcccggagaag
aagcaatgacggtatctggggaataagcatcggctaactctgtgccagcagccgcggtaatacagaggatgcaagcgttatccggaatgattgg
gcataaagcgtctgtaggtggctttttaagtccgccgtcaaatcctagggctcaactctggacaggcggtggaaactaccaagctggagtacggt
aggggcagagggaatttccggtggagcggtgaaatgcgtagagatcggaaagaacaccaacggcgaacgcactctgctgggccgacactgac
actgagagacgaaagctaggggagcgaatg

>31a3138f02e3ce5e5e20e19f918b59cb

tggggaattttccgcaatgggcgaaagcctgacggagcaatgccgcgtggaggtagaaggcccacgggtcgtgaacttcttttcccggagaaga
agcaatgacggtatctgaggaataagcatcggctaactctgtgccagcagccgcggtaagacagaggatgcaagcgttatctggaatgattgg
gcgtaaggcgtctgtaggtggcttttcaagtccgccgtccaatcccagggctcaaccctggacaggcggtggaaactaccaagctggagtacgg
taggggcagagggaatttccggtggagcggtgaaatgcgtagagatcggaaagtacaccaacggcgaaagcaccctgctgggccgacactga
cactgagagacgaaagctaggggagcgaatg

>31cf8a209b8a034efdcfc64586de530b

tggggaatcttagacaatgggcgcaagcctgatctagccatgccgcgtgagtgatgaaggccttagggtcgtaaagctctttcgccagggatgat
aatgacagtacctggtaaagaaaccccggctaactccgtgccagcagccgcggtaatacggagggggttagcgttgttcggaattactgggcgt
aaagcgcacgtaggcggactattaagtcagaggtgaaatcccggggctcaaccccggaactgcctttgatactggtagtcttgagttcgagaga
ggtgagtggaattccgagtgtagaggtgaaattcgtagatattcggaggaacaccagtggcgaaggcggctcactggctcgatactgacgctga
ggtgcgaaagtgtggggagcaaaca

>31f562dd50fbba77a7c444322458b0ac

tggggaattttccgcaatgggcgaaagcctgacggagcaataccgcgtgagggatgacggcctatgggttgtaaacctctttttcagggagga
acaaaatgacgtgtacctgaagaataagcatcggctaactccgtgccagcagccgcggtaagacggaggatgcaagtgttatccggaatcact
gggcgtaaagcgtctgtaggtggtttaataagtcaactgttaaatcttgaggctcaacctcgaaatcgcagtcgaaactattagactagagtata
gtaggggtaaagggaatttccagtggagcggtgaaatgcgtagagattggaaagaacaccgatggcgaaggcactttactgggctattactaa
cactgagagacgaaagctagggtagcaaatg

>32cc015d582a042949b29ac11915851f

tagggaatattgcacaatgggcgaaagcctgatgcagcaatgccgcgtgtgtgaagaaggcctgagggttgtaaagcactttcaatagtgaag
aaaagcacagggttaataaccttgtgtgttgacgttaactatacaagaagcaccggctaactccgtgccagcagccgcggtaatacggagggtg
caagcgttaatcggaattactgggcgtaaagcgtctgtaggtggctttccaagtcaactgtcaaaaatcagggcttaaccttgagccggcagttg
aaactatgaagcttgagttaggtaggggcagagggaattcctagtgtagcggtgaaatgcgtagatattaggaagaacaccaatcgcgaaagc
actctgctgggccatgactgacactgagagacgaaagctaggggagcgaatg

>332823abd72d40758e55149bceddb7da

tgaggaatattgcccaatgggggaaaccctgaggcagcaacgccgcgtggaggaagaaggtctttacgatcgtaaacttcttttgtgagggaag
aggaaggacggtacctcatgaataagtcacggctaactacgtgccagcagccgcggtaatacgtaggtgacgagcgttatccggaattactggg
cgtaaagcgtacgcaggcggcatggtaagtctgacgtgaaatctcctggcttaactgggagaggtcgttggagactgctgtgcttgagacaatg
agaggggtgcagaattcccggtgtagtggtggaatgcgtagatatcgggaggaataccagaggcgaaagcggcaccctggcattggtctgacg
ctcatgtacgaaagcgtgggtagcaaacg

>3333c920cb06e1e10e2f1e2b0029038a

taaggaatattggacaatggtcggaagactgatccagccatgccgcgtgaaggacgaaggcgctcagcgttgtaaacttcttttgtacgggaac
aaaaccaccaatgcgttggtgtctgagggtaccgtaagaataagcaccggctaactccgtgccagcagccgcggtaatacggagggtgcaagc
gttatccggaatcactgggtttaaagggtgcgtaggcggttcgataagtcagttgtgaaattccctcgcttaacgagtgggactgcgattgatact
gttgaacttgaatcaggttgaggtaggcggaatgtgacatgtagcggtgaaatgcatagatatgtcatagaacaccgattgcgaaggcagcttg
ctggacctgtattgacgctgaggcacgaaagcgtggggagcaaaca

>33b25a87cd5a86c71c5b680d3b9229d3

tggggaattttccgcaatgggcgaaagcctgacggagcaatctcgcgtgagggatgacggcctatgggttgtaaacctctttttcagggaggaa
taaaatgacgtgtacctgaagaataagcatcggctaactccgtgccagcagccgcggtaagacggaggatgcaagtgttatccggaatcactg
ggcgtaaagcgtctgtaggtggtttaataagtcaactgttaaatcttgaagcttaacttcaaaatcgcagttgaaactattagactagagtatagt
aggggtaaagggaatttccagtggagcggtgaaatgcgtagagattggaaagaacaccgatggcgaaggcactttactgggctattactgaca
ctcagagacgaaagctagggtagcaaatg

>3484878bbc7e9747c3e3cc2553721aaf

tggggaattttccgcaatgggcgaaagcctgacggagcaatgccgcgtggaggtggaaggcctacgggtcgtcaacttcttttctcggagaaga
aacaatgacggtatctgaggaataagcatcggctaactctgtgccagcagccgcggtaagacagaggatgcaagcgttatccggaatgattgg
gcgtaaagcgtctgtaggtggcttttcaagtccgccgtcaaatcccagggctcaaccctggacaggcggtggaaactaccaagctggagtacgg
taggggcagagggaatttccggtggagcggtgaaatgcattgagatcggaaagaacaccaacggcgaaagcactctgctgggccgacactga
cactgagagacgaaagctaggggagcaaatgggattagataccccggtag

>3534b4be198dac95882f6e730b647c60

tggggaatattgcacaatgggggaaaccctgatgcagcaacgccgcgtgagtgaagaagtatttcggtatgtaaagctctatcagcagggaag
ataatgacggtacctgactaagaagctccggctaaatacgtgccagcagccgcggtaatacgtatggagcaagcgttatccggatttactgggt
gtaaagggtgcgtaggtggcagtgcaagtcagatgtgaaaggccggggctcaaccccggagctgcatttgaaactgctcggctagagtacagg
agaggcaggcggaattcctagtgtagcggtgaaatgcgtagatattaggaggaacaccagtggcgaaggcggcctgctggactgttactgaca
ctgaggcacgaaagcgtggggagcaaaca

>35684023cba433c1739a36685ae21b57

tgaggaatattgggcaatgggcggaagcctgacccagcaacgccgcgtggaggaagacggccttcgggttgtaaactccttttacgggggaag
aggaaggacggtaccccgagaataagtcacggctaactacgtgccagcagccgcggtaatacgtaggtgacaagcgttatccggaattactgg
gcgtaaagcgcacgcaggcggcgcattaagtctgatgtgaaatctcccggcttaactgggaggggtcattggaaactgatgtgcttgaggcggt
gagaggggtgtagaattcccggtgtagtggtggaatgcgtagatatcgggaggaataccagtggcgaaagcggcaccctggcactagcctgac
gctcatgtgcgaaggcgtggggagcgaacg

>35ab1d83c148531f0c55fed902065d57

tagggaatcttccgcaatggacgaaagtctgacggagcaacgccgcgtgtatgaagaaggttttcggatcgtaaagtactgttgttagagaaga
acaaggataagagtaactgcttgtcccttgacggtatctaaccagaaagccacggctaactacgtgccagcagccgcggtaatacgtaggtggc
aagcgttaatcggaattactgggcgtaaagcgcgcgtaggtggtttgttaagttggatgtgaaagcccagggctcaaccttggaactgcattcaa
aactgactcgctagagtacgagagaggttagtggaatttcctgtgtagcggtgaaatgcgtagagatgggaaggaacaccagtggcgaaggcg
actgactggctcgatactgacactgaggtgcgaaagcgtggggagcaaacg

>362324a4d733ca0d96c9e017cf9c44a7

tgaggaattttccgcaatgggcgaaagcctgacggagcaataccgcgtggaggatgacggcctgtgggtcttaaactccttttcttagagaaga
agttctgacggtatctaaggaataagcatcggctaactccgtgccagcagccgcggtaatacgggggatgcaagcgttatccggaatgattggg
cgtaaagcgtctgtaggtggctttccaagtcaactgtcaaaaatcagggcttaaccttgagccggcagttgaaactatgaagcttgagttaggta
ggggcagagggaattcctagtgtagcggtgaaatgcgtagatattaggaagaacaccaatcgcgaaagcactctgctgggccattggaagaac
accaatcgcgaaagcactctgctgggccatgactgacactgagagacgaaagctaggggagcgaatg

>369d8e9d322e5d94c1d2d45988eb9678

tggggaatcttgcgcaatgggcgaaagcctgacgcagcaacgccgcgtgcgggaagaaggccttcgggttgtaaaccgctttcagaaggaacg
aaactgacggtactttcagaagaaggtgcggccaactacgtgccagcagccgcggtgacacgtaggcaccaagcgttgtccggatttattgggc
gtaaagagctcgtaggcggttcggtaagtcgggtgtgaaaactttgggctcaacccaaagcgtgcactcgatactgccgtgactagagttcggta

ggggagtggggaattcctagtgtagcggtgaaatgcgcagatattaggaggaacaccggtggcgaaggcgccactctgggccgaaactgacgc
tgaggagcgaaagcgtgggtagcaaaca

>36dc31196b3417f0d036e823f1f6043e

tgaggaattttccgcaatgggcgaaagcctgacggagcaatgccgcgtgaaggatgaaggcctatgggttgtaaacttcttttctcagaaaaga
attatgacggtatctgaggaataagcatcggctaactctgtgccagcagccgcggtaagacagaggatgcaagcgttatccggaatgattgggc
gtaaagcgtctgtaggttgtttaaaaagtattctgtcaaagattagggcttaaccctagataggcagaataaacttttaagctagagtttggtaga
ggcagagggaattcccggtggagcggtgaaatgcgtagatatcgggaggaacaccaaaggcgaaggcactctgctgggccaatactaacact
gagagacgaaagcgaggggagcaaatg

>38a5e2958a7ef3691d30bf15ee1d63a2

tggggaatattggacaatgggcgaaagcctgatccagcaataccgcgtgagtgatgacggccttcgggttgtaaagctcttttagtagggaaga
taatgacggtacctacagaaaaagcaccggctaacttcgtgccagcagccgcggtaatacgaagggtgctagcgttgttcggaatcattgggcg
taaagggtgcgtaggcggtttgacaagtcagttgtgaaagcccagggctcaaccctggaactgcaattgaaactgttagactagaatttggtgg
gggatagtagaattcctagtgtaggggtgaaatccgtagatattaggaggaataccagtggcgaaggcgactgtctataccaacattgacgctg
aggcacgaaagcgtggggagcaaaca

>38d57115412b3f39c4f0aeb51bc83e2b

tagggaatattgggcaatggaggcaactctgacccagccatgccgcgtgtaggaagaaggcgttctgcgttgtaaactacttttatcggggaag
aaacgtcatcttgcgaggtgatttgacggtactcgaggaataagcaccggctaactccgtgccagcagccgcggtaatacggagggtgcaagc
gttgtccggatttattgggtttaaagggtgcgtaggtggtttgataagtctgacctgaaagtgggccgcttaacggcacagggtggttggatactg
tcgaacttgaattaggcggaggttgccggaacggatggtgtagcggtgaaatgcatagagatcatccagaacgccaattgcgtaggcaggtgg
ctacgcctatattgacactgaggcacgaaagcatggggagcaaaca

>38dde236a165157c60122f2323c0b023

tagggaatcttccgcaatggacgaaagtctgacggagcaacgccgcgtgtatgaagaaggttttcggatcgtaaagtactgttgttagagaaga
acaaggataagagtaactgcttgtcccttgacggtatctaaccagaaagccacggctaactacgtgccagcagccgcggtaatacgtaggtggc
aagcgttgtccggatttattgggcgtaaagcgagcgcaggcggtcaattaagtctgatgtgaaagcccccggctcaaccggggagggtcattgg
aaactggttgacttgagtgcagaagaggagagtggaattccatgtgtagcggtgaaatgcgtagatatatggaggaacaccagtggcgaaggc
gactctctggtctgtaactgacgctgaggctcgaaagcgtgggtagcaaaca

>3928ddf1976d57ac8d94987814e95410

tggggaatttttcgcaatgggcgaaagcctgacggagcaatgccgcgtggaggtggaaggcctacggttcgtcaacttcttttctcggagaagaa
acaatgacggtatctgagggataagcatcggctaactctgtgccagcagccgcggtaagacagaggatgcaagcgttatccagaatgattggg
cgtaaagcgtctgtaggtggcttttcaagtccgccgtcaaatcccagggctcaaccctggatatgcgacggaaactaccaagctggagtacggta
ggggcagagggaatttccggtggagcggtgaaatgcattgagatcggaaagaacaccaacggtgaaagcactctgcggggtcgacactgaca
ctaagagacgaaagctaggggagcaaatg

>39eb2e97332a21c1039d7078f87fc251

tagggaatattgggcaatggatgcaagtctgacccagccatgccgcgtgcaggaagaaggtcctctggattgtaaactgcttttgtcggggaag
aagagtcatcttgcgaggtgatgtgacggtacccgatgaataagccacggctaactacgtgccagcagccgcggtaatacgtaggtggcaagc
gttgtccggatttattgggtttaaagggtgcgtaggtggtttgctaagtcagtggtgaaagctggttgctcaacaatcaagttgccattgatactgt
cagacttgagagaagtggaggctcatggaatggatggtgtagcggtgaaatgcatagatatcatccagaacgtcgattgcgaaggcagtgggc
tgtactttttctgacactgaggcacgaaagcgtggggatcaaaca

>3a1dd0ac2f3c9915f9d57965195df608

tgaggaatattgcccaatgggcgcaagcctgaggcagcaacgccgcgtggacgatgaaggccttcgggttgtaaagtccttttctgggggaaga
gcaaggacggtaccccaggaaaaagtgtcggctaactacgtgccagcagccgcggtaaaacgtaggacgcgaacgttatccggatttactggg
cgtaaagcgcgtgcaggcggtggcttaagtcgtgcgtgaaagctccctggctcaaccgggagaggccgcgcgaaactgggtcactggaggacg
gtagaggcgtgtggaattccgggtgtagtggtgaaatgcgtagatatccggaggaacaccagtggcgaaggcgacacgctgggccgtacctga
cgctcagacgcgaaagcatgggtagcgaacg

>3a3ad52203346a781c34c4fecbf8ffaf

tggggaattttggacaatgggggaaaccctgatccagccatgccgcgtgcaggatgaaggccttcgggttgtaaactgcttttgtacggaacgaa
aaggtcttttctaataaagaaggctcatgacggtaccgtaagaataagcaccggctaactacgtgccagcagccgcggtaatacgtagggtgca
agcgttaatcggaattactgggcgtaaagcgtgcgcaggcggttatataagacagatgtgaaatccccgggctcaacctgggaactgcattagt
gactgtatagctagagtgcggcagagggggatggaattccgcgtgtagcagtgaaatgcgtagatatgcggaggaacaccgatggcgaaggc
aatcccctgggcctgcactgacgctcatgcacgaaagcgtggggagcaaaca

>3b9e7583526db086712f074037225c90

tggggaatattggacaatgggcgaaagcctgatccagccatgccgcgtgtgtgaagaaggtcttcggattgtaaagcactttaagttgggagga
agggttgtagcttaatacgctgcaaccttgacgttaccaacagaataagcaccggctaactctgtgccagcagccgcggtaatacagagggtgc
aagcgttaatcggaattactgggcgtaaagcgcgcgtaggcggtttgttaagttggatgtgaaagccccgggctcaacctgggaactgcatcca
aaactggcaagctagagtacagtagagggtggtggaatttcctgtgtagcggtgaaatgcgtagatataggaaggaacaccagtggcgaaggc
gaccacctggactgatactgacgctgaggtgcgaaagcgtggggagcaaaca

>3bf736831a6492071c843b2bf9acde07

tggggaatattgcacaatgggcgcaagcctgatgcagccatgccgcgtgtgtgaagaaggccttcgggttgtaaagcactttcagcgaggagga
aaggttagtcgctaatatcggctagctgtgacgttactcgcagaagaagcaccggctaactccgtgccagcagccgcggtaatacggagggtgc
gagcgttaatcggaattactgggcgtaaagcgtgcgtaggcggtttgttaagcgagatgtgaaagcccagggctcaaccttggaactgcatttcg
aactggcaggctagaattttgtagagggtggtggaatttccagtgtagcggtgaaatgcgtagagattggaaggaacatcagtggcgaaggcg
gccacctggacaagaattgacgctgaggcacgaaagcgtgggtagcaaaca

>3cd10ece5b7d9d72123ca1ee0c590466

tggggaattttctgcaatgggcgaaagcctgacagagcaataccgcgtgagggaacgaaggctttcgagttgtaaacctctttttcaaggatga
agatctgacagtacttgaagaataagcatcggctaactctgtgccagcagccgcggtaatacaggggatgcaagtgttatccggaattattggg
cgtaaagcgtctgtaggttgatttataagtcttttgttaaatatttaagcttaacttaaaaaatgcaaaggaaactatttatctagagtttggtaga
ggtaaagggaatttccagtgaaatggtgaaatatttagatattggaaagaacaccaagggcgaaggcattttactgggccataactgacactga
gagacgaaagctaggggagcaaacg

>3cdac49092de5170c435f4b13a995bcd

tagggaatcttcggcaatggacggaagtctgaccgagcaacgccgcgtgagtgaagaaggttttcggatcgtaaagctctgttgtaagagaaga
acgagtgtgagagtggaaagttcacactgtgacggtatcttaccagaaagggacggctaactacgtgccagcagccgcggtaatacgtaggtcc
cgagcgttgtccggatttattgggcgtaaagcgagcgcaggcggttagataagtctgaagttaaaggctgtggcttaaccatagtacgctttgga
aactgtttaacttgagtgcaagaggggagagtggaattccatgtgtagcggtgaaatgcgtagatatatggaggaacaccggtggcgaaagcg
gctctctggcttgtaactgacgctgaggctcgaaagcgtggggagcaaacaggattagataccccggtag

>3d15e3688f6bc1b259179731480ea69d

tggggaatcttagacaatgggggaaaccctgatctagccatgccgcgtggacgatgaaggccttagggttgtaaagtcctttcagctgggaaga
taatgacggtaccagcagaagaagccccggctaactccgtgccagcagccgcggtaatacggagggggctagcgttgttcggaattactgggc
gtaaagcgcacgtaggcggatcagcaagttagaggtgaaatcccggggctcaaccccggaactgcctttaaaactgttggtcttgagttcgaga
gaggtgagtggaattccgagtgtagaggtgaaattcgtagatattcggaggaacaccagtggcgaaggcggctcactggctcgatactgacgct
gaggtgcgaaagcgtggggagcaaaca

>3dd50350bc4b9419a97c68f68c2da444

tggggaatattggacaatgggcgcaagcctgatccagccatgccgcgtgagtgatgaaggccttagggttgtaaagctctttcaccgatgaagat
aatgacggtagtcggagaagaagccccggctaacttcgtgccagcagccgcggtaatacgaagggggctagcgttgttcggaattactgggcgt
aaagcgcacgtaggcggatatttaagtcaggggtgaaatcccggggctcaaccccggaactgcctttgatactgggtatcttgagtatggaaga
ggttagtggaattgcgagtgtagaggtgaaattcgtagatattcgcaggaacaccagtggcgaaggcggctgactggtccattactgacgctga
ggtgcgaaagcgtggggagcaaaca

>3e045903da074348b5e9e8fe6a9f6796

tggggaattttccgcaatgggcgaaagcctgacggagcaatgccgcgtggaggtagaaggcccacgggtcatgaacttcttttcccggagaaga
agcaatgacggtatctgaggaataagcatcggctaactctgtgccagcagccgcggtaatacagaggatgcaagcgttatccggaatgattggg
cgtaaagcgtctgtaggtggctttttaagtccgccgtcaaatcccagggctcaactctggacaggcggtggaaactaccaagctggagtacggta
ggggcagagggaatttccggtggagcggtgaaatgcgtagagatcggaaagaacaccaacggcgaaagcactctgctgggccgacactgaca
ctgagagacgaaagctaggggagcgaatg

>3e6d8538428124e5943fa94e85568e3a

tggggaatcttggacaatgggcgaaagcccgatcaagcaatatcacgtgagtgaagaagggcaatgccgcttgtaaagctcttttgttgagtgc
gtgatcatgatataactcaaggaagaaaccctggctaactccatgctagcagcagcggtaagatgggggcaagtgttctttggaatgactagg
cataaagggcgcgtaggctgtgaatcgggttgaatgtgaaagtcgctaaaaagtggcgaatgctctcgaaaccaattcggttgagtgggaaag
aggagagtggaatttcatgtgtaggggtgaaatccgtagatctatgaaggaacaccagaagcgaaggcagctctatgggtccctaccgatgctg
gagtgagaaagcatggggatcaaaca

>3f3a0eaeea9c0690b6ede1b17b4fd8ce

tggggaatattgcacaatgggcgcaagcctgatgcagcgacgccgcgtgcgggatgacggccttcgggttgtaaaccgcttttgactgggagca
agcccttcggggtgagtgtacctttcgaataagcaccggctaactacgtgccagcagccgcggtaatacgtagggtgcaagcgttatccggaatt
attgggcgtaaagggctcgtaggcggttcgtcgcgtccggtgtgaaagtccatcgcttaacggtggatccgcgccgggtacgggcgggcttgagt
gcggtaggggagactggaattcccggtgtaacggtggaatgtgtagatatcgggaagaacaccaatggcgaaggcaggtctctgggccgtcac
tgacgctgaggagcgaaagcgtggggagcgaaca

>3fe9cac6db71748978f97b08de8e4301

tggggaattttggacaatgggcgaaagcctgatccagccatgccgcgtgagtgaagaaggccttcgggttgtaaagctctttcggtcgggaaga
aatagttgtggctaatatccacaatgaatgacggtaccgacataagaagcaccggctaactacgtgccagcagccgcggtaatacgtagggtgc
aagcgttaatcggaattattgggcgtaaagggtgcgcaggcggttgtataagtcagatgtgaaatccctgggcttaacctaggaattgcgtttga
aactatatgactagagtgtgacagaggggagtggaattccatgtgtagcagtgaaatgcgtagagatgtggaagaacaccgatggcgaaggca
gctccctgggttaacactgacgctcatgcacgaaagcgtggggagcaaaca

>3ffd7a6c44e9d72e8ade2a5627915433

tagggaatattggacaatgggcgaaagcctgatccagcaataccgcgtgagtgatgaaggctattttggtcgtaaagctctttcgtcgaggaag
atgatgacggtactcgaagaagaagccctggctaactccgtgccagcagccgcggtaatacggagagggctagtgttattcggaataattgggc
gtaaagggcacgtaggcggtgaatcaagttgaaagtgaaatcccaaagcttaactttggaactgctttcaaaactgattcactagagtttgatag
gggacagtggaatttctagtgtagagatagaattcataaatattagaaggaacaccagtggcgaaggcgactgtctggatcaaaactgacgctg
aggtgcgaaagcatggggagcaaaca

>400fbcdf8f1ab6e064ee4202e22e07f9

tagggaatattgcgcaatgggcgaaagcctgacgcagcgatgccgcgtgagtgacgaaggtcttaggattgtaaagctcttttctgagggaaga
ataggtctggtaggaaatgatcggattctgacggtacctcaggaataaggaacggctaactccgtgccagcagccgcggtaatacggaggttcc
aagcgttgttcggaattactgggcgtaaagggagcgcaggcggacaggtcagtttaaagtggaatcccagggctcaaccctggacgtgctttgg

atactgcctgtcttgagttcgtgagaggaaagtggaattcagggtgtagcggtgaaatgcttagatatcctgaggaacaccagtggcgaaagcg
actttctggcacgaaactgacgctgaggctcgaaagcatgggtagcgaacg

>40449520ee5239c0dc0d2f4125df7dce

tggggaatattggacaatgggcgcaagcctgatccagccatgccgcgtgagtgatgaaggccctagggttgtaaagctcttttgtgcgggaaga
taatgacggtaccgcaagaataagccccggctaacttcgtgccagcagccgcggtaatacgaagggggctagcgttgctcggaatcactgggc
gtaaagcgcacgtaggcgggttcttaagtcagaggtgaaatcctggagctcaactccagaactgcctttgatactgggagtctagagttcgggag
aggtgagtggaactgcgagtgtagaggtgaaattcgtagatattcgcaagaacaccagtggcgaaggcggctcactggcccgatactgacgct
gaggtgcgaaagcgtggggagcaaaca

>4046d107e680a0fa8a0e45b91f169b1c

tagggaattgttcgcaatgggcgcaagcctgacgacgcaacgccgcgtggaggatgaagggcttcggctcgtaaactcctttcgaccgagatga
atacccgcagacttaatacgtttgcggactgacagtatcgagggaagaagccccggctaactccgtgccagcagccgcggtaatacggggggg
gcaagcgttgttcggaattactgggcgtaaagggttcgtaggtggccaactaagtcatacgtgaaatccctcagcttaactggggaactgcgtct
gatactggttggcttgagtgcaggagaggaacgcggaattccaggtgtagcggtgaaatgcgtagatatctggaggaacaccggtggcgaagg
cggcgttctggactgtaactgacactgaggaacgaaagccaggggagcaaacg

>4098e79fc7e429d240cb80dfbe5180e2

tgaggaatattgggcaatggaggcaactctgacccagccatgccgcgtgcaggaagacggccctatgggttgtaaactgcttttatacaggaag
aaacactgctacgtgtagcagcttgacggtactgtaagaataagcaccggctaactccgtgccagcagccgcggtaatacgggggatgcaagc
gttatccggaatgattgggcgtaaagcgtctgtaggtggctttttaagttctccgtcaaatcccagggctcaaccctggacaggcggtgaaaacta
ccaagctagagtacggtaggggcagagggaatttccggtggagcgatgaaatgcgtagagatcggaaggaacaccaacggcgaaagcactct
gctgggccgacactgacactgagagacgaaagctaggggagcaaatg

>409f711b59152d57926cf444c5577087

tagggaatcttcggcaatgggggcaaccctgaccgagcaacgccgcgtgagtgaagaaggttttcggatcgtaaagctctgttgtaagtcaaga
acgagtgtgagagtggaaagttcacactgtgacggtagcttaccagaaagggacggctaactacgtgccagcagccgcggtaatacgtaggtc
ccgagcgttgtccggatttattgggcgtaaagcgagcgcaggcggtttgataagtctgaagttaaaggctgtggctcaaccatagttcgctttgga
aactgtcaaacttgagtgcagaaggggagagtggaattccatgtgtagcggtgaaatgcgtagatatatggaggaacaccggtggcgaaagcg
gctctctggtctgtaactgacgctgaggctcgaaagcgtggggagcgaaca

>40e8b3bc4f482bc7c62eed3a5d340af1

tagggaatcttccgcaatggacgaaagtctgacggagcaacgccgcgtgtatgaagaaggccttcgggttgtaaagcactttcagtaaggagg
aaaggttaagtgttaatagcgcttagctgtgacgttacttacagaagaagcaccggctaactccgtgccagcagccgcggtaatacggagggtg
cgagcgttaatcggaattactgggcgtaaagcgtacgcaggcggtttgttaagcgagatgtgaaagccccgggctcaacctgggaactgcattt
cgaactggcaaactagagtgtgatagagggtggtagaatttcaggtgtagcggtgaaatgcgtagagatctgaaggaataccgatggcgaagg
cagccacctgggtcaacactgacgctcatgtacgaaagcgtggggagcaaaca

>4169a76114b0f1a068b71ee3adf7befe

tgaggaattttccgcaatgggcgaaagcctgacggagcaatgccgcgtgaaggaagaaggctcacgggtcgtaaacttcttttctcggagaaga
ataaatgacggtatctgaggaataagcatcggctaactctgtgccagcagccgcggtaaaacagaggatgcaagcgttatccggaattattggg
cgtaaagtgtctgtaggtggcttttcaagtccgtcgtcaaatcccagggctcaaccctggacaggcggtggaaactatcaagctagagtacggta
gaggcagagggaatttccggtggagcggtgaaatgcgttgagatcgggaggaacaccaagggcgaaagcactctgctgggccgttactgaca
ctcagagacgaaagctaggggagcaaatg

>42134c0ca555e7f9188352bddbb271da

ttaggaatattcgtcaatgggggaaaccctgaacgagcaatgccgcgtgaaggaagacggtcctctggattgtaaacttctgttgttagggaag
aacgacctgtgtaggaaatgacataggagtgacggtacctttcaagaaagctccggctaactacgtgccagcagccgcggtaatacgtaggga
gcgagcgttatccggaattattgggtgtaaagcgtgtgtaggcgggaaattaagtctaaggtctaagcccggagctcaactccggttcgccttag
aaactgattttcttgagtgtggtagaggcaaacggaatttctagtgtagcggtaaaatgcgtagatattagaaggaacaccagtggcgaaggcg
gtttgctgggccactactgacgctgagacacgaaagcgtggggagcaaata

>422a89f4b23ffe659b4707d193059776

tagggaatattggacaatggacgaaagtctgatccagcaacgccgcgtgaaggatgaaggtcttaggattgtaaacttcttttgacagggagga
taatgactgtacctgtagaataagcaccggctaacttcgtgccagcagccgcggtaatacgaagggtgccagcattactcggaatcactgggcg
taaagaatgcgtaggcggacttgcaagttaggagtgaaatccctgggctcaacctaggaactgcttttaaaactgcttgtcttgaatgctagagt
gattaggggaattcttggtgtaggggtaaaatccgtagatatcaggaggaagaccggaagcgaaagcgcttaattagctagatattgacgctga
tgcatgaaagcgtggggagcaaaca

>4233f16ea5b9a5801fdb1ed10e01c3bd

caaggaattttcggcaatgggcgcaagcctgaccgagcaacgccgcgtggaggacgacggccttcgggttgtaaactccttttgcgggggacga
ttgtgactgtaccccgcgaatcaggctcggctaactctgtgccagcagccgcggtaagacagaggagccaagcgttgtccggattgactgggcg
taaagcgcacgcaggcggctgggcacgtggggtgtgaaacctggcggcttaactgccagacgccatcccatacggcccggctcgaggcaggca
gaggttcgtggaattgccggtgtagtggtgaaatgcgtagagatcggcaggaacaccaagggcgaaagcagcgaactgggcctgttctgacgc
tcaggtgcgaaagcgtggggagcgaacc

>427a6228ffeff322ed0884d62b458799

tggggaattttggacaatggacgcaagtctgatccagcaatgccgcgtgcaggacgaaggccttcgggttgtaaactgcttttgtacagaacga
aaaagtctttaataacactagaggctcatgacggtactgtaagaataagcaccggctaactacgtgccagcagccgcggtaatacgtagggtgc
aagcgttaatcggaattactgggcgtaaagcgtgcgcaggcggttatataagacagatgtgaaatccccgggctcaacctgggacctgcatttgt
gactgtatagctagagtacggtagaggggatggaattccgcgtgtagcagtgaaatgcgtagatatgcggaggaacaccgatggcgaaggca
atcccctggacctgtactgacgctcatgcacgaaagcgtggggagcaaaca

>427f5142ae52662b7db0bdd5689461f0

tagggaatcttcggcaatggacgaaagtctgaccgagcaacgccgcgtgagtgaagaaggttttcggatcgtaaaactctgttgttagagaaga
acaaggatgagagtaactgttcatcccttgacggtatctaaccagaaagccacggctaactacgtgccagcagccgcggtaatacgtaggtggc
aagcgttgtccggatttattgggcgtaaagcgagcgcaggcggtttcttaagtctgatgtgaaagcccccggctcaaccggggagggtcattgga
aactgggagacttgagtgcagaagaggagagtggaattccatgtgtagcggtgaaatgcgtagatatatggaggaacaccagtggcgaaggc
ggctctctggtctgtaactgacgctgaggctcgaaagcgtggggagcaaaca

>42a83897f7eb0e989498521c8d97ef70

tagggaatcttccgcaatggacgaaagtctgacggagcaacgccgcgtgtatgaagaaggttttcggatcgtaaagtactgttgttagagaaga
acaaggataagagtaactgcttgtcccttgacggtatctaaccagaaagccacggctaactccgtgccagcagccgcggtaatacggagggtgc
aagcgttaatcggaattactgggcgtaaagcgcgcgtaggcggcttagtaagtcggatgtgaaatcccagggcttaaccttggaactgcattcg
atactgctaggctagagtatgttagaggcaagtggaattccaggtgtagcggtgaaatgcgtagatatctggaggaacaccagtggcgaaggc
gacttgctgggacaatactgacgctgaggtgcgaaagcgtggggagcaaaca

>42d354614bd2e8209fddbd2047d531bf

tggggaatattgcacaatggagggaactctgatgcagcgacgccgcgtgagcgatgaaggctttcgagtcgtaaagctctgtcgaatgggaag
ataatgacggtaccatttgagaaagccccggctaactacgtgccagcagccgcggtaatacgtagggggcgagcgttgtccggaattattgggc
gtaaagggttcgtaggcggcaatgtatgtcaggtgtgaaagacggtggctcaaccaacgcaagcatctgaaactgcatagcttgagaaaagga
gaggtaagtggaattcctagtgtagcggtgaaatgcgtagatattaggaggaataccagtggcgaaggcgacttactggactttaactgacgct
gatgaacgaaagcgtggggagcaaaca

>4394468b49611f78b96b64d72449fab5

tagggaatcttccacaatgggcgcaagcctgatggagcaacaccgcgtgagtgaagaagggtttcggctcgtaaagctctgttgttaaagaaga
acacgtatgagagtaactgttcatacgttgacggtatttaaccagaaagtcacggctaactacgtgccagcagccgcggtaatacgtaggtggc
aagcgttatccggatttattgggcgtaaagagagtgcaggcggttttctaagtctgatgtgaaagccttcggcttaaccggagaagtgcatcgga
aactggataacttgagtgcagaagagggtagtggaactccatgtgtagcggtggaatgcgtagatatatggaagaacaccagtggcgaaggcg
gctacctggtctgcaactgacgctgagactcgaaagcatgggtagcgaaca

>43a54829cc0d7a58a61ad7f833cc2b22

tagggaatcttccgcaatgggcgaaagcctgacggagcaacgccgcgtgagtgaagaaggtcttcggatcgtaaaactctgttattagggaag
aacaaatgtgtaagtaactatgcacgtcttgacggtacctaatcagaaagccacggctaactacgtgccagcagccgcggtaatacgtaggtgg
caagcgttatccggaattattgggcgtaaagcgcgcgtaggcggtttttttaagtctgatgtgaaagcccacggctcaaccgtggagggtcattgg
aaactggaaaacttgagtgcagaagaggaaagtggaattccatgtgtagcggtgaaatgcgcagagatatggaggaacaccagtggcgaagg
cgactttctggtctgtaactgacgctgatgtgcgaaagcgtggggatcaaaca

>43aaec1829316c299caafecc47e33d65

tggggaatattgcacaatgggcgcaagcctgatgcagcgacgccgcgtgagggatgacggccttcgggttgtaaacctctttcagcagggaaga
agcgaaagtgacggtacctgcagaagaagcaccggctaactacgtgccagcagccgcggtaatacgtagggtgcgagcgttgtccggaattat
tgggcgtaaagagcttgtaggcggtttgtcgcgtctgcagtgaaaatccggggctcaaccccggacttgctgtgggtacgggcagactagagtgt
ggtaggggagaccggaattcctggtgtagcggtggaatgcgcagatatcaggaggaacaccgatggcgaaggcaggtctctgggccacaactg
acgctgagaagcgaaagcatggggagcgaaca

>43c634736bf9a70837bf33cc15d15143

tggggaatattgcacaatgggggaaaccctgatgcagccatgccgcgtgtgtgaagaaggccttcgggttgtaaagcactttcagcgaggagga
aaggttgatacctaatacgtatcagctgtgacgttactcgcagaagaagcaccggctaactccgtgccagcagccgcggtaatacggagggtgc
aagcgttaatcggaattactgggcgtaaagcgcgcgtaggtggtttgttaagtggaatgtgaaagccctgggctcaacctaggaactgcatccc
aaactgacagactagagtacaatagaggggtgtggaatttcctgtgtagcggtgaaatgcgtagatataggaaggaacaccagtggcgaaggc
gacaccctggattgatactgacactgaggtgcgaaagcgtggggagcaaaca

>43cdcdfdf1af40850bdbd8b6ef9f263c

tagggaatcttccgcaatggacgaaagtctgacggagcaacgccgcgtgtatgaagaaggccttagggttgtaaacccctttcagcggggaag
ataatgacggtacccgcagaagaagccccggctaactccgtgccagcagccgcggtaatacggagggggctagcgttgttcggaattactggg
cgtaaagcgcgcgtaggcggatctgcaagtcaggggtgaaatcccgaggctcaacctcggaactgcctttgatactgcggatcttgagtccgag
agaggtgagtggaattcctagtgtagaggtgaaattcgtagatattaggaagaacaccagtggcgaaggcggctcactggctcggtactgacgc
tgaggcgcgaaagcgtggggagcaaaca

>4424abb9b48a8cbe94a9f770ccdb5b72

tggggaatattgcgcaatgggcgaaagcctgacgcagcaacgccgcgtgcgggatgaaggccttcgggttgtaaaccgctttcagtggggacg
aattcagacggtacccacagaagaagccccggccaactacgtgccagcagccgcggtaacacgtagggggcgagcgttgtccggatttattgg
gcgtaaagagctcgtaggcggctaagtaagtgaggtgtgaaaactccaggctcaacctggagacgccacctcatactgctttggctagagtccg
gtaggggagcatggaattcctggtgtagcggtgaaatgcgcagatatcaggaggaacaccagtggcgaaggcggtgctctgggccggtactga
cgctgaggagcgaaagcgtggggagcaaaca

>44f1735bd70d89609e5c57d046d2082a

tggggaatattgcacaatgggcggaagcctgatgcagcaacgccgcgtgcgggatgacggccttcgggttgtaaaccgctttcgcctgtgacga
agcgtgagtgacggtaatgggtaaagaagcaccggctaactacgtgccagcagccgcggtgatacgtagggtgcgagcgttgtccggatttatt
gggcgtaaagggctcgtaggtggttgatcgcgtcggaagtgtaatcttggggcttaaccctgagcgtgctttcgatacgggttgacttgaggaag

gtaggggagaatggaattcctggtggagcggtggaatgcgcagatatcaggaggaacaccagtggcgaaggcggttctctgggcctttcctgac
gctgaggagcgaaagcgtggggagcgaacaggattagataccccagtag

>44f1dc6802761224c2a2cd9047d09294

tggggaatattgcacaatgggcgaaagcctgatgcagcaacgccgcgtgagggatgacggccttcgggttgtaaacctcttttagtagggaaga
agcgaaagtgacggtacctgcagaaaaagcaccggctaactacgtgccagcagccgcggtaatacgtagggtgcaagcgttgtccggaattat
tgggcgtaaagagctcgtaggcggtttgtcgcgtctgctgtgaaatcccgaggctcaacctcgggtctgcagtgggtacgggcagactagagtgt
ggtaggggagattggaattcctggtgtagcggtggaatgcgcagatatcaggaggaacaccgatggcgaaggcagatctctgggccattactg
acgctgaggagcgaaagcatggggagcgaaca

>4504469c97ce9a8eb989bc0bad2bf4ba

tagggaatcttccgcaatggacgaaagtctgacggagcaacgccgcgtgtatgaagaaggttttcggatcgtaaagtactgttgttagagaaga
acaaggataagagtaactgcttgtcccttgacggtatctaaccagaaagccacggctaactacgtgccagcagccgcggtaatacggagggtgc
aagcgttatccggaatcactgggtttaaagggtgcgtaggcggcctggtaagtcagaggtgaaatgcacgagcttaactggtgaattgcctttga
aactatcaagctagaattatgttgaggtcagcggaatgagtcatgtagcggtgaaatgcatagatatgacttagaacaccgattgcgtaggcag
ctggctaggcatatattgacgctgaggcacgaaagcgtggggagcgaaca

>4614cd89c6385e270c69f3b383f5d5ce

tgaggaattttccgcaatgggcgaaagcctgacggagcaatgccgcgtggaggtagaaggcccacgggtcgtgaacttcttttcccggagaaga
agcaatgacggtatctggggaataagcatcggctaactctgtgccagcagccgcggtaatacagaggatgcaagcgttatccggaatgattggg
cgtaaagcgtctgtaggtggctttttaagtccgccgtcaaatcccagggctcaaccctggacaggcggtggaaactaccaagctggagtacggt
aggggcagagggaatttccggtggagcggtgaaatgcgtagagatcggaaagaacaccaacggcgaaagcactctgctgggccgacactgac
actgagagacgaaagctaggggagcgaatg

>46cb0161377ebc0ca0fb7a9b5ac56164

tcgagaatcattcacaatgggcgaaagcctgatggtgtgacgccgcgtggaggatgaaggtcttcggattgtaaactcctgtcaagtgagagta
aagccgggcgttaactgcgagccggtttgagagtatcactagaggaagagacggctaactctgtgccagcagccgcggtaatacagaggtctc
aagcgttgttcggaatcactgggcgtaaagggtgcgtaggctgcacggtaagtcagatgtgaaagcccggagctcaacttcggaattgcatccg
atactgccgtgcttgaggactggagaggagattggaattcacagtggagcagtgaaatgcgtagatattgtgaggaacactagtggcgaaggc
gaatctctggacagttcctgacgctgaggcacgaaggccaggggagcaaacg

>47082eb92759c2179725fe69f11672c5

tggggaattttggacaatgggcgaaagcctgatccagccatgccgcgtgcaggatgaaggccttcgggttgtaaactgcttttgtacggaacgaa
acggccttttctaataaagagggctaatgacggtaccgtaagaataagcaccggctaactacgtgccagcagccgcggtaatacgtagggtgca
agcgttaatcggaattactgggcgtaaagcgtgcgcaggcggttatgtaagacagttgtgaaatccccgggctcaacctgggaactgcatctgtg
actgcatagctagagtacggtagagggggatggaattccgcgtgtagcagtgaaatgcgtagatatgcggaggaacaccgatggcgaaggca
atcccctggacctgtactgacgctcatgcacgaaagcgtggggagcaaaca

>483c89bebaf35922d7ed4d83f2471304

tgaggaattttctgcaatgggcgaaagcctgacagagcaatatcgcgtgagggaggaaggcctacgggttgtaaacctcttttctcaaggagga
aactatgacgttacttgaggaataagcatcggctaaccctgtgccagcagccgcggtaatacaggggatgcaagtgttatccggaatcattggg
cgtaaagcgtctgcaggctggcttttaagtcttttgttaaagctctaggctcaacctagaaaatgcaaaggaaactaaaagccttgagtatggta
gaggtaaagggaatttccagtggagcagtgaaatgcttagatattggaaagaacaccaacggcgaaggcactttactgggccagcactgacgc
tgagagacgaaagctaggggagcaaacg

>48574cce00abc6753c1e7b230012cfdc

tggggaatattggacaatgggggcaaccctgatccagcgacgccgcgtgtgtgaagaaggcctgcgggttgtaaagcacttttagtggggagg
aaaagctcagacctaacacgtctgggaattgacttaacccaaagaaaaagcaccggctaactctgtgccagcagccgcggtaatacagagggt
gcgagcgttaatcggaattactgggcgtaaagcgtgcgtagacggttatctaagtcgggtgtgaaatccccgggctcaacctgggaattgcattc
gagactgaatagctagggttcggaagagggaagcggaatttccggtgtagcggtgaaatgcgtagatatcggaaggaacatcagtggcgaaa
gcggcttcctggtccagaaccgacgttcaggcacgaaagcgtggggagcaaaca

>492e2cc5c85859e2f98ec56452284e51

tggggaatattggacaatgggcggaagcctgatccagcaacgccgcgtgagggatgacggccttcgggttgtaaacctctttcagtaccgacga
agcgcaagtgacggtaggtacagaagaagcaccggccaactacgtgccagcagccgcggtaatacgtagggtgcgagcgttgtccggaattat
tgggcgtaaagggctcgtaggcggtttgtcgcgtcgggagtgaaaaccaggtgcttaacacctggcttgctttcgatacgggcagactagaggta
ttcaggggagaacggaattcctggtgtagcggtgaaatgcgcagatatcaggaggaacaccggtggcgaaggcggttctctgggaatatcctg
acgctgaggagcgaaagtgtggggagcgaaca

>497c2e1d6c8d903650af16a108d373a1

tggggaattttccgcaatgggcgaaagcctgacggagcgacgctgcgtgaaggatgacggcctgcgggttgtaaacttcttttctcgaagaaga
ataaatgacggtattcgaggaataagcatcggctaactctgtgccagcagccgcggtaagacagaggatgcaagtgttattcggattgattggg
cgtaaagcgtctgtaggcggtttggaaagtcttttgtgaaattcttcagctcaactgggggtccgcaaaagaaacttctaggcttgagggaagta
gaggtacagggaatttccggtggagcggtgaaatgcgtagatatcggaaggaacaccaatatggcgaaggcactgtactgggctttacctgac
gctaagagacgaaagctaaaggagtgatta

>4a7a4813b0e0cac8daeb99b59edd44a6

tggggaatattggacaatgggcgaaagcctgatccagcaatgccgcgtgagtgatgaaggccttagggttgtaaagctcttttacccgggatgat
aatgacagtaccgggagaataagctccggctaactccgtgccagcagccgcggtaatacggagggagctagcgttgttcggaattactgggcgt
aaagcgcacgtaggcggctttgtaagttagaggtgaaagcctggagctcaactccagaattgcctttaagactgcatcgctcgaatccaggaga
ggtgagtggaattccgagtgtagaggtgaaattcgtagatattcggaagaacaccagtggcgaaggcggctcactggactggtattgacgctga
ggtgcgaaagcgtggggagcaaaca

>4a7f867d55034fd3b2b4b28e0b1dda36

tggggaatattgcacaatgggcgaaagcctgatgcagcgacgccgcgtgagggatgacggccttcgggttgtaaacctctttcagcagggacga
agcgcaagtgacggtacctgcagaagaagcaccggctaactacgtgccagcagccgcggtaatacgtagggtgcaagcgttgtccggaattac
tgggcgtaaagagttcgtaggcggtttgtcgcgtcgtttgtgaaaaccagcagctcaactgctggcttgcaggcgatacgggcagacttgagtac
tgcaggggagactggaattcctggtgtagcggtgaaatgcgcagatatcaggaggaacaccggtggcgaaggcgggtctctgggcagtaactg
acgctgaggaacgaaagcgtgggtagcgaaca

>4a8f14a41015a1bf6ee196eb3affbd56

tggggaatattgcgcaatgggcgaaagcctgacgcagccacgccgcgtgagcgatgaaggccttcgggtcgtaaagctctgtggggagagacg
aagaaagccggtgaagagccggccttgacggtatctccttagcaagcaccggctaactccgtgccagcagccgcggtaatacggagggtgcaa
acgttgctcggaatcattgggcgtaaagcgcacgtaggcggcgaagcaagttgggtgtgaaagccctgggctcaacccaggaagtgcattcaa
aactgcttcgcttgaatctcggagggggtcagagaattcccggtgtagaggtgaaattcgtagatatcgggaggaataccagtggcgaaggcgc
tgacctggacgaagattgacgctgaggtgcgaaagcgcggggagcaaaca

>4ac2359a61bc31e062026924341c6464

tggggaattttggacaatgggcgaaagcctgatccagcaatgccgcgtgtgtgaagaaggccttcgggttgtaaagcacttttgtccggaaaga
aatcctttgggttaatacctcggggggatgacggtaccggaagaataagcaccggctaactacgtgccagcagccgcggtaatacgtagggtgc
aagcgttaatcggaattactgggcgtaaagcgtgcgcaggcggttttgtaagacggatgtgaaatccccgggcttaacctgggaactgcattcgt
gactgcaaggctagagtatggcagaggggggtagaattccacgtgtagcagtgaaatgcgtagagatgtggaggaataccgatggcgaaggc
agccccctgggccaatactgacgctcatgcacgaaagcgtggggagcaaaca

>4ad3ff35fcf6cc001d50d5e4a0cf1572

tggggaatattgcacaatgggcgcaagcctgatgcagcaacgccgcgtgagggatgacggccttcgggttgtaaacctcttttagtaaggaaga
agccgcaaggtgacggtacttgcagaaaaagcaccggctaactacgtgccagcagccgcggtaatacgtagggtgcaagcgttgtccggaatt
attgggcgtaaagagctcgtaggcggtttgtcgcgtctgccgtgaaagcccgaggctcaacctcgggtctgcggtgggtacgggcaggctagag
tgcggtaggggagattggaattcctggtgtagcggtggaatgcgcagatatcaggaggaacaccgatggcgaaggcagatctctgggccgtta
ctgacgctgaggagcgaaagcatggggagcgaaca

>4d93383d0d775f7979f2fbe804ff9997

tggggaattttggacaatgggcgaaagcctgatccagccatgccgcgtgcaggatgaaggccttcgggttgtaaactgcttttgtacggaacgaa
aagactctggttaatacctggggtccatgacggtaccgtaagaataagcaccggctaactacgtgccagcagccgcggtaatacgtagggtgca
agcgttaatcggaattactgggcgtaaagcgtgcgcaggcggttatataagacagatgtgaaatccccgggctcaacctgggaactgcatttgt
gactgtatagctagagtacggcagaggggggatggaattccgcgtgtagcagtgaaatgcgtagatatgcggaggaacaccgatggcgaaggc
aatcccctgggcctgtactgacgctcatgcacgaaagcgtggggagcaaaca

>4e38cc2c28e798a73bd2e6a218c04b37

tggggaatattggacaatgggcgcaagcctgatccagccatgccgcgtgagtgatgacggccttagggttgtaaagctcttttacccgggaagat
aatgacggtaccgggagaataagccccggctaacttcgtgccagcagccgcggtaatacgaaggggggctagcgttgttcggaatcactgggcgt
aaagcgcacgtaggcggatctttaagtcaggggtgaaatcccggggctcaaccccggaactgcctttgatactggggatctcgagttcgggaga
ggtgagtggaactgcgagtgtagaggtgaaattcgtagatattcgcaagaacaccagtggcgaaggcggctcactggcccgatactgacgctg
aggtgcgaaagcgtggggagcaaaca

>4e40b0ab511cadfdacfa1caa47c851d5

tgaggaattttccgcaatgggcgaaagcctgacggagcaataccgcgtggaggatgacggcctgtgggtcttaaactccttttcttagagaaga
agttctgacggtatctaaggaataagcatcggctaactccgtgccagcagccgcggtaatacgggggatgcaagcgttatccggaatgattggg
cgtaaagcgtctgtaggtggctttccaagtcaactgtcaaaaatcagggcttaaccttgagccggcagttgaaactatgaagcttgagttaggta
ggggcagagggaattcctagtgtagcggtgaaatgcgtagatattaggaagaacaccaatcgcgaaagcactctgctgggccatgactgacac
tgagagacgaaagctaggggagcgaatg

>4eb7217a641476257c60a1b361649930

tgaggaattttccgcaatgggcgaaagcctgacggagcaataccgcgtgaaggattaaggcctatgggttgtaaacttcttttctcagagaagaa
ttttgacggtatctgaggaataagcatcggctaactctgtgccagcagccgcggtaagacagaggatgcaagcgttatccggattaattgggcgt
aaagcgtctgtaggtggtttatcaagtttttttgtcaaagatcggggcttaactccgggcaggcagagaaaactagtagacttgagttcggtaggg
gcagagggaattcccggtggagcggtgaaatgcgtagagatcgggaggaacaccaaaggcgaaggcactctgctgggccgcaactgacactg
agagacgaaagcgagggtagcaaatg

>4f11f4b7bc5241fa2c1a694d41089e41

tggggaatattgcacaatgggcgcaagcctgatgcagccatgccgcgtgtgtgaagaaggccttcgggttgtaaagcactttcagtcgtgagga
aggtggtagtgttaatagcactatcatttgacgttagcgacagaagaagcaccggctaactccgtgccagcagccgcggtaatacggagggtgc
gagcgttaatcggaattactgggcgtaaagcgcacgcaggcggtttgttaagctagatgtgaaagccccgagctcaacttgggatggtcatttag
aactggcagactagagtcttggagaggggagtggaattccaggtgtagcggtgaaatgcgtagatatctggaggaacatcagtggcgaaggcg
actccctggccaaagactgacgctcatgtgcgaaagtgtgggtagcgaaca

>4f693533099e27dc7b6ed4f822145f4e

tgaggaatattgggcaatggaggcaactctgacccagccataccgcgtgaaggaagactgtcctatggattgtaaacttcttttatatgggaaga
ataaggtctacgtgtagactgatgactgtaccatatgaataaggaccggctaactccgtgccagcagccgcggtaatacggagggttcaagcgt
tatccggatttattgggtttaaagggtccgtaggcggactaataagtcagtggtgaaatcctatagctcaactatagaactgccattgaaactgtt

agtcttgaattatttggaagttggcggaatgagtagtgtagcggtgaaatgcatagatattactcagaacaccgattgcgaaggcagctgactac
gaatatattgacgctgatggacgaaagcgtaggtagcgaaca

>51dc7031d4636e88142b59059317de39

tggggaatattggacaatgggcgcaagcctgatccagccatgccgcgtgagtgatgaaggccttagggttgtaaagctctttcagtagggaaga
taatgacggtacctacagaagaagccccggctaacttcgtgccagcagccgcggtaatacgaagggggctagcgttgttcggatttactgggcg
taaagcgcacgtaggcggatcgttaagtcaggggtgaaatcctggagctcaactccagaactgcctttgatactggcgatctcgagtccggaag
aggtgagtggaactcctagtgtagaggtggaattcgtagatattaggaagaacaccagtggcgaaggcggctcactggtccggaactgacgct
gaggtgcgaaagcgtggggagcaaaca

>5275d0e6bbcef9e6101af982907cf88a

tagggaatcttccgcaatgggcgaaagcctgacggagcaacgccgcgtgagtgattaaggtcttcggatcgtaaaactctgttattagggaaga
acaaatgtgtaagtaactatgcacgtcttgacggtacctaatcagaaagccacggctaactacgtgccagcagccgcggtaatacgtaggtggc
aagcgttatccggaattattgggcgtaaagcgcgcgtaggcggtttttttaagtctgatgtgaaagcccacggctcaaccgtggagggtcattgga
aactggaaaacttgagtgcagaagaggaaagtggaattccatgtgtagcggtgaaatgcgcagagatatggaggaacaccagtggcgaaggc
gactttctggtctgtaactgacgctgatgtgcgaaagcgtggggatcaaaca

>528a228f1a944feaa9c9cddfdf2f57b6

tagggaatcttccgcaatggacgaaagtctgacggagcaacgccgcgtgtatgaagaaggttttcggatcgtaaagtactgttgttagagaaga
acaaggataagagtaactgcttgtcccttgacggtatctaaccagaaagccacggctaactacgtgccagcagccgcggtaatacgtaggtggc
aagcgttgtccggatttattgggcgtaaagcgcgcgcaggcggtcttttaagtctgatgtgaaagcccccggcttaaccggggagggtcattgga
aactggaagactggagtgcagaagaggagagtggaattccacgtgtagcggtgaaatgcgtagatatgtggaggaacaccagtggcgaaggc
gactctctggtctgtaactgacgctgaggcg

>53b7dfa9f189c44ffda0eef1a1c9f53d

tgaggaattttctgcaatgggcgaaagcctgacagagcaataccgcgtgggggatgaagacttactgagttgtaaacctcggtaccctaaggaa
gaagatatgacggtacttagggtggaaagcatcggctaactccgtgccagcagccgcggtaagacgggggatgcaagtgttatccggatttact
gggcgtaaagcgtctgcaggtggtttcttaagtctactgttaaatcttgaggctcaacctcaaatctgcagtagaaactaggagacttgagtatag
taggggtagagggaatttccagtggagcggtgaaatgcgtagatattggaaagaacaccgatggcgaaggcactctactaggctattactgac
actcagagacgaaagctaggggagcaaatg

>541e8d9ee02a46b74c3adeca6a36491d

taaggaatattggtcaatggagggaactctgaaccagccatgccgcgtgcaggatgaaagccctctgggttgtaaactgcttttaacagggaag
aaaatgctatacgtgatatagtactgacggtacctgaggaataagcaccggctaactccgtgccagcagccgcggtaatacggagggtgcaag
cgttatccggaatcactgggtttaaagggtgcgtaggcggttttataagtcagtggtaaaaggttgtagctcaactataagaggtccattgatact
gtaagacttgaatcgagttgaggttggcggaatgagacatgtagcggtgaaatgcttagatatgtcatagaacaccaattgcgaaggcagctga
ctagacttagattgacgctgaggcacgaaagtgtgggtagcgaaca

>541f6821e5035ab2d63c16fa681bf389

tggggaatattggacaatgagcgaaagcttgatccagcaatatcgcgtgagtgaagaaggctattttggttgtaaaactctttcgtcgaggaaga
taatgacggtactcgaagaagaagccccggctaactccgtgccagcagccgcggtaagacggagggggctagtgttattcggaattactgggc
gtaaagggcacgtaggctgtgaattaagttgaaagtgaaatcccaaagcttaactttggaactgttttcaaaactgattcactagagttcaatag
gggatagtggaatttctagtgtagaaattaaattcttaaatattagaaggaacaccagtggcgaaggcgactatctggattgatactgacgctaa
ggtgcgaaagcatggggagcaaata

>5497318e515a8c328a68f95975d9c7d4

tagggaatcttccgcaatgggcgaaagcctgacggagcaacgccgcgtgagtgatgaaggtcttcggatcgtaaaactctgttattagggaaga
acaaatgtgtaagtaactatgcacgtcttgacggtacctaatcagaaagccacggctaactacgtgccagcagccgcggtaatacgtaggtggc
aagcgttatccggaattattgggcgtaaagcgcgcgtaggcggtttttttaagtctgatgtgaaagcccacggctcaaccgtggagggtcattgga
aactggaaaacttgagtgcagaagaggaaagtggaattccatgtgtagcggtgaaatgcgcagagatatggaggaacaccagtggcgaaggc
gactttctggtctgtaactgacgctgatgtgcgaaagcgtggggatcaaaca

>552f110db8d39cc665101679623fa17d

tggggaatcttggacaatgggcgcaagcctgatccagccatgccgcgtgagtgatgaaggccctagggtcgtaaagctctttcgccagagatga
taatgacagtatctggtaaagaagtcccggctaactccgtgccagcagccgcggtaatacggaggggactagcgttgttcggaattactgggcg
taaagcgcacgtaggcggatcagaaagtatggggtgaaatcccagggctcaaccctggaactgcctcataaactcctggtctagagttcgagag
aggtgagtggaattccgagtgtagaggtgaaattcgtagatattcggaggaacaccagtggcgaaggcggctcactggctcgatactgacgctg
aggtgcgaaagtgtggggagcaaaca

>56104f48457981616f041d0577c26179

tggggaatattgcacaatgggcgaaagcctgatgcagcgacgccgcgtgagggatgacggccttcgggttgtaaacctctttcagcagggaag
aagcgaaagtgacggtacctgcagaagaagcaccggctaactacgtgccagcagccgcggtaatacgtagggtgcgagcgttgtccggaatta
ttgggcgtaaagagcttgtaggcggtttgtcgcgtctgcagtgaaaatccgaggctcaacctcggacttgctgtgggtacgggcagactagagta
tggtaggggagaccggaattcctggtgtagcggtggaatgcgcagatatcaggaggaacaccgatggcgaaggcaggtctctgggccataact
gacgctgagaagcgaaagcatggggagcgaaca

>5760c84ea88c18318e8e806996fb4d51

tggggaatattggacaatgggcgaaagcctgatccagcaatgccgcgtgggtgaagaaggcctgcgggttgtaaagccctttcagccgggagg
aaaagcatcgggctaatatctcgatgtcttgacgttaccggcagaagaagcaccggctaactccgtgccagcagccgcggtaatacggagggtg
cgagcgttaatcggaattactgggcgtaaagcgcatgtaggcggccggataagtcgagtgtgaaaaccccgggctcaacctgggaattgcatcc
gatactgttcggctagagtctggtagagggaggcggaattcccggtgtagcggtgaaatgcgtagatatcgggaggaacaccagtggcgaagg
cggcctcctggaccaagactgacgctgaggtgcgaaagcgtggggagcaaaca

>5796a8941308656f11cee5f298cfa4cd

ttaggaatattggacaatgggggcaaccctgatccagccatgccgcgtgtgtgaagaaggccttagggttgtaaagcactttcggctgtgaagat
aatgacagtagcagaagaagaagctccggctaaattcgtgccagcagccgcggtaatacgaatggagcgagcgttgttcggaatcactgggcg
taaagcgtgcgcaggcggtcttgaaagtcaggagtgaaatcccagggcttaaccctggaattgctcttgaaactccaagactagagtactggag
aggttggcggaatttcgagtgtagcagtgaaatgcgtagatattcgaaggaacaccgatggcgtaggcagccaactggacagttactgacgctc
atgcacgaaagcgtggggatcaaaca

>586a580e56cf02c4b815e0d30cb1e230

tggggaattttccgcattgggcgaaagcctgacggagcaatgccgcgtggaggtagaaggcccacgggtcatgaacttcttttcccggagaaga
agcaatgacggtatctggggaataagcatcggctaactctgtgccagcagccgcggtaatacagaggatgcaggcgttatccggaatgattggg
cgtaaagcgtctgtaggtggctttttaagtccgccgtcaaatcccagggctcaactctggacaggcggtggaaactaccaagctggagtacggta
ggggcagagggaatttccggtggagcggtgaaatgcgtagagatcggaaagaacaccaacggcgaaagcactctgctgggccgacactgaca
ctgagagacgaaagctaggggagcgaatgggattagataccccctgtagt

>58c6e137a2022dfbdf30c2f2ef5949c4

tagggaatattggacaatgggcgaaagcctgatccagcaataccatgtgagtgatgaaggctatttaggctgtaaagctctttcatcgaggaag
ataatgacggtactcgaagaagaagccccggctaaccctgtgccagcagccgcggtaagacaggggggctagtgttattcggaatgactggg
cgtaaagggtgcgtaggcggtaaataaagttgaaggtgaaatcccaaagcttaactttggaaatgcgttcaaaactaatttactggagtatgat
agaggatagtggaatttctagtgtagagatagaattcgcaaagattagaaggaacaccagtggcgaaggcgactatctggatcaatactgacg
ctgaggcacgaaagcatggggagcaaacg

>58f3b1137cd96451070ebfeacf4c937c

ccgcggtaatacagatggggcaagtgttattcggaatgattgggcgtaaagggtacgtaggtggttttattagtcagttgtcacaaccttttaga
aaagaatcgcagctttaaaagactacaaaacttgagtttgatagaggaaagcggaattttgagtggagagatagaattcatagatattcaaagg
aacaccgaaaacgaaggtagctttctgggtcaacactgacgctcatgtacgaaagcatggggagtaaatg

>59835154e1b1073cb003929fd7684a91

taaggaattttccgcaatgggcgaaagcctgacggagcaataccgcgtgaaggatgacggcccatgggttgtaaacttcttttcttggagaaga
attctgacggtatccaaggaataagcatcggctaactccgtgccagcagccgcggtaagacgggggatgcaagcgttattcggaattattgggc
gtaaagcgtctgtaggtggtgaggaaagtgttttgttaaagatcagggcttaaccctgggaaagcagagcatactatctcacttgagttcggtag
gggcagagagaattcccagtgtagcggtgaaatgcgtagatattgggaggaagaccggtggcgaaggcgttctgctgggccgtaactgacact
gagagacgaaagctaagggagcaaata

>59aa8c6b152a2caf743a16c5cdaf4144

tggggaattttccgcaatgggcgaaagcctgacggagcaatgccgcgtggaggtagaggcccctgggtcatgaacttcttttcccggagaagaa
aaaatgacggtatccggggaataagcatcggctaactctgtgccagcagccgcggtaagacagaggatgcaagcgttatccggaatgattggg
cgtaaagcgtctgtaggtggctttttaagttcgctgtcaaataccagggcccaaccctggacaggtggtgaaaactactaagctagagtacggta
ggggcagagggaatttccggtggagcgatgaaatgcgtagagatcggaaggaacaccaacggcgaaagcactctgctgggccgacactgaca
ctgagagacgaaagctaggggagcgaatg

>5a8f0b46a094391e9436f875639034e9

tagggaatcttcggcaatggacgaaagtctgaccgagcaacgccgcgtgagtgaagaaggttttcggatcgtaaaactctgttgttagagaaga
acaagtgggagagtaactgttcccgccttgacggtatctaaccagaaagccacggctaactacgtgccagcagccgcggtaatacgtaggtggc
aagcgttgtccggatttattgggcgtaaagcgagcgcaggtggttctttaagtctgatgtgaaagcccccggctcaaccggggagggtcattgga
aactggggaacttgagtgcagaagaggagagtggaattccatgtgtagcggtgaaatgcgtagatatatggaggaacaccagtggcgaaggc
gactctctggtctgtaactgacactgaggctcgaaagcgtggggagcaaaca

>5aba2d48a9999d2a3b9c07f1465e84b7

tagggaatcttccgcaatggacgaaagtctgacggagcaacgccgcgtgtatgaagaaggttttcggatcgtaaagtactgttgttagagaaga
acaaggataagagtaactgcttgtcccttgacggtatctaaccagaaagccacggctaactacgtgccagcagccgcggtaatacgtaggtggc
aagcgttgtccggatttattgggcgtaaagcgcgcgcaggcggtcttttaagtctgatgtgaaagcccccggcttaaccggggagggtcattgga
aactggaagactggagtgcagaagaggagagtggaattccacgtgtagcggtgaaatgcgtagatatgtggaggaacaccagtggcgaaggc
gactctctggtctgtaactgacgctgaggctcgaaagcatgggtagcgaaca

>5b6b170276b8ef352f78beb0f0e0fe73

tgaggaattttccgcaatgggcgaaagcctgacggagcaataccgcgtggaggatgacggcctgtgggtcttaaactccttttcttagagaaga
agttctgacggtatctaaggaataagcatcggctaactccgtgccagcagccgcggtaatacgggggatgcaagcgttatccggaatgattggg
cgtaaagcgtctgtaggtggctttccaagtcaactgtcaaaaatcagggcttaaccttgagccggcagttgaaactatgaagcttgagttaggta
ggggcagagggaattcctagtgtagcggtgaaatgcgtagatattaggaagaacaccaatcgcgaaagcactctgctgggccatgactgacac
tgagagacgaaagctaggggagcgaatgggattagataccccagtagt

>5cf49312cbe55402c4efc855e881ec8a

tggggaattttggacaatgggcgaaagcctgatccagccatgccgcgtgcaggatgaaggccttcgggttgtaaactgcttttgtacggaacgaa
aagactccttctaataaagggagtccatgacggtaccgtaagaataagcaccggctaactacgtgccagcagccgcggtaatacgtagggtgc
aagcgttaatcggaattactgggcgtaaagcgtgcgcaggcggttatataagacagatgtgaaatccccgggctcaacctgggaactgcatttg
tgactgtatagctagagtacggcagagggggatggaattccgcgtgtagcagtgaaatgcgtagatatgcggaggaacaccgatggcgaaggc
aatcccctgggcctgtactgacgctcatgcacgaaagcgtggggagcaaaca

>5f11c3c0d2d5759a9d51de131b9cf5c8

tggggaatattgcacaatgggcgcaagcctgatgcagccatgccgcgtgtgtgaagaaggccttcgggttgtaaagcactttcagcgaggagga
aaggttagtcgctaatatcggctagctgtgacgttactcgcagaagaagcaccggctaactccgtgccagcagccgcggtaatacggagggtgc
gagcgttaatcggaattactgggcgtaaagcgtacgcaggcggccatttaagtcagatgtgaaagccccgggctcaacctgggaactgcatttg
aaactggatggctagagtgcgacagagggtggtagaatttcaggtgtagcggtgaaatgcgtagagatctgaaggaataccaatggcgaaggc
agccacctgggtcgacactgacgctcatgtacgaaagcgtgggtagcaaaca

>5f7382f2d1a279b491bfe682e999d41b

tggggaattttccgcaatgggcgaaagcctgacggagcaatgccgcgtgaaggcagaaggcccacgggtcatgaacttcttttctcggagaaga
aaaaatgacggtatctgaggaataagcatcggctaactctgtgccagcagccgcggtaagacagaggatgcaagcgttatccggaatgattgg
gcgtaaagcgtctgtaggtggcttttcaagtccgccgtcaaatccccgggctcaaccctggacaggcagtggaaactaccaagctggagtacgg
taggggcagagggaatttccggtggagcggtgaaatgcgttgagatcggaaagaacaccaacggcgaaagcactctgctgggccgacactga
cactgagagacgaaagctaggggagcaaatg

>5f8b99d96b9fb65e12c944b3f8bd38b9

tggggaattttggacaatgggcgaaagcctgatccagcaatgccgcgtgtgtgaagaaggccttcgggttgtaaagcacttttgtccggaaaga
aatccttggctctaatacagtcgggggatgacggtaccggaagaataagcaccggctaactacgtgccagcagccgcggtaatacgtagggtg
cgagcgttaatcggaattactgggcgtaaagcgtgcgcaggcggtttgctaagaccgatgtgaaatccccgggctcaacctgggaactgcattg
gtgactggcaggctagagtatggcagaggggggtagaattccacgtgtagcagtgaaatgcgtagagatgtggaggaataccgatggcgaag
gcagccccctgggccaatactgacgctcatgcacgaaagcgtggggagcaaaca

>603311b987d30368539611fdb617a67a

tggggaatcttagacaatgggggaaaccctgatctagccatgccgcgtgatcgatgaaggccttagggttgtaaagatctttcagatgggaaga
taatgacggtaccatcagaagaagccccggctaactccgtgccagcagccgcggtaatacggagggggctagcgttattcggaattactgggcg
taaagcgcacgtaggcggattggatagttggaggtgaaatcccagggctcaaccttggaactgccttcaaaacttccagtcttgaggtcgagag
aggtaagtggaattccgagtgtagaggtgaaattcgtagatattcggaggaacaccagtggcgaaggcggcttactggctcgatactgacgctg
aggtgcgaaagcgtggggagcaaaca

>61307d86c31f81363b2727ec24b3ca6a

tggggaatattgcacaatgggcgcaagcctgatgcagccatgccgcgtgtgtgaagaaggctctagggttgtaaagcactttcagttgggagga
aagcttgtagattaatactctataagtgtgacgttaccaacagaagaagcaccggctaactccgtgccagcagccgcggtaatacggagggtgc
aagcgttaatcggaattactgggcgtaaagcgcacgtaggtggtttgttaagttggatgtgaaagccccgggctcaacctgggaactgcatcca
aaactggcaaactagagtacggtagaggtgagtggaatttcctgtgtagcggtgaaatgcgtagagatgggaaggaacatcagtggcgaaggc
gactcactggactgatactgacactgaggtgcgaaagcgtgggtagcaaaca

>61de4bf54c5a7ce590210959e2d93ed7

tgaagaatattaggcaatgcacgaaagtgtgacctatcgaattagcggaaatgaagaaggctacagtagccgtaaagtttatacgtgtacaag
aattatgatttgatgtacaaagagaagtcatggcaaatacaggtgccagcagccgcggtaatacctgagtgacgagcattattcgcaataactg
ggtgtaaaggatgtgtaggaaattttaaaaaatgactgactaaattctcggcaaagatgggctttcgtttttataaatatttgagtatctatagag
agttggggtgtgtctaaatagtgataaaatactaaaatttagccattcttgccgattgtataagtaacgattcttataggttctgactctgaaacat
caaagtaagggaaataaaca

>62f1ba162f9bb059bc3045f13179dba2

tggggaattttggacaatggacgcaagtctgatccagcaataccgcgtgcaggacgaaggccttcgggttgtaaactgcttttgtacggaacga
aaagccttcttctaatacagggaggtcatgacggtaccgtaagaataagcaccggctaactacgtgccagcagccgcggtaatacgtagggtgc
aagcgttaatcggaattactgggcgtaaagcgtgcgcaggcggttgtgtaagacaggtgtgaaatccccgagctcaacttgggaattgcacttgt

gactgcacagctagagtacggcagagggggatggaattccgcgtgtagcagtgaaatgcgtagatatgcggaggaacaccgatggcgaaggc
aatcccctgggcctgtactgacgctcatgcacgaaagcgtggggagcaaaca

>64bf18c8572c61680370cfbafe7ac793

tggggaatattggacaatgggggcaaccctgatccagccatgccgcgtgagtgatgaaggccttagggttgtaaaactcttttgtccgggaagat
aatgactgtaccggaagaataagccccggctaacttcgtgccagcagccgcggtaatacgaagggggctagcgttgttcggaatcactgggcgt
aaagcgcacgtaggcggatcgttaagtcagaggtgaaatcccggagctcaacttcggaactgcctttgatactggcgatctcgagtttggaaga
ggttggtggaactgcgagtgtagaggtgaaattcgtagatattcgcaagaacaccagtggcgaaggcggccaactggtccaatactgacgctga
ggtgcgaaagcgtggggagcaaaca

>64dda94b15ad63e3875ea5a8b3ab84f2

tggggaatcttgcacaatgggcgaaagcctgatgcagaaacgccgcgtgcgggaagaaggccctagggttgtaaaccgctttcagcagggaag
aaactgacggtacctgtagaagaaggtgcggccaactacgtgccagcagccgcggtgacacgtaggcaccaagcgttgtccggatttattgggc
gtaaagagctcgtaggcggttcggtaagtcgggtgtgaaaactctgggctcaacccagagccgccacctgatactgctgtgactcgagtgcggt
aggggagcggggaattcctggtgtagcggtgaaatgcgcagatatcaggaggaacaccagtggcgaaggcgccgctctgggccgtgactgac
gctgaggagcgaaagcgtgggtagcaaaca

>66fac4b9efc92db4607478d36ae09336

tggggaattttccgcaatgggcgaaagcctgacggagcaatgccgcgtggaggtggaaggcctacgggtcgtcaacttcttttctcggagaaga
aacaatgacggtatctgaggaataagcatcggctaactctgtgccagcagccgcggtaagacagaggatgcaagcgttatccggaatgattgg
gcgtaaagcgtctgtaggtggcttttcaagtccgccgtcaaatcccagggctcaaccctggacaggcggtggaaactaccaagctggagtacgg
taggggcagagggaatttccggtggagcggtgaaatgcattgagatcggaaagaacaccaacggcgaaagcactctgctgggccgacactga
cactgagagacgaaagctaggggagcaaatgggattagataccctagtag

>671bca4fd9d9a534f5ef9ca36254bc3e

tgaggaatattggtcaatggaggcaactctgaaccagccatgccgcgtgaaggatgaaggtcctctggattgtaaacttcttttatagaagaaga
aaccccggtattcttaccgggttgacggtactctatgaataagcaccggctaactccgtgccagcagccgcggtaatacggagggtgcaagcgtt
atccggattcactgggtttaaagggagcgtaggtgggttggtaagtcagtggtgaaatctccgagcttaacttggaaactgccattgatactacc
agtcttgaatactgtggaggtaagcggaatatgtcatgtagcggtgaaatgcttagatatgacatagaacaccgatagcgaaggcagcttacta
cgcagttattgacactgaggctcgaaagcgtggggatcaaaca

>6767886def9f21be29cee68e507a353d

tggggaattttccgcaatgggcgaaagcctgacggagcaatgccgcgtggaggtagaaggcccacgggtcatgaacttcttttcccggagaaga
agcaatgacggtatctggggaataagcatcggctaactctgtgccagcagccgcggtaatacagaggatgcaagcgttatccggaatgattggg
cgtaaagcgtctgtaggtggctttttaagtccgccgtcaaatcccagggctcaactctggacaggcggtggaaactaccaagctggagtacggta
ggggcagagggaatttccggtggagcggtgaaatgcgtagagatcggaaagaacaccaacggcgaaagcactctgctgggccgacactgaca
ctgagagacgaaagctaggggagcgaatgggattagatacccgggtagt

>6809485884cd95bf31581d0154e0e253

tagggaatcttccgcaatggacgaaagtctgacggagcaacgccgcgtgagtgaagaaggttttcggatcgtaaaactctgttgtcagagaaga
acaagttggagagtaactgctccagccttgacggtatctgaccagaaagccacggctaactacgtgccagcagccgcggtaatacgtaggtggc
aagcgttgtccggatttattgggcgtaaagcgagcgcaggcggttccttaagtctgatgtgaaagcccacggctcaaccgtggaaggtcattgga
aactggggaacttgagtgcagaagaggagagtggaattccatgtgtagcggtgaaatgcgtagatatatggaggaacaccagtggcgaaggc
gactctctggtctgtaactgacgctgaggctcgaaagcgtggggagcaaacaggattagataccccggtagt

>68aad407f0c22f8c9afaa4863447f51f

tggggaatattggacaatgggcgcaagcctgatccagcaatgccgcgtgggtgaagaaggcctgcgggttgtaaagccctttaagcggggagg
aaagccggggcgtcaatagcgcctcggggtgacgttacccgcagaataagcaccggctaactccgtgccagcagccgcggtaatacgggggt
gcgagcgttaatcggaattactgggcgtaaagcgtgcgtaggcggcgcaatcagtcagccgtgaaagccccgggcttaacctgggaatggcgg
ttgagacggttgggctagagtgggctagaggatcgtggaactcccggtgtagcggtgaaatgcgtagagatcgggaagaacaccgatggcgaa
ggcagcggtctggggccacactgacgctgaggcacgacggcgtggggagcaaaca

>68bb6d5696f465d5ac396c7a6ada0250

tggggaatattgcacaatgggcgaaagcctgatgcagcgacgccgcgtgagggatgacggccttcgggttgtaaacctctttcagcagggaag
aagcgaaagtgacggtacctgcagaagaagcaccggctaactacgtgccagcagccgcggtaatacgtagggtgcgagcgttgtccggaatta
ttgggcgtaaagagcttgtaggcggtttgtcgcgtctgctgtgaaaatccggggctcaaccccggacctgcagtgggtacgggcagactagagta
tggtaggggagactggaattcctggtgtagcggtgaaatgcgcagatatcaggaggaacaccgatggcgaaggcaggtctctgggccattact
gacgctgagaagcgaaagcgtggggagcaaaca

>6906ec2c3e825267f53731bc296b3c83

tagggaatcttcggcaatggacgaaagtctgaccgagcaacgccgcgtgagtgaagaaggttttcggatcgtaaaactctgttgttagagaaga
acgttgcatagagtggaaaattatgcaagtgacggtatctaaccagaaagggacggctaactacgtgccagcagccgcggtaatacgtaggtc
ccgagcgttgtccggatttattgggcgtaaagcgagcgcaggtggtttaataagtctgatgtaaaaggcaatggctcaaccattgtgtgcattgg
aaactgttagacttgagtgcagtagaggagagtggaattccatgtgtagcggtgaaatgcgtagatatatggaggaacaccggtggcgaaagc
ggctctctggactgtcactgacactgaggctcgaaagcgtgggtagcaaaca

>6af34a43ea7caf29692704560fe79591

tggggaatattggacaatggggggaaccctgatccagcaatgccgcgtgtgtgaagaaggcctgagggttgtaaagcactttcggtggggagg
agtatatataggttaagagctaatatataggacgttacccacataagaagcaccggctaactccgtgccagcagccgcggtaatacggagggtg
cgagcgttaatcggaattactgggcgtaaagggtgcgtaggttgttgattaagttatctgtaaaatccctgggctcaacctgggcaggtcagata
agactgattgactcgagtataggagagggtagtggaatttccggtgtagcggtgaaatgcgtagagatcggaaggaacaccagtggcgaaggc
ggctacctggcctaatactgacactgaggcacgaaagcgtggggagcaaaca

>6bb7141e0deafcdada8a68f197aced90

taaggaatattggtcaatggaggcaactctgaaccagccatgccgcgtggaggatgaaggtcctctggattgtaaacttcttttatatgggacga
aaaaaggcttttctaagtcgtctgacggtaccatatgaataagcaccggctaactccgtgccagcagccgcggtaatacggagggtgcaagcgt
tatccggattcactgggtttaaagggtgcgtaggtgggttggtaagtcagtggtgaaatccccgagcttaacttgggaactgccattgatactatc
agtcttgaataccgtggaggtcggcggaatatgtcatgtagcggtgaaatgcttagatatgacatagaacaccaattgcgaaggcagctgacta
cgcggatattgacactgaggcacgaaagcgtggggatcaaaca

>6bf4208c1176bb483ed245f86e785152

tagggaatcttccgcaatggacgaaagtctgacggagcaacgccgcgtgtatgaagaaggttttcggatcgtaaagtactgttgttagagaaga
acaaggataagagtaactgcttgtcccttgacggtatctaaccagaaagccacggctaactacgtgccagcagccgcggtaatacgtaggtggc
aagcgttgtccggatttattgggcgtaaagcgcgcgcaggcggtcttttaagtctgatgtgaaagcccccggcttaaccggggagggtcattgga
aactggaagactggagtgcagaagaggagagtggaattccacg

>6c01b8d12dfe4a4f4280aacc621cc031

ccgcggtaatacgtaggtggcaagcgttgtccggatttattgggcgtaaagcgagcgcaggcggttccttaagtctgatgtgaaagcccacggct
caaccgtggaaggtcattggaaactggggaacttgagtgcagaagaggagagtggaattccatgtgtagcggtgaaatgcgtagatatatgga
ggaacaccagtggcgaaggcgactctctggtctgtaactgacgctgaggctcgaaagcgtggggagcaaaca

>6d65fe9936fdb9933802a695f197070b

tggggaattttccgcaatgggcgaaagcctgacggagcaatgccgcgtggaggtagaaggcccacgggtcatgaacttcttttcccggagaaga
agcaatgacggtatctggggaataagcatcggctaactctgtgccagcagccgcggtaatacagaggatgcaagcgttatccggaatgattggg
cgtaaagcgtctgtaggtggctttttaagtccgccgtcaaatcccagggctcaactctggacaggcggtggaaactaccaagctggagtacggta
ggggcagagggaatttccggtggagcggtgaaatgcgtagagatcggaaagaacaccaacggcgaaagcactctgctgggccgacactgaca
ctgagagacgaaagctaggggagcgaatgggattagataccccagta

>6dd8bd7675b56fedc7f1d860167f412f

tggggaattttccgcaatgggcgaaagcctgacggagcaatgccgcgtggaggtagaaggcccacgggtcatgaacttctttccccggagaag
aagcaatgacggtatctggggaataagcatcggctaactctgtgccagcagccgcggtattacagaggatgcaagcgttatccggaatgattgg
gcgtaaagcgtctgtaggtggctttttaagtccgccgtcaaatcccagggctcaactctggacaggcggtggaaactaccaagctggagtacggt
aggggcagagggaatttccggtggagcggtgaaatgcgtagagatcggaaagaacaccaacggcgaaagcactctgctgggccgacactgac
actgagagacgaaagctaggggagcgaatg

>6e16693f910b8be9b45277bd0d4dd9a4

tggggaatattggacaatgggcgaaagcctgacggagcaatctcgtgtgagggattagtctcgtgagggacggaaagtctacggattgtaaac
ctctttttcagggaggaatacaatgacgtgtatctgaagaataagcatcggctaactccgtgccagcagccgcggtaagacggaggatgcaag
tgttatccggaattattgggcgtaaagcgtttgtaggtggttaaataagtcaattgttaaatcttgaagcttaacttcaaaattgcaatcgaaacta
ttagactagagtatagtaagggtaacaggaattttcagtggagcggtgaaatgtgtagatattgaaaggaacatcagtggcgaaagcgttttact
aggttaatactgacactcagaaactaaagctaggggagcaaatg

>6ec88afe9afacb2ae5e39a2d96b638d3

tggggaatattgggcaatgggcgaaagcctgacccagcaataccgcgtgagtgatgaaggtcgtaagattgtaaaactcttttattagagaaga
ttatgacggtatctaatgaataaggactgactaacttcgtgccagcagtcgcggtaatacgaagagtccaagcgttactcggaatcactgggcgt
aaagaatgcgcaggctgttttgtaagttgataatgaaataccggagcataacttcggtgctgttatcaaaactgcaagactagagtttggtaggg
gatgatggaatttttggtgtaggggtaaaatcctttgatatcaaaaggaacgccggaggcgaaagcgatcatctgggccaatactgacgctcat
gcatgacagcgtggggagcaaaca

>6ecf0411e6cb2404165d7fb96db6c238

tagggaatcttccgcaatggacgaaagtctgacggagcaacgccgcgtgtatgaagaaggttttcggatcgtaaagtactgttgttagagaaga
acaaggataagagtaactgcttgtcccttgacggtatctaaccagaaagccacggctaactacgtgccagcagccgcggtaatacggagggtgc
aagcgttatccggaatcactgggtttaaagggtgcgtaggtgggctaataagtcagaggtgaaagcccgcagcttaactgcggaattgcctttga
tactgttagtcttgaatcaggttgaggtatgcggaatgtggcatgtagcggtgaaatgcgtagatatgccatagaacaccgattgcgaaggcagc
atactggccctgtattgacactgaggcacgaaagcgtggggagcgaaca

>6f87dd84cb1ce4668e15e837607db316

tagggaatcttccgcaatggacgaaagtctgacggagcaacgccgcgtgtatgaagaaggttttcggatcgtaaagtactgttgttagagaaga
acaaggataagagtaactgcttgtcccttgacggtatctaaccagaaagccacggctaactacgtgccagcagccgcggtaatacgtagggtgc
gagcgttaatcggaattactgggcgtaaagcgtgcgcaggcggttgtgtaagacaggtgtgaaatccccgggcttaacctgggaactgcgcttgt
gactgcacagctagagtacggcagaggggggtggaattccacgtgtagcagtgaaatgcgtagagatgtggaggaacaccgatggcgaaggc
agccccctgggcctgtactgacgctcatgcacgaaagcgtggggagcaaaca

>70e6f31afa70525ebbeb0bce77ba2121

tagggaatcttccgcaatggacgaaagtctgacggagcaacgccgcgtgagcgaagaaggtcttcggatcgtaaagctctgttgtcagggaag
aacaagtaccgttcgaacagggcggtaccttgacggtacctgaccagaaagccacggctaactacgtgccagcagccgcggtaatacgtaggt
ggcaagcgttgtccggaattattgggcgtaaagcgcgcgcaggcggtctcttaagtctgatgtgaaagcccacggctcaaccgtggagggtcatt
ggaaactgggagacttgagtgcagaagaggagagcggaattccacgtgtagcggtgaaatgcgtagagatgtggaggaacaccagtggcgaa
ggcggctctctggtctgtaactgacgctgaggcgcgaaagcgtggggagcgaaca

>7216595d94ee0859ec044530c2b70c7f

tggggaatcttgcgcaatgggcggaagcctgacgcagcgacgccgcgtgagggatgacggccttcgggttgtaaacctctttcagcagggaag
aagcggaagtgacggtacctgcagaagaagcaccggctaactacgtgccagcagccgcggtaatacgtagggtgcaagcgttgtccggaatta
ttgggcgtaaagagctcgtaggcggtctgtcgcgtctgctgtgaaaacttcgggctcaacccgaagctggcagtgggtacgggcggactggagt
gcagtaggggagactggaattcctggtgtagcggtgaaatgcgcagatatcaggaggaacaccggtggcgaaggcgggtctctgggctgttac
tgacgctgaggagcgaaagcgtggggagcgaaca

>731fbab381606c13d39053eb1c805648

tgaggaatattcggcaatgggggcaaccctgaccgagcaacgccgcgtggaggatgacggttttcggattgtaaactccttttctcagggaaga
gcgaggacggtacctgaggaataagtcacggctaactacgtgccagcagccgcggtaatacgtaggtggcaagcgttatccggaattactggg
cgtaaagcgcacgcaggtggctgcttaagtctgacgtgaaagctcctggcttaactgggagaggtcgttggagactgggcggcttgaggcgagg
agaggggtgtagaattcccggtgtagtggtggaatgcgtagagatcgggaggaataccagtggcgaaagcggcaccctggccttggcctgacg
ctcaggtgcgaaagcgtggggagcgaacg

>735afa4d80e8c4aa9906b0445ef9e9a6

tgaggaatattggacaatgaacgtaagtttgatccaacaatattgctttggtgaagaaaattaattttgtaaaaccatacttaatttaaagataat
gacataaaatttaagtaagtcctgactaatttcgtgccagcagtcgcggtgatacggaaaggacaagtgttatttgatttaactgggtgtaaagtg
tgtgtagatgattaaaataataataaaaaaaaaaattattttttaaataattaattttattatatttttaatttgagttatagtgtagtttgcagaa
ctttaagtgtagaggtaaaattcttaaaaatttaaggtactttcaagaaggtgaaaacagcatgctaattataaactgacattgaagcacaaaag
cataggtatcaaatg

>73966f8498e3223dfb1b3af2d7856de0

tgaggaatattggacaatgggcgcaagcctgatccagccatgccgcgtgcaggatgaagcatctatggtgtgtaaactgcttttgtacgagaag
aaacactgcttcgtgaagcagcttgacggtatcgtaagaataaggatcggctaactccgtgccagcagccgcggtaatacggaggatccaagc
gttatccggaatcattgggtttaaagggtccgtaggcgggccagtaagtcagtggtgaaatctcccggctcaaccgggaaatggccattgatact
gctggtcttgaattattaggaagtaactagaatatgtagtgtagcggtgaaatgcttagagattacatggaataccaattgcgaaggcaggttac
tactaatggattgacgctgatggacgaaagcgtgggtagcgaaca

>7484615e4461354c073c50c069fcd5c5

tggggaatcttagacaatgggggcaaccctgatctagccatgccgcgtgagtgatgaaggccttagggttgtaaagctctttcagctgggaagat
aatgacggtaccagcagaagaagccccggctaactccgtgccagcagccgcggtaatacggagggggctagcgttgttcggaattactgggcg
taaagcgcacgtaggcggactggaaagttggaggtgaaatcccagggctcaaccttggaactgccttcaaaactatcagtctggagttcgagag
aggtgagtggaattccgagtgtagaggtgaaattcgtagatattcggaggaacaccagtggcgaaggcggctcactggctcgatactgacgctg
aggtgcgaaagcgtggggagcaaaca

>755d78b08ff387893f0deb62c8f0e6ec

tggggaatattgcacaatgggcgaaagcctgatgcagcgacgccgcgtgagggatgacggccttcgggttgtaaacctctttcagcagggaag
aagcgaaagtgacggtacctgcagaagaagcaccggctaactacgtgccagcagccgcggtaatacgtagggtgcgagcgttgtccggaatta
ttgggcgtaaagagcttgtaggcggtttgtcgcgtctgcagtgaaaatccggggctcaaccccggacttgctgtgggtacgggcagactagagtg
tggtaggggagaccggaattcctggtgtagcggtggaatgcgcagatatcaggaggaacaccgatggcgaaggcaggtctctgggccacaact
gacgctgagaagcgaaagcatggggagcgaacaggattagataccccagtag

>76000149a575735de0dcf5d2db530790

tggggaattttccgcaatgggcgaaagcctgacggagcaatgccgcgtggaggtggaaggcctacgggtcgtcaacttcttttctcggagaaga
aacaatgacggtatctgaggaataagcatcggctaactctgtgccagcagccgcggtaagacagaggatgcaagcgttatccggaatgattgg
gcgtaaagcgtctgtaggtggcttttcaagtccgccgtcaagtcccagggctcaaccctggacaggcggtggaaactaccaagctggagtacgg

taggggcagagggaatttccggtggagcggtgaaatgcattgagatcggaaagaacaccaacggcgaaagcactctgctgggccgacactga
cactgagagacgaaagctaggggagcaaatg

>76c6d8f0905c4d929ed1279bc69858f9

taaggaatattggtcaatggacgaaagtctgaaccagccatcccgcgtgcaggatgaaggtcctatggattgtaaactgcttttagcagagaag
aaaagcatccttttaagggtgtctgacggtatctgcggaataagcaccggctaactccgtgccagcagccgcggtaatacggagggtgcaagcg
ttatccggaatcactgggtttaaagggtgcgtaggcggaatattaagtcagtggtgaaagcctgtcgcttaacgatagaactgccattgatactg
atattcttgaattgggttgaggttagcggaatgtgacatgtagcggtgaaatgcatagatatgtcatggaacaccaattgcgaaggcagctagct
gggccttgattgacgctgaggcacgaaagcgtgggtagcgaaca

>76f0f8255428fb1f026f84aef1e0f8a0

taaggaatattggtcaatggacggaagtctgaaccagccatcccgcgtgcaggatgactgccctatgggttgtaaactgcttttagcagagacga
aacgccttcatttatgagggtctgacggtatctgcagaataagcaccggctaactccgtgccagcagccgcggtaatacggagggtgcaagcgt
tatccggaatcactgggtttaaagggtgcgtaggcggatatttaagtcagtggtgaaatcttgtcgcttaacgataaaattgccattgatactgaa
tgtcttgaattgggttgaggttagcggaatgtgacatgtagcggtgaaatgcatagatatgtcatggaacaccaattgcgaaggcagctagctgg
gccttgattgacgctgaggcacgaaagcgtgggtagcgaaca

>773b3812f142ad767f3859237e8f8515

tagggaatcttccgcaatgggcgaaagcctgacggagcaacgccgcgtgagtgatgaaggtcttcggatcgtaaaactctgttattagggaaga
acaaatgtgtaagtaactatgcacgtcttgacggtacctaatcagaaagccacggctaactacgtgccagcagccgcggtaatacgtaggtggc
aagcgttatccggaattattgggcgtaaagcgcgcgtaggcggtttttttaagtctgatgtgaaagcccacggctcaaccgtggagggtcattgga
aactggaaaacttgagtgcagaagaggaaagtggaattccatgtgtagcggtgaaatgcgcagagatatggaggaacaccagtggcgaagtc
gactttctggtctgtaactgacgctgatgtgcgaaagcgtggggatcaaaca

>78c0834c53fcc6c1960f7b3740fd9a61

taaggaatattgcgcaatgggcgaaagcctgacgcagcgacgccgcgtgggggatgaaggtcttcggattgtaaacccctttcgggagggaag
atggagcgagcaatcgttcggacggtacctccagaagcagccacggccaacttcgtgccagcagccgcggtaatacgaaggtggcaagcgttg
ttcggattcactgggcgtacagggtgtgtaggcggtttggtaagccttctgttaaagcttcgggcccaacccggaaagcgcagagggtactgcca
ggctagagggtgggagaggagcgcggaattcccggtgtagcggtgaaatgcgtagagatcgggaggaaggccggtggcgaaggcggcgctct
ggaacatgcctgacgctgagacacgaaagcgtggggagcaaaca

>7eed10c4fffc3fd0a4c865a359fec1df

tggggaatattgcacaatgggcgaaagcctgatgcagcaatgccgcgtgattgaagaaggccttagggttgtaaagatcttttaccaagaatga
taatgacagtacttggggaataaggaccggctaacttcgtgccagcacccgcggtaatacgaagggtccaagcgttactcggatttactgggcgt
aaagtgcgcgtaggctgattggcaagtcgaaagttaaatccccgggctcaacctgggaatcgctttcgaaactgcctttctagagtttggtagag
agaagtggaattcttagtggaggggtaaaatccgtagatattaagaggaacaccggaggcgaaggcgacttcttgggccaatactgacgctga
ggcgcgaaggcgtggggagcaaacg

>8173390115e64057c54bd029bf900023

tggggaattttccgcaatgggcgaaagcctgacggagcaatgccgcgtggaggtagaaggcccacgggtcatgaacttcttttcccggagaaga
agcaatgacggtatctggggaataagcatcggctaactctgtgccagcagccgcggtaatacagaggatgcaagcgttatctggaatgattggg
cgtaaagcgtctgtaggtggctttttaagtccgccgtcaaatcccagggctcaactctggacaggcggtggaaactaccaagctggagtacggta
ggggcagagggaatttccggtggagcggtgaaatgcgtagggatcggaaagaacaccaacggcgaaagcactctgctgggccgacactgaca
ctgagagacgaaagctaggggggcgaatg

>81ef04fc448a46c418860d949afca5be

tggggaatattggacaatgggcgcaagcctgatccagccatgccgcgtgagtgatgaaggccctagggttgtaaagctctttcaccggtgaaga
taatgacggtaaccggagaagaagccccggctaacttcgtgccagcagccgcggtaatacgaagggggctagcgttgttcggaattactgggc
gtaaagcgcacgtaggcgggtatttaagtcaggggtgaaatcccagagctcaactctggaactgcctttgatactgggtacctagagtatggaa
gaggtaagtggaattccgagtgtagaggtgaaattcgtagatattcggaggaacaccagtggcgaaggcggcttactggtccattactgacgct
gaggtgcgaaagcgtggggagcaaaca

>828a4757876f251a1afe03b52c07441e

tagggaatcttccgcaatggacgaaagtctgacggagcaacgccgcgtgtatgaagaaggttttcggatcgtaaagtactgttgttagagaaga
acaaggataagagtaactgcttgtcccttgacggtatctaaccagaaagccacggctaactacgtgccagcagccgcggtaatacgtaggtggc
aagcgttgtccggatttattgggcgtaaagcgcgcgcaggcggtcttttaagtctgatgtgaaagcccccggcttaaccggggagggtcattgga
aactggaagactggagtgcagaagaggagagtggaattccacgtgtagcggtgaaatgcgtagatattcggaggaacaccagtggcgaaggc
ggctcactggctcgatactgacgctgaggtgcgaaagtgtggggagcaaaca

>832a3e16b70814d1faf9e67ac5c26fee

taaggaatattggtcaatggacggaagtctgaaccagccatcccgcgtgcaggatgactgccctatgggttgtaaactgcttttagcagagacga
aacgccttcatttatgagggtctgacggtatctgcagaataagcaccggctaactccgtgccagcagccgcggtaatacggagggtgcaagcgt
tatccggaatcactgggtttaaagggtgcgtaggcggatatttaagtcagtggtgaaatcttgtcgcttaacgataaaattgccattgatactgaa
tgtcttgaattgggttgaggttagcggaatgtgacatgtagcggtgaaatgcatagatatgtcatggaacaccaattgcgaaggcagctagctgg
gccttgattgacgctgaggcacgaaagcgtgggtagcgaacaggattagataccccgggtagtc

>83ec7f7576dc9d09fb87f1d5d92fdaf2

tggggaattttccgcaatgggcgaaagcctgacggagcaatgccgcgtggaggtagaaggcccacgggtcgtgaacttcttttcccggagaaga
agcaatgacggtatctgaggaataagcatcggctaactctgtgccagcagccgcggtaagacagaggatgcaagcgttatctggaatgattgg
gcgtaaggcgtctgtaggtggcttttcaagtccgccgtccaatcccagagctcaaccctggacaggcggtggaaactaccaagctggagtacgg
taggggcagagggaatttccggtggagcggtgaaatgcgtagagatcggaaagaacaccaacggcgaaagcaccctgctgggccgacactga
cactgagagacgaaagctaggggagcgaatg

>8427b8367229d57c683a805c8242dfe1

taaggaatattggtcaatgggcgcaagcctgaaccagccatgccgcgtgaaggatgaaggccctatgggttgtaaacttcttttgtacgggaaa
aaacctctggtcgtgtatcagagctgatggtaccgtaagaataagcatcggctaactccgtgccagcagccgcggtaatacggaggatgcaagc
gttatccggattcattgggtttaaagggtgcgcaggcggaataataagtcagcggtgaaagcctacagcttaactgtagaactgccattgatact
gttattcttgagtgttgttgaggttggcggaatgtgtcatgtagcggtgaaatgcttagatatgacacagaacaccgatcgcgaaggcagctaac
caagcaacaactgacgctcatgcacgaaagcgtggggatcaaaca

>847179e322a6d4463b4f0aec23812ef3

tggggaatcttggacaatgggcgaaagcccgatccagcaatatcgcgtgagtgaagaagggcaatgccacttgtaaagctctttcgtcgagtgc
gcgatcatgataggactcgaggaagaagccccggctaactccgtgccagcagccgcggtaagacggtgggggcaagtgttcttcggaatgact
gggcgtaaaggacacgtaggcggtgaatcgggttgaaagtgaaagtcgccaaaaagtggcggaatgctctcgaaaccaattcacttgagtgag
acaaaggagagtggaatttcgtgtgtaggggtgaaatccgtagatctacaaaggaacaccaaaagcgaaggcagctctctgggtccctaccga
tgctggggtgcgaaagcatggggagcgaaca

>849c8179a62919a1b5c4766d089df22c

tggggaatcttagacaatgggggcaaccctgatctagccatgccgcgtgagtgatgaaggccttagggttgtaaagctctttcagctgggaagat
aatgacggtaccagcagaagaagccccggctaactccgtgccagcagccgcggtaatacggagggggctagcgttgttcggaattactgggcg
taaagcgcacgtaggcggaccggaaagttgggggtgaaatcccggggctcaaccccggaactgccttcaaaactactggtctggagttcgaga
gaggtgagtggaattccgagtgtagaggtgaaattcgtagatattcggaggaacaccagtggcgaaggcggctcactggctcgatactgacgct
gaggtgcgaaagcgtggggagcaaaca

>84bb310929a2055b527f353254083568

caaggaattttccgcaatgggcgaaagcctgacggagcaacgccgcgtgcgggatgacgcttttcggagtgtaaaccgctgttcggggggacg
aagcactgacggtacccccggaggaaggcccggctaactacgtgccagcagccgcggtaatacgtaggggtcgagcgttgtccggagttactg
ggcgtaaagcgtgcgcaggcggctcatcgcgctcggcgtgaaagcccccggctcaaccggggagggtcgtcgaggacgggtgagcttgagggt
atcaggggttggtggaattcccggtgtagtggtgaaatgcgtagagatcgggaggaacacccgtggcgaaggcggccaactgggatacacctg
acgctgaggcacgaaggcgtggggagcgaacg

>84c412bb52078c7ce2172d30d6c90096

tggggaatattggacaatggggggaaaccctgatccagcaacgccgcgtggaggatgaaggtcttcggattgtaaactcctgttaggggggacg
aagggatgtcggcgaacagtcggcaaacttgacggtacccccagaggaagccccggctaactctgtgccagcagccgcggtaatacagaggg
ggcaagcgttattcggaattattgggcgtaaagggcgcgtaggcggccttgtaagtcttgggtgaaatccctcagctcaactgaggaactgcctc
ggatactgcaaggcttgaggccgggagagggtagtggaattcccagtgtagcggtgaaatgcgtagatattgggaggaacaccggtggcgaag
gcggctacctggaccggttctgacgctgatgcgcgaaagcgtggggagcaaaca

>84dd907bab2a29dd7f09f33838146da4

tagggaatcttccgcaatggacgaaagtctgacggagcaacgccgcgtgtatgaagaaggttttcggatcgtaaagtactgttgttagagaaga
acaaggataagagtaactgcttgtcccttgacggtatctaaccagaaagccacggctaactacgtgccagcagccgcggtaatacgtaggtggc
aagcgttgtccggatttattgggcgtaaagcgagcgcaggcggttccttaagtctgatgtgaaagcccccggctcaaccggggagggtcattgg
aaactggggaacttgagtgcagaagaggagagtggaattccatgtgtagcggtgaaatgcgtagatatatggaggaacaccagtggcgaagg
cgactctctggtctgtaactgacgctgaggctcgaaagcgtgggtagcaaaca

>853baa5b9e0e66697ddf03b73990e9ae

tggggaatcttgcacaatgggcgaaagcctgatgcagcaacgccgcgtgcgggaagaaggccctagggttgtaaaccgctttcagcagggaag
aaactgacggtacctgtagaagaaggtgcggccaactacgtgccagcagccgcggtgacacgtaggcaccaagcgttgtccggatttattgggc
gtaaagagctcgtaggcggttcggtaagtcgggtgtgaaaactctgggctcaacccagagccgccacctgatactgctgtgactcgagtgcggt
aggggagcggggaattcctggtgtagcggtgaaatgcgcagatatcaggaggaacaccagtggcgaaggcgccgctctgggccgtgactgac
gctgaggagcgaaagcgtgggtagcaaaca

>85a950584e63a02aa3389cab382a3d2a

tggggaatattgcacaatgggcgaaagcctgatgcagcgacgccgcgtgagggatgacggccttcgggttgtaaacctctttcagcagggaag
aagcgaaagtgacggtacctgcagaagaagcaccggctaactacgtgccagcagccgcggtaatacgtagggtgcgagcgttgtccggaatta
ttgggcgtaaagagcttgtaggcggtttgtcgcgtctgctgtgaaaatccgggactcaattccggacttgcagtgggtacgggcagactagagta
cggtaggggagactggaattcctggtgtagcggtgaaatgcgcagatatcaggaggaacaccgatggcgaaggcaggtctctgggccgtaact
gacgctgagaagcgaaagcatggggagcgaaca

>85d654dd53d5fe83a6b2ff8bc4c19c4f

taaggaatattccgcaatgggcgaaagcctgacggagcgacaccgcgtggaggatgacggctctagggttgtaaactccttttctgagggaaga
agatctgacggtacctcaggaataagcaccggctaattacgtgccagcagccgcggtaatacgtaaggtgcaagcattatccggaattattggg
cgtaaagcgtccgcaggcggtttgacaagtcagttattaaataccggagctcaacttcggtgctgtaaccgaaactgttaaactagaagatagg
agaggtgtgtagaatgctacaagtaggggtaaaatccgtagatatgtagtggaataccaaaagcgaaggcagcacactggactattcttgacg
ctcagggacgaaagcgtaggtagcaaaac

>8619181b69516743cde8331315c654f0

tgaggaattttccgcaatgggcgaaagcctgacggagcaataccgcgtgaaggatgaaggcctatgggttgtaaacttcttttctcagagaaga
attttgacggtatctgaggaataagcatcggctaactctgtgccagcagccgcggtaagacagaggatgcaagcgttatccggattaattgggc
gtaaagcgtctgtaggtggtttatcaagtcttttgtcaaagatcagggcctaaccctgggcaggcagaggaaactagtagactagagttcggtag

gggcagagggaattcccggtggagcggtgaaatgcgtagagatcgggaggaacaccaaaggcgaaggcactctgctgggccgcaactgacac
tgagagacgaaagcgaggggagcaaatg

>8721e39bcd9e1b4021357893c56e6649

tggggaatattgcacaatgggcgaaagcctgatgcagcgacgccgcgtgagggatgaaggccttcgggttgtaaacctctttcagcagggaag
aagcgaaagtgacggtacctgcagaagaagcaccggctaactacgtgccagcagccgcggtaatacgtagggtgcgagcgttgtccggaatta
ttgggcgtaaagagcttgtaggcggtttgtcgcgtctgctgtgaaaatccggggctcaaccccggacttgcagtgggtacgggcagactagagtg
tggtaggggagactggaattcctggtgtagcggtgaaatgcgcagatatcaggaggaacaccaatggcgaaggcaggtctctgggccaccact
gacgctgagaagcgaaagcgtggggagcgaaca

>8727a65b7a8366be1a004d8eae91ed96

tagggaatcttccgcaatggacgaaagtctgacggagcaacgccgcgtgtatgaagaaggttttcggatcgtaaagtactgttgttagagaaga
acaaggataagagtaactgcttgtcccttgacggtatctaaccagaaagccacggctaactacgtgccagcagccgcggtaatacgtaggtggc
aagcgttgtccggatttattgggcgtaaagcgcgcgcaggcggtcttttaagtctgatgtgaaagcccccggcttaaccgg

>88c12bf29fdd7b2d0f127d2b0d6a958f

tagggaatcttccgcaatggacgaaagtctgacggagcaacgccgcgtgtatgaagaaggttttcggatcgtaaagtactgttgttagagaaga
acaaggataagagtaactgcttgtcccttgacggtatctaaccagaaagccacggctaactacgtgccagcagccgcggtaatacgtaggtggc
aagcgttgtccggatttattgggcgtaaagcgcgcgcaggcggtcttttaagtctgatgtgaaagcccccggcttaaccggggagggtcattgga
aactggaagactggagtgcagaagaggagagtggaattccacgtgtagcggtgaaatgcgtagatatgtggaggaacaccagtggcgaaggc
gactctctggtctgtaactgacgctgaggcgcgaaagcgtggggagcaaacaggattagataccccctgtag

>88e6446c09d3fe93253498a4d2f9a0c5

tgaggaattttctgcaatgggcgaaagcctgacagagcaataccgcgtgagggatgaagacttactgagttgtaaacctcggtaccttaaggaa
gaagatctgacggtacttaaggtggaaagcatcggctaactccgtgccagcagccgcggtaagacgggggatgcaagtgttatccggatttact
gggcgtaaagcgtctgcaggtggtttcttaagtctactgttaaatcttgaggctcaacctcaaatctgcagtagaaactaggagacttgagtatag
taggggtagagggaatttccagtggagcggtgaaatgcgtagatattggaaagaacaccgatggcgaaggcactctactgggctattactgac
actcagagacgaaagctaggggagcaaatg

>890412537f45fcad06a7d20e4ebfd98a

tggggaattttggacaatgggcgaaagcctgatccagccatgccgcgtgcaggatgaaggccttcgggttgtaaactgcttttgtacggaacgaa
aaggtcttttctaataaagaaggctcatgacggtaccgtaagaataagcaccggctaactacgtgccagcagccgcggtaatacgtagggtgca
agcgttaatcggaattactgggcgtaaagcgtgcgcaggcggttatataagacagatgtgaaatccccgggctcaacctgggaactgcattagt
gactgtatagctagagtgcggcagagggggatggaattccgcgtgtagcagtgaaatgcgtagatatgcggaggaacaccgatggcgaaggc
aatcccctgggcctgcactgacgctcatgcacgaaagcgtggggagcaaaca

>89c3e82448157f26115944d903509787

tagggaatcttccgcaatggacgaaagtctgacggagcaacgccgcgtgtatgaagaaggttttcggatcgtaaagtactgttgttagagaaga
acaaggataagagtaactgcttgtcccttgacggtatctaaccagaaagccacggctaactacgtgccagcagccgcggtaatacgtaggtggc
aagcgttgtccggatttattgggcgtaaagcgcgcgcaggcggtcttttaagtctgatgtgaaagcccccggcttaaccggggagggtcattgga
aactggaagactggagtgcagaagaggagagtggaattccacgtgtagcggtgaaatgcgtagatatgcggaggaacaccgatggcgaaggc
aatcccctgggcctgcactgacgctcatgcacgaaagcgtggggagcaaaca

>8a6829c8525f949e5a98f6ac78ca6b14

tggggaattttccgcaatgggcgaaagcctgacggagcaatgccgcgtggaggtagaaggcccacgggtcatgaacttcttttcccggagaaga
agcaatgacggtatctggggaataagcatcggctaactctgtgccagcagccgcggtaatacagaggatgcaagcgttatccggaatgattggg
cgtaaagcgtctgtaggtggctttttaagtccgccgtcaaatcccagggctcaactctggacaggcggtggaaactaccaagctggagtacagta

ggggcagagggaatttccggtggagcggtgaaatgcgtagagatcggaaagaacaccaacggcgaaagcactctgctgggccgacactgaca
ctgagagacgaaagctaggggagcgaatg

>8c9da229c2301074e8bc5cd4c6951914

tgaggaattttccgcaatgggcgaaagcctgacggagcaataccgcgtggaggatgacggcctgtgggtcttaaactccttttcttagagaaga
agttctgacggtatctaaggaataagcatcggctaactccgtgccagcagccgcggtaatacgggggatgcaagcgttatccggaatgattggg
cgtaaagcgtctgtaggtggctttccaagtcaactgtcaaaaatcagggcttaaccttgagccggcagttgaaactatgaagcttgagttaggta
ggggcagagggaattcctagtgtagcggtgaaatgcgtagatattaggaagaacaccaatcgcgaaagcactctgctgggccatgactgacac
tgaga

>8d32dd3b7283c5f6059cf82f62d4d4c3

tggggaatattggacaatgggcgaaagcctgatccagcaataccgcgtgagtgatgacggccttcgggttgtaaagctcttttagtagggaaga
taatgacggtacctacagaaaaagcaccggctaacttcgtgccagcagccgcggtaatacgaagggtgctagcgttgttcggaatcattgggcg
taaagggtgcgtaggcggtttgacaagtcagttgtgaaagcccagggctcaaccctggaactgcaattgaaactgttagactagaatttggtgg
gggataatagaattcctagtgtaggggtgaaatccgtagatattaggaggaataccagaggcgaaggcgattgtctataccaatattgacgctg
aggcacgaaagcgtggggagcaaaca

>8e98d6ad2a7a87f80737c4ddb1a37c09

tagggaatattgggcaatgggcgaaagcctgacccagccatgccgcgtgcaggatgaaggcgctcagcgttgtaaactgcttttatctgggaag
aaaagtatgcctgcgggtgtatgtgacggtaccagaggaataagcaccggctaactccgtgccagcagccgcggtaatacggagggtgcaagc
gttgtccggatttattgggtttaaagggtgcgtaggtggtagcccaagtctggtttgaaagctggtcgctcaacgatcagatgtggctggaaactg
ggttacttgaatggattggcggtagccggaatgggtcatgtagcggtgaaatgcatagatatgacccggaaccccgattgcgaaggcaggctac
tacgatttgattgacactgaggcacgagagcatgggtagcgaaca

>8f923ea037326c333eda51ef9b127412

taaggaatttttcgcaatgggggaaaccctgacgaagcaacaccgcgtgagggatgaaggccttttggttgtaaacttctttttgagggaataa
atatgaaggtacctcaagaataagcatcggctaactacgtgccagcagccgcggtaagacgtaggatgcaagcgttatccggaattattgggcg
taaagagtctgtaggcgaattgttaagtaggatgttaaagaccagtgcctaacactgggaaggtatcctaaactggcaaaatagagtatgacag
aggcaagtagaattctgtgtgtaggggtaatatccgtagatatacagaggaataccaaaagcgaaggcagcttgctgggtcattactgacgctc
aaggacgaaagcgtggggagcaaaca

>8fb136cf860905ec86a2f52528331b5c

tggggaatcttggacaatgggcgcaagcctgatccagccatgccgcgtgagtgatgaaggccctagggtcgtaaagctctttcgccagggatga
taatgacagtacctggtaaagaaaccccggctaactccgtgccagcagccgcggtaatacggagggggttagcgttgttcggaattactgggcg
taaagcgcgcgtaggcggattagtcagtcagaggtgaaatcccagggctcaaccctggaactgcctttgatactgctagtcttgagttcgagaga
ggtgagtggaattccgagtgtagaggtgaaattcgtagatattcggaggaacaccagtggcgaaggcggctcactggctcgatactgacgctga
ggtgcgaaagtgtggggagcaaaca

>8fc3742c23b5314516453a96b9f789d3

caaggaattttccacaatggacgaaagtctgatggagctacgccgcgtgcgggatgacgcctttcggggtgtaaaccgcttttcatagggacga
atacaatgacggtacctatggaataaggatcggctaactacgtgccagcagccgcggtaagacgtaggatccgagcgttgtccggagttactgg
gcgtaaagcgagcgcaggcggttttgtacatcggcggtgaaagccccccgctcaacgggggagggtccgtcgagatggcaagactggaggctg
ggagaggtcggtggaattccgggtgtagtggtgaaatgcgtagatatccggaggaacaccagtggcgaaggcggccgactggaccagacctg
acgctaaggctcgaaggcgtggggagcgaacg

>9002efc8d0aae0e00570a0ef34a8f2f4

tggggaattttccgcaatgggcgaaagcctgacggagcaatgccgcgtggaggtagaaggcccacgggtcgtgaacttcttttcccggagaaga
agcaatgacggtatctggggaataagcatcggctaactctgtgccagcagccgcggtaatacagaggatgcaagcgttatccggaatgattggg
cgtaaagcgtctgtaggtggctttttaagtccgccgtcaaatcccagggctcaaccctggacaggcggtggaaactaccaagctggagtacggt
aggggcagagggaatttccggtggagcggtgaaatgcgtagagatcggaaagaacaccaacggcgaaagcactctgctgggccgacactgac
actgagagacgaaagctaggggagcgaatg

>905101afe6a987f895da576d843421f4

tggggaatattggacaatgggcgcaagcctgatccagccatgccgcgtgagtgatgaaggccttagggttgtaaagctctttcagtggggaaga
taatgacggtacccacagaagaagccccggctaacttcgtgccagcagccgcggtaatacgaagggggctagcgttgttcggatttactgggcg
taaagcgcacgtaggcggtttgttaagtcaggggtgaaatcccggagctcaactccggaactgcctttgatactggcaagctagagtccggaag
aggtaagtggaactcctagtgtagaggtggaattcgtagatattaggaagaacaccagtggcgaaggcggcttactggtccggaactgacgctg
aggtgcgaaagcgtggggagcaaaca

>908f448f4f59b402848848bdea0afb0e

tcgcggtgatacggagaggacgagtgttacttgtattgactgggtgtaaagcgtatgtagacgtctaaaaaagtttagatttaaaaatttagttat
aactaaaaaatttctttttatttttagattgagttataaggaagattacagaattttaagtgttaggggtaaaatccgtttgaaatttaaaagacta
tcaattggcgaaggcagtaatctatttataactgacgttgagatacagaagcataggtatcgaaca

>91012b39de4d8b24803ecbb66e792649

tagggaatcttccgcaatggacgaaagtctgacggagcaacgccgcgtgtatgaagaaggttttcggatcgtaaagtactgttgttagagaaga
acaaggataagagtaactgcttgtcccttgacggtatctaaccagaaagccacggctaactacgtgccagcagccgcggtaatacgtaggtggc
aagcgttgtccggatttattgggcgtaaagcgcgcgcaggcggtcttttaagtctgatgtgaaagcccccggcttaaccggggagggtcattgga
aactggaagactggagtgcagaagaggagagtggaattccacgtgtagcggtgaaatgcgtagagatctgaaggaataccggtggcgaaggc
ggccccctggacagatactgacactcagatgcgaaagcgtggggagcaaaca

>923513ea0a7374d4a1c8d0e14c18f370

tccggaatcttggacaattggcgaaagccagatccagccatattacgtgaaggatgaagactttttggttgtaaacttctttactttttaaagtta
aagagtataaaaagccccggctaaatctgtgccagcagccgcggtaatacagatggggcaagtgttattcggaatgattgggcgtaaagggta
cgtaggtggttttattagtcagttgtcacaacctttttagaaaagaatcgcagctttaaaagactacaaaacttgagtttgatagaggaaagcgg
aattttgagtggagagatagaattcatagatattcaaaggaacaccgaaaacgaaggtagctttctgggtcaacactgacgctcatgtacgaaa
gcatggggagtaaatgggattagataccctagta

>926a0f4e5e66b87f67782220da523ccb

tagggaatcttccgcaatggacgaaagtctgacggagcaacgccgcgtgtatgaagaaggttttcggatcgtaaagtactgttgttagagaaga
acaaggataagagtaactgcttgtcccttgacggtatctaaccagaaagccacggctaactacgtgccagcagccgcggtaatacgtaggtggc
aagcgttgtccggatttattgggcgtaaagcgcgcgcaggcggtcttttaagtctgatgtgaaagcccccggcttaaccggggagggtcattgga
aactggaagactggagtgcagaagaggagagtggaattccacgtgtagcggtgaaatgcgtagatataggaaggaacaccagtggcgaagg
cgactacctggactgatactgacactgaggtgcgaaagcgtggggagcaaaca

>927e2df075c0c36f471872f7090fd2e3

tgaggaatattggacaatgggcgcaagcctgatccagccatgccgcgtgcaggatgaagcatctatggtgtgtaaactgcttttgtacgagaag
aaacactgcttcgtgaagcagcttgacggtatcgtaagaataaggatcggctaactccgtgccagcagccgcggtaatacggaggatccaagc
gttatccggaatcattgggtttaaagggtccgtaggtggataattaagtcagaggtgaaatcctgcagctcaactgtagaattgcctttgatactg
gttatcttgagtcattatgaagtggttagaatatgtagtgtagcggtgaaatgcatagatattacatagaataccaattgcgaaggcagatcacta
ataatgtactgacactgatggacgaaagcgtggggagcgaaca

>934208deb7b3c30ce40a90260bd65058

ccaccttacctacggggggctgcagtgaggaattttccgcaatgggcgaaagcctgacggagcaataccgcgtggaggatgacggcctgtgggt
cttaaactccttttcttagagaagaagttctgacggtatctaaggaataagcatcggctaactccgtgccagcagccgcggtaatacgggggatg
caagcgttatccggaatgattgggcgtaaagcgtctgtaggtggctttccaagtcaactgtcaaaaatcagggcttaaccttgagccggcagttg
aaactatgaagcttgagttaggtaggggcagagggaattcctagtgtagcgg

>93b58b0ba0d326e9c8d1a81f8672c16a

tggggaatattgcacaatgggggaaaccctgatgcagcgacgccgcgtgagcgatgaagtatttcggtatgtaaagctctatcagcagggaag
aaaatgacggtacctgactaagaagcaccggctaaatacgtgccagcagccgcggtaatacgtatggtgcaagcgttatccggatttactgggt
gtaaagggagcgtagacggagtggcaagtctgatgtgaaaacccggggctcaaccccgggactgcattggaaactgtcaatctagagtaccgg
agaggtaagcggaattcctagtgtagcggtgaaatgcgtagatattaggaggaacaccagtggcgaaggcggcttactggacggtaactgacg
ttgaggctcgaaagcgtggggagcaaaca

>93f07800045d5772842b7590ae86af64

tagggaatcttccgcaatggacgaaagtctgacggagcaacgccgcgtgtatgaagaaggttttcggatcgtaaagtactgttgttagagaaga
acaaggataagagtaactgcttgtcccttgacggtatctaaccagaaagccacggctaactacgtgccagcagccgcggtaatacgtaggtggc
aagcgttgtccggatttattgggcgtaaagcgcgcgcaggcggtcttttaagtctgatgtgaaagcccccggcttaaccggggagggtcattgga
aactggaagactggagtgcagaagaggagagtggaattccacgtgtagcagtgaaatgcgtagagatgtggaggaacaccgatggcgaagg
cagccccctgggcctgtactgacgctcatgcacgaaagcgtggggagcaaaca

>947491f1482ef2840f9fc28c5277d655

tggggaattttccgcaatgggcgaaagcctgacggagcaataccgcgtgagggatgaaggcttactgagttgtaaacctctttttcaaggaaga
agttctgacgttacttgaagaataagcatcggctaactccgtgccagcagccgcggtaagacggaggatgcaagtgttatccggaatcactggg
cgtaaagcgtctgcaggtggtttttaagtctgatgttaaaacttgaggctcaacctcaatccagcattggaaactgaaagactagagtatagtag
ggatagagggaatttccagtggagcggtgaaatgcgtagatattggaaagaacaccgatggcgaaagcactctattggactattactgacactc
agagacgaaagctaggggagcaaatg

>94b06f09456f242293cf812d43f6c8da

tagggaatcttccgcaatggacgaaagtctgacggagcaacgccgcgtgtatgaagaaggttttcggatcgtaaagtactgttgttagagaaga
acaaggataagagtaactgcttgtcccttgacggtatctaaccagaaagccacggctaactacgtgccagcagccgcggtaatacggaggggg
ttagcgttgttcggaattactgggcgtaaagcgcacgtaggcggattggaaagttagaggtgaaatcccggggctcaaccccggaactgccttta
aaactcccagtctagagttcgagagaggtaagtggaattccgagtgtagaggtgaaattcgtagatattcggaggaacaccagtggcgaaggc
ggcttactggctcgatactgacgctgaggtgcgaaagtgtggggagcaaaca

>94b59a29370ce50fd7dc6b92027eac2c

tagggaatcttccgcaatggacgaaagtctgacggagcaacgccgcgtgtatgaagaaggttttcggatcgtaaagtactgttgttagagaaga
acaaggataagagtaactgcttgtcccttgacggtatctaaccagaaagccacggctaactacgtgccagcagccgcggtaatacgtaggtggc
aagcgttgtccggatttattgggcgtaaagcgcgcgcaggcggtcttttaagtctgatgtgaaagcccccggcttaaccggggagggtcattgga
aactggaagactggagtgcagaagaggagagtggaattccacgtgtagcggtgaaatgcgtagatatgtggaggaacaccagtgg

>94d62eef2e4702fb308c32bc470c4a93

tagggaattctggacaatgggcgaaagcttgatccggtgatatcagagaagtgaagaaggttaactccgtaaaacttgattactaaagaggatg
atgacgttatttagtaaggaagccccggctaatcttgtgccagcagccgcggtaatacaggaggggctagtgttactcatcatgattgggcgtaa
agagtatgtaggcggtttgtgaagttgatgaaagtcaaaaccagcaaactagaatataggagaggatagccgtattcctagtggagaggtgaa
atgtgaagatattaggaggacgaacaaaggcgaaggcagctatctggcttataattgacgctgagatacgaaagcacgggtagcgaata

>94f6f537930dcabb1c681113760ca0d4

tcgagaatctttcacaatgggggcaaccctgatggagcgacgccgcgtggaggataaggtcttcggattgtaaactcctgtcatgcgagaacaa
ggtagttgggttaataacctagctgcttgatggtaccgcaagaggaagagacggctaactctgtgccagcagccgcggtaatacagaggtctca
agcgttgttcggaatcactgggcgtaaagggtgcgtaggctgcgccgtaagtcagatgtgaaagctcagggctcaaccctgaaattgcatccga
tactgcggtgctagagaaatgaaggggagtctagaattttcggtgtagcagtgaaatgcgtagatatcgaaaggaataccaaaggcgaaggca
ggactctgggcattttctgacgctcatgcacgaaggctagggtagcgaacg

>952a9acbce2aaead15a571890f76c9c0

tggggaatcttggacaatgggcgaaagcccgatccagcaatatcgcgtgagtgaagaagggcaatgccgcttgtaaagctctttcgtcgagtgc
gcgatcatgacaggactcgaggaagaagccccggctaactccgtgccagcagccgcggtaagacggggggggcaagtgttcttcggaatgact
gggcgtaaagggcacgtaggcggtgaatcgggttgaaagtgaaagtcgccaaaaagtggcggaatgctctcgaaaccaattcacttgagtgag
acagaggagggtggaatttcgtgtgtaggggtgaaatccgtagatctacgaaggaacgccaaaagcgaaggcagctctctgggtccctaccga
cgctggggtgcgaaagcatggggagcgaaca

>9558a489a409072daa2651abd7ef72bc

tggggaatattgcgcaatgggcgaaagcctgacgcagccacgccgcgtgagcgatgaaggccttcgggtcgtaaagctctgtggggagagacg
aagaaagcccgtgaagagcgggccttgacggtatctccttagcaagcaccggctaactccgtgccagcagccgcggtaatacggagggtgcaa
acgttgctcggaatcattgggcgtaaagcgcacgtaggcggcgaagcaagttgggtgtgaaagccctgggctcaacccaggaagtgcattcaa
aactgcttcgcttgaatctcggagggggtcagagaattcccggtgtagaggtgaaattcgtagatatcgggaggaataccagtggcgaaggcgc
tgacctggacgaagattgacgctgaggtgcgaaagcgcggggagcaaaca

>957ab590811dc97ca515a107e4cb1e88

tagggaatcttccgcaatgggcgaaagcctgacggagcaacgccgcgtgagtgatgaaggtcttcggatcgtaaaactctgttatcagggaaga
acaaacgtgtaagtaactgtgcacgtcttgacggtacctgatcagaaagccacggctaactacgtgccagcagccgcggtaatacgtaggtggc
aagcgttatccggaattattgggcgtaaagcgcgcgtaggcggtttttaagtctgatgtgaaagcccacggctcaaccgtggagggtcattgga
aactggaaaacttgagtgcagaagaggaaagtggaattccatgtgtagcggtgaaatgcgcagagatatggaggaacaccagtggcgaaggc
gactttctggtctgtaactgacgctgatgtgcgaaagcgtggggatcaaaca

>9598d2588ce7fdb88b62b7ee13936b19

tagggaatcttccacaatggacgaaagtctgatggagcaacgccgcgtgagtgaagaaggttttcggatcgtaaaactctgttgttggagaaga
atgtatctgatagtaactgatcaggtagtgacggtatccaaccagaaagccacggctaactacgtgccagcagccgcggtaatacgtaggtggc
aagcgttgtccggatttattgggcgtaaagcgagcgcaggcggtttcttaagtctgatgtgaaagccttcggctcaaccgaagaagtgcatcgga
aactgggaaacttgagtgcagaagaggacagtggaactccatgtgtagcggtgaaatgcgtagatatatggaagaacaccagtggcgaaggc
ggctgtctggtctgtaactgacgctgaggctcgaaagcatgggtagcaaaca

>97d7b22c16e90083752975349ca1df39

tggggaattttccgcaatgggcgaaagcctgacggagcaatgccgcgtggaggtggaaggcctacgggtcgtcaacttcttttctcggagaaga
aacaatgacggtatctgaggaataagcatcggctaactctgtgccagcagccgcggtaagacagaggatgcaagcgttatccggaatgattgg
gcgtaaagcgtctgtaggtggcttttcaagtccgccgtcaaatcccagggctcaaccctggacaggcggtggaaactaccaagctggagtacgg
taggggcagagggaatttccggtggagcggtgaaatgcattgagatcggaaagaacaccaacggcgaaagcactctgctgggccgacactga
cactgagagacgaaagctaggggagcaaatgggattagataccccctgtag

>98eb46039335ec8a290cf3f5ea0fb4e5

taaggaatattggtcaatgcccggaagggtgaaccagccatgccgcgtgaaggatgactgccctctgggttgtaaacttcttttggatgggacga
aaaaacgggatttatcctggactgacggtaccatcagaataagcaccggctaactccgtgccagcagccgcggtaatacggagggtgcaagcg
ttatccggaatcactgggtttaaagggtgcgtaggcggttttataagtcagaggtgaaagcatgcagcttaactgtataattgcctttgatactgta
agacttgaataaggttgaggttggcggaatgtgacatgtagcggtgaaatgcttagatatgtcatagaacaccgattgcgaaggcagctgactg
ggcctttattgacgctgaggcacgaaagcgtggggagcgaaca

>993a18382137e354f9d889e09a47238c

tggggaatcttggacaatgggcgaaagcccgatccagcaatatcgcgtgagtgaagaagggcaatgccgcttgtaaagctctttcgtcgagtgc
gcgatcatgacaggactcgaggaagaagccccggctaactccgtgccagcagccgcggtaagacggggggggcaagtgttcttcggaatgact
gggcgtaaagggcacgtaggcggtgaatcgggttgaaagtgaaagtcgccaaaaagtggcggaatgctctcgaaaccaattcacttgagtgag
acagaggagagtggaatttcgtgtgtaggggtgaaatccgtagatctacgaaggaacgccaaaagcgaaggcagctctctgggtccctaccga
cgctggggtgcgaaagcatggggagcgaacaggattagataccccctgtag

>9960b20e17a6ebd5bf31b249c3503956

tggggaatattgcacaatgggcgaaagcctgatgcagcaacgccgcgtgagcgatgaaggccttcgggtcgtaaagctctgtcctcaaggaag
ataatgacggtacttgaggaggaagccccggctaactacgtgccagcagccgcggtaatacgtagggggctagcgttatccggatttactgggc
gtaaagggtgcgtaggtggtttcttaagtcaggagtgaaaggctacggctcaaccgtagtaagctcttgaaactgggaaacttgagtgcaggag
aggaaagtggaattcctagtgtagcggtgaaatgcgtagatattaggaggaacaccagtagcgaaggcggctttctggactgtaactgacactg
aggcacgaaagcgtggggagcaaaca

>9962d3a861210082d9b216dc6bdc8b7c

tagggaatcttccgcaatggacgaaagtctgacggagcaacgccgcgtgtatgaagaaggttttcggatcgtaaagtactgttgttagagaaga
acaaggataagagtaactgcttgtcccttgacggtatctaaccagaaagccacggctaactacgtgccagcagccgcggtaatacggagggtgc
aagcgttatccggaatcactgggtttaaagggtgcgtaggcggttagttaagtcagaggtgaaagcccggcgctcaacgtcggaattgcctttga
tactggctaacttgaatcaggttgaggtgggcggaatgtggcatgtagcggtgaaatgcatagatatgccatagaacaccaattgcgaaggcag
ctcactggccctgcattgacgctgaggcacgaaagcgtggggagcgaaca

>9a2d021f88b77d7e51b50d3718b02276

tggggaattttccgcaatgggcgaaagcctgacggagcaatgccgcgtggaggtagaaggcccacgggtcatgaacttcttttcccggagaaga
agcaatgacggtatctggggaataagcatcggctaactctgtgccagcagccgcggtaatacagaggatgcaagcgttatccggaatgattggg
cgtaaagcgtctgtaggtggctttttaagtccgccgtcaaatcccagggctcaactctggacaggcggtggaaactaccaagctggagtacggta
ggggcagagggaatttccggtggagcggtgaaatgcgtagagatcggaaaggacaccaacggcgaaagcactctgctgggccgacaccgaca
ctgagagacgaaagctaggggagcgaatg

>9a71eb30b24bad698cc5082bb184be62

tggggaatcttggacaatgggcgaaagcccgatccagcaatatcgcgtgagtgaagaagggcaatgccgcttgtaaagctctttcgtcgagtgc
gcgatcatgacaggactcgaggaagaagccccggctaactccgtgccagcagccgcggtaagacggggggggcaagtgttcttcggaatgact
gggcgtaaagggcacgtaggcggtgaatcgggttgaaagtgaaagtcgccaaaaagtggcggaatgctctcgaaaccaattcacttgagtgag
acagaggagagtggaatttcgtgtgtaggggtgaaatccgtagatctacgaaggaacgccaaaagcgaaggcagctctctgggtccctaccga
cgctggggtgcgaaagcatggggagcgaacaggattagataccctagtag

>9a7c75b7b0897efe49682881232a5f65

tgaggaattttccgcaatgggcgaaagcctgacggagcaataccgcgtggaggatgacggcctgtgggtcttaaactccttttcttagagaaga
agttctgacggtatctaaggaataagcatcggctaactccgtgccagcagccgcggtaatacgggggatgcaagcgttatccggaatgattggg
cgtaaagcgtctgtaggtggctttccaagtcaactgtcaaaaatcagggcttaaccttgagccggcagttgaaactatgaagcttgagttaggta
ggggcagagggaattcctagtgtagcggtgaaatgcgtagatattaggaagaacaccaatcgcgaaagcactctgctggccatgactgacac
t

>9ae4a24f8afe2d11c1c619180d04e01d

tggggaatcttagacaatgggggaaaccctgatctagccatgccgcgtgagcgatgaaggccttagggttgtaaagctctttcagctgggaaga
taatgacggtaccagcagaagaagccccggctaactccgtgccagcagccgcggtaatacggagggggctagcgttgttcggaattactgggc
gtaaagcgcacgtaggcggattggaaagtcagaggtgaaatcccagggctcaaccctggaactgcctttgaaactcccagtcttgaggtcgaga

gaggtgagtggaattccgagtgtagaggtgaaattcgtagatattcggaggaacaccagtggcgaaggcggctcactggctcgatactgacgct gaggtgcgaaagcgtggggagcaaaca

>9b23053de8f4269fe6b5ce286dfbef3c

tagggaatcttccacaatggacgcaagtctgatggagcaacgccgcgtgagtgaagaagggtttcggctcgtaaagctctgttggtagtgaaga aagatagaggtagtaactggcctttatttgacggtaattacttagaaagtcacggctaactacgtgccagcagccgcggtaatacgtaggtggca agcgttgtccggatttattgggcgtaaagcgagtgcaggcggttcaataagtctgatgtgaaagccttcggctcaaccggagaattgcatcagaa actgttgaacttgagtgcagaagaggagagtggaactccatgtgtagcggtggaatgcgtagatatatggaagaacaccagtggcgaaggcgg ctctctggtctgcaactgacgctgaggctcgaaagcatgggtagcgaaca

>9d3e6fb754f8ac99ef9e38a3a06ab096

tgaggaattttccgcaatgggcgaaagcctgacggagcaataccgcgtggaggatgacggcctgtgggtcttaaactccttttcttagagaaga agttctgacggtatctaaggaataagcatcggctaactccgtgccagcagccgcggtaatacgggggatgcaagcgttatccggaatgattggg cgtaaagcgtctgtaggtggctttccaagtcaactgtcaaaaatcagggcttaaccttgagccggcagttgaaactatgaagcttgagttaggta ggggcagagggaattcctagtgtagcggtgaaatgcgtagagatctgaaggaataccagtggcgaaggcggcccccctggacagatactgaca ctcagatgcgaaagcgtggggagcaaaca

>9e399cfde32835d6f62f43c10a994e4f

tggggaatattgcacaatgggcgaaagcctgatgcagcaacgccgcgtgagggatgacggccttcgggttgtaaacctctttcagtagggaaga agcgaaagtgacggtacctacagaagaagcaccggctaactacgtgccagcagccgcggtaatacgtagggtgcgagcgttgtccggaattat tgggcgtaaagagcttgtaggcggtttgtcgcgtctgctgtgaaaatccggggctcaaccccggacttgcagtgggtacgggcagactagagtgt ggtaggggagactggaattcctggtgtagcggtgaaatgcgcagatatcaggaggaacaccgatggcgaaggcaggtctctgggccactactg acgctgagaagcgaaagcgtggggagcgaaca

>9e4038cdef3bfb536a41fad896736e8f

tgaggaatattgggcaatggaggcaactctgacccagccatgccgcgtgcaggaagacggccctatgggttgtaaactgcttttatacaggaag aaacactgctacgtgtagcagcttgacggtactgtaagaataagcaccggctaactccgtgccagcagccgcggtaatacggagggtgcaagc gttatccggaatgattgggcgtaaagcgtctgtaggtggctttttaagttctccgtcaaatcccagggctcaaccctggacaggcggtgaaaacta ccaagctagagtacggtaggggcagagggaatttccggtggagcgatgaaatgcgtagagatcggaaggaacaccaacggcgaaagcactct gctgggccgacactgacactgagagacgaaagctaggggagcaaatg

>9e56fae721b91205d3a17eec65e96d0b

taaggaatattggtcaatggaggaaactctgaaccagccatcccgcgtgaaggatgaaggggctctgccttgtaaacttcttttgaatgggacga aaaaaggggatttatcctcgactgacggtaccattagaataagcaccggctaactccgtgccagcagccgcggtaatacggagggtgcaagcg ttatccggaatcactgggtttaaagggtgcgtaggcggctaaataagtcagtggtgaaaggccgtggcttaaccatgggattgccattgatactg tatagcttgaatgaggatgaggttggcggaatgtggcatgtagcggtgaaatgcttagatatgccatggaacatcgattgcgaaggcagctgac tggacctgaattgacgctgaggcacgaaagcgtgggtagcgaaca

>9e7125a102db8951720ec539b4e78113

tagggaatcttcggcaatgggggcaaccctgaccgagcaacgccgcgtgagtgaagaaggttttcggatcgtaaagctctgttgtaagagaaga acgtgtgtgagagtggaaagttcacacagtgacggtaacttaccagaaagggacggctaactacgtgccagcagccgcggtaatacgtaggtc ccgagcgttgtccggatttattgggcgtaaagcgagcgcaggcggtttaataagtctgaagttaaaggcagtggcttaaccattgttcgctttgga aactgttagacttgagtgcagaaggggagagtggaattccatgtgtagcggtgaaatgcgtagatatatggaggaacaccggtggcgaaagcg gctctctggtctgtaactgacgctgaggctcgaaagcgtggggagcaaaca

>9e8dc6c089b4d88a00f76870e272757d

tggcgaattttccgcaatgggcgaaagcctgacggagcaatgccgcgtggaggtggaaggcctacgggtcgtcaacttcttttctcggagaaga
aacaatgacggtatctgaggaataagcatcggctaactctgtgccagcagccgcggtaagacagaggatgcaagcgttatccggaatgattgg
gcgtaaagcgtctgtaggtggcttttcaagtccgccgtcaaatcccagggctcaaccctggacaggcggtggaaactaccaagctggagtacgg
taggggcagagggaatttccggtggagcggtgaaatgcattgagatcggaaagaacaccaacggcgaaagcactctgctgggccgacactga
cactgagagacgaaagctaggggagcaaatg

>9f48b0700ca4f9bc731f652161ca7bce

tggggaattttccgcaatgggcgaaagcctgacggagcaatgccgcgtggaggtagaggcccctgggtcatgaacttcttttcccggagaagaa
aaaatgacggtatccggggaataagcatcggctaactctgtgccagcagccgcggtaagacagaggatgcaagcgttatccggaatgattggg
cgtaaagcgtctgtaggtggctttttaagttcgctgtcaaataccagggctcaaccctggacaggtggtgaaaactactaagctagagtacggta
ggggcagagggaatttccggtggagcgatgaaatgcgtagagatcggaaggaacaccaacggcgaaagcactctgctgggccgacactgaca
ctgagagacgaaagctaggggagcgaatg

>9f5a3d67e86993d3c9b0e0135701c2da

tggggaattttccgcaatgggcgaaagcctgacggagcaatgccgcgtggaggtagaggcctctgggtcatgaacttcttttcccggagaagaa
aaaatgacggtatccggggaataagcatcggctaactctgtgccagcagccgcggtaagacagaggatgcaagcgttatccggaatgattggg
cgtaaagcgtctgtaggtggctttttaagttcgctgtcaaataccagggctcaaccctggacaggtggtgaaaactactaagctagagtacggta
ggggcagagggaatttccggtggagcgatgaaatgcgtagagatcggaaggaacaccaacggcgaaagcactctgctgggccgacactgaca
c

>9fa34e9fe72a259ec79dfa297ded4eb7

tgaggaattttctgcaatgggcgaaagcctgacagagcaataccgcgtgagggatgacggcctgcgggttgtaaacctcttttctcaagaaaga
agatctgacgctacttgaggaataagcatcggctaactctgtgccagcagccgcggtaatacagaggatgcaagtgttattcggaattattgggc
gtaaagcgtctgtaggtgggcaattaagtcttttgtcaaatctttgggcttaacctaaagcgtgcaaaagaaactgtttgcctagagtctgttaga
ggtaaagggaatttccagtggagcggtgaaatgcgtagatattggaaggaacaccaaaggcgaaagcactttactgggtcagtactgacactg
agagacgaaagctaggggagcaaaca

>9fc5e219349ec9655d8b7c7cca60749e

tggggaattttggacaatgggggcaaccctgatccagccattccgcgtgagtgaagaaggccttcgggttgtaaagctctttcagcaggaacga
aacggtggtccctaatacgggctactaatgacggtacctgaagaagaagcaccggctaactacgtgccagcagccgcggtaatacgtagggtg
cgagcgttaatcggaattactgggcgtaaagcgtgcgcaggcggttatataagacagatgtgaaatccccgggcttaacctgggaactgcgtttg
tgactgtataacttgagtgtggcagagggggggtggaattccacgtgtagcagtgaaatgcgtagagatgtggaggaacaccgatggcgaaggc
agccccctgggtcaacactgacgctcatgcacgaaagcgtggggagcaaaca

>a07d3c42790d498fbbdd1f1d8269bbb5

tggggaatattggacaatgggcgcaagcctgatccagccatgccgcgtgagtgatgaaggccctagggttgtaaagctctttcaccggtgaaga
taatgacggtaaccggagaagaagccccggctaacttcgtgccagcagccgcggtaatacgaagggggctagcgttgttcggaattactgggc
gtaaagcgcacgtaggcgggtatttaagtcaggggtgaaatcccagagctcaactctggaactgcctttgatactgggtacctagagtatggaa
gaggtaagtggaattccgagtgtagaggtgaaattcgtagatattcggaggaacaccagtggcgaaggcggcttactggtccattactgacgct
gaggtgcgaaagcgtggggagcaaacaggattagataccctggtag

>a09a81e81834c8edb6d987bba585854d

tggggaattttccgcaatgggcgaaagcctgacggagcaatgccgcgtggaggtagaagtcccacgggtcatgaacttcttttcccggagaaga
agcaatgacggtatctggggaataagcatcggctaactctgtgccagcagccgcggtaatacagaggatgcaagcgttatccggaatgattggg
cgtaaagcgtctgtaggtggctttttaagtccgccgtcaaatcccagggctcaactctggacaggcggtggaaactaccaagctggagtacggta
ggggcagagggaatttccggtggagcggtgaaatgcgtagagatcggaaagaacaccaacggcgaaagcactctgctgggccgacactgaca
ctgagagacgaaagctaggggagcgaatg

>a0d41403222d849db9df4541729f6d54

tgaggaatattggacaacgtatgaaaatatgatccagcaatattgtagaggtgaagaggtaaacttgcagagcaggtaggcttttaaaacctta
gttttaatgaagatagtgacgttaattaaaatattagtcctgactaatttcgtgccagcagtcgcggtgatacggagaggacgagtgttacttgta
ttgactgggtgtaaagcgtatgtagacgtctaaaaaagtttagatttaaaaatttagttataactaaaaaatttctttttattttttagattgagttat
aaggaagattacagaattttaagtgttaggggtaaaatccgtttgaaatttaaaagactatcaattggcgaaggcagtaatctatttataactga
cgttgagatacagaagcataggtatcgaaca

>a1a24e3152c07f68044dc58b61884e70

tggggaattttggacaatgggcgaaagcctgatccagcaatgccgcgtgagtgaagaaggccttcgggttgtaaagctcttttgtcagggaaga
aacggtgggagctaatatctcctgctaatgacggtacctgaagaataagcaccggctaactacgtgccagcagccgcggtaatacgtagggtgc
aagcgttaatcggaattactgggcgtaaagcgtgcgcaggcggttttgtaagtctgtcgtgaaatccccgggctcaacctgggaattgcgatgga
gactgcaaggctagaatctggcagagggggggtagaattccacgtgtagcagtgaaatgcgtagagatgtggaggaacaccgatggcgaaggc
agccccctgggtcaagattgacgctcatgcacgaaagcgtggggagcaaaca

>a48196c73921763a9f66211d2e6e1390

tggggaatcttgcgcaatgggcggaagcctgacgcagcgacgccgcgtgggggatgaaggccttcgggttgtaaacctctttcagcagggaag
aagccgtgaggtgacggtacctgcagaagaagcaccggctaactacgtgccagcagccgcggtaatacgtagggtgcgagcgttgtccggaat
tattgggcgtaaagagctcgtaggtggcgggtcacgtcgggtgtgaaagcccggggcttaaccccgggtctgcattcgatacgggcttgctaggg
tccggcaggggagactggaattcctggtgtagcggtgaaatgcgcagatatcaggaggaacaccggtggcgaaggcgggtctctgggccggta
ccgacgctgaggagcgaaagcgtggggagcgaaca

>a4c2f12c09ff1bdea81d8528ebe741b6

tggggaattttgcgcaatgggggaaaccctgacgcagcaatgccgcgtggaggacgaaggtcttcggattgtaaactccttttgcgggggacaa
atagcggcgtgaataacgccgactgatggtactccgtgaataagccacggctaacttcgtgccagcagccgcggtaagacgaaggtggcgagc
gttactcggaattactaggcgtaaagcgtgggcaggcggtttggtaagtctgctgtgaaagccccgggctcaacccggggaggtcagtggatac
tgccggacttggatgtgggagaggtgactggaattcctggtgtagcggtgaaatgcgtagatatcaggaggaacaccgatggcgaaagcaggt
cactggaccactatcgacgctcatccacgaaagctgggggatcaaaca

>a627f4fe57afccc972a77351fbfb42f8

tggggaatattccgcaatgggcgaaagcctgacggagcaatgccgcgtggaggtagaaggcctacgggtcatgaactttttttcccggagaaga
aaaaatgacggtatccggggaataagcatcggctaactctgtgccagcagccgcggtaagacagaggatgcaagcgttatccggaatgattgg
gcgtaaagcgtctgtaggtggctttttaagttctccgtcaaatcccagggctcaaccctggacaggcggtgaaaactaccaagctagagtacggt
aggggcagagggaatttccggtggagcgatgaaatgcgtagagatcggaaggaacaccaacggcgaaagcactctgctgggccgacactgac
actgagagacgaaagctaggggagcaaatg

>a822eed692bc6f11f3e7a953ac0a23e4

tagggaatcttccacaatggacgcaagtctgatggagcaacgccgcgtgagtgaagaaggtcttcggatcgtaaaactctgttgttagagaaga
acacgagtgagagtaactgttcattcgatgacggtatctaaccagcaagtcacggctaactacgtgccagcagccgcggtaatacgtaggtggc
aagcgttgtccggatttattgggcgtaaagggaacgcaggcggtcttttaagtctgatgtgaaagccttcggcttaaccggagtagtgcattgga
aactggaagacttgagtgcagaagaggagagtggaactccatgtgtagcggtgaaatgcgtagatatatggaagaacaccagtggcgaaagc
ggctctctggtctgtaactgacgctgaggttcgaaagcgtgggtagcaaaca

>a86329980f132594bc3933a7b6ffee4c

tggggaatattggacaatgggcgaaagcctgatccagcaacgccgcgtgagggatgacggccttcgggttgtaaacctctttcagtagggacga
agcgcaagtgacggtacctacagaagaagcaccggccaactacgtgccagcagccgcggtaatacgtagggtgcgagcgttgtccggaattat
tgggcgtaaagggctcgtaggcggtttgtcacgtcgggagtgaaatctcagggctcaaccctgagcctgcttccgatacgggcagactagaggt

atgcaggggagaacggaattcctggtgtagcggtgaaatgcgcagatatcaggaggaacaccggtggcgaaggcggttctctgggcattacct
gacgctgaggagcgaaagtgtggggagcgaaca

>a87a08f34176a01d56939af85dce7384

tggggaatattgcacaatgggcgaaagcctgatgcagcgacgccgcgtgagggatgaaggccttcgggttgtaaacctctttcagcagggaag
aagcgaaagtgacggtacctgcagaagaagcaccggctaactacgtgccagcagccgcggtaatacgtagggtgcgagcgttgtccggaatta
ttgggcgtaaagagcttgtaggcggtttgtcgcgtctgcagtgaaaatccggggctcaaccccggacttgctgtgggtacgggcagactagagta
tggtaggggagaccggaattcctggtgtagcggtggaatgcgcagatatcaggaggaacaccgatggcgaaggcaggtctctgggccataact
gacgctgagaagcgaaagcatggggagcgaaca

>a9e028a7a419d1c9fd55dcc73423fdf1

tggggaattttggacaatgggcgcaagcctgatccagcaatgccgcgtgcaggaagaaggccttcgggttgtaaactgcttttgtacggagcga
aaaggctctctctaatacaggggggttcatgacggtaccgtaagaataagcaccggctaactacgtgccagcagccgcggtaatacgtagggtgc
gagcgttaatcggaattactgggcgtaaagcgtgcgcaggcggttttgtaagacaggcgtgaaatccccgggctcaacctgggaatggcgcttg
tgactgcaaagctggagtgcggcagaggggggatggaattccgcgtgtagcagtgaaatgcgtagatatgcggaggaacaccgatggcgaagg
caatcccctgggcctgcactgacgctcatgcacgaaagcgtggggagcaaaca

>aa70d7ca71967603d5a9a82c6bf5b9bf

tagggaatattgggcaatggatgcaagtctgacccagccatgccgcgtggaggaagaaggtcctctggattgtaaactccttttgtcggggaag
aataggcaggatgcgtcctgttgtgacggtacccgatgaataagccacggctaactacgtgccagcagccgcggtaatacgtaggtggcaagc
gttgtccggatttattgggtttaaagggtgcgtaggtggtttattaagtcagtggtgaaagctggttgctcaacaatcaagttgccattgatactga
tagacttgagagaagtggaggttcatggaatggatggtgtagcggtgaaatgcatagatatcatccagaacgtcgattgcgaaggcagtgggct
gtactttttctgacactgaggcacgaaagcgtggggatcaaaca

>ab8a067bee46678530028fbc97f80daf

tggggaatattggacaatgggcgcaagcctgatccagccatgccgcgtgagtgatgaaggccttagggttgtaaagctcttttgtccgggaagat
aatgactgtaccggaagaataagccccggctaacttcgtgccagcagccgcggtaatacgaagggggctagcgttgttcggaatcactgggcgt
aaagcgcacgtaggcggacttttaagtcagaggtgaaagcctggagctcaactccagaactgcctttgatactgggagtctcgagttcggaaga
ggttggtggaactgcgagtgtagaggtgaaattcgtagatattcgcaagaacaccagtggcgaaggcggccaactggtccgatactgacgctga
ggtgcgaaagcgtggggagcaaaca

>ac340b3d0a0c7cc4688f5bfbfc91c670

tagggaatcttccgcaatggacgaaagtctgacggagcaacgccgcgtgtatgaagaaggttttcggatcgtaaagtactgttgttagagaaga
acaaggataagagtaactgcttgtcccttgacggtatctaaccagaaagccacggctaactccgtgccagcagccgcggtaatacggagggtgc
aagcgttaatcggaattactgggcgtaaagcgcgcgtaggtggtttgataagcgagatgtgaaagccccgggcttaacctgggaacggcatttc
gaactgtcaggctagagtatggtagaggagtgtggaatttcctgtgtagcggtgaaatgcgtagatataggaaggaacaccagtggcgaaggc
ggcactctggaccaatactgacactgaggtgcgaaagcgtggggagcaaaca

>ac8195c3decdfd4fdf2113d253050c4b

tggggaatattggacaatgggcgcaagcctgatccagccatgccgcgtgtgtgaagaaggccttttggttgtaaagcactttaagcgaggagga
ggcttacctggttaatacctgggataagtggacgttactcgcagaataagcaccggctaactctgtgccagcagccgcggtaatacagagggtg
cgagcgttaatcggaattactgggcgtaaagcgcgcgtaggcggtttgttaagtcggatgtgaaatccccgggctcaacctgggaattgcattcg
atactggcaagctagagtatgggagaggaaggtagaattccaggtgtagcggtgaaatgcgtagagatctggaggaataccgatggcgaagg
cagccttctggcctaatactgacgctgaggtgcgaaagcatggggagcaaaca

>ad0978ef3bee6a173bb66d94dfab1054

tggggaatcttagacaatgggggaaaccctgatctagccatgccgcgtgagcgatgaaggccttagggttgtaaagctctttcgtgggggaaga
taatgactgtaccccaagaagaagccccggctaactccgtgccagcagccgcggtaatacggagggggctagcgttgttcggaattactgggcg
taaagcgcacgtaggcggaccagaaagtcagaggtgaaagcccagggctcaaccttggaactgcctttgaaactcctggtcttgaggtcgaga
gaggtgagtggaattccgagtgtagaggtgaaattcgtagatattcggaggaacaccagtggcgaaggcggctcactggctcgatactgacgct
gaggtgcgaaagcgtggggagcaaaca

>ad7b12e3042568621340521446cc187f

tagggaatcttccgcaatggacgaaagtctgacggagcaacgccgcgtgtatgaagaaggttttcggatcgtaaagtactgttgttagagaaga
acaaggataagagtaactgcttgtcccttgacggtatctaaccagaaagccacggctaactacgtgccagcagccgcggtaatacgtaggtggc
aagcgttgtccggatttattgggtttaaagggtgcgtaggcgggcctttaagtcagtggtgaaagcctgcagcttaactgtagaactgccattgat
actggaggccttgagtgtactagaggtaggcggaatttatggtgtagcggtgaaatgcatagataccataaagaacaccgatagcgtaggcag
cttactggagtacaactgacgctgaggcacgaaagcatggggagcgaaca

>ad89a9d5fec4408fafb73e7225f44d08

tggggaatattgcacaatggaggaaactctgatgcagcgacgccgcgtgaaggatgaagtatttcggtatgtaaacttctatcagcagggaaga
aaatgacggtacctgactaagaagccccggctaactacgtgccagcagccgcggtaatacgtagggggcaagcgttatccggatttactgggtg
taaagggagcgtagacggcacggcaagccagatgtgaaagcccggggctcaaccccgggactgcatttggaactgctgagctagagtgtcgga
gaggcaagtggaattcctagtgtagcggtgaaatgcgtagatattaggaggaacaccagtggcgaaggcggcttgctggacgatgactgacgtt
gaggctcgaaagcgtggggagcaaaca

>ae760d84b7424a463b2e75dc32ffe09e

tgaggaatattggacaacgtatgaaaatatgatccagcaatattgtagaggtgaagaggtaaacttgcagagcaggtaggcttttaaaacctta
gttttaatgaagatagtgacgttaattaaaatattagtcctgactaatttcgtgccagcagtcgcggtgatacggagaggacgagtgttacttgta
ttgactgggtgtaaagcgtatgtagacgtctaaaaaagtttagatttaaaaatttagttataactaaaaaatttctttttattttttagattgagctat
aaggaagattacagaattttaagtgttaggggtaaaatccgtttgaaatttaaaagactatcaattggcgaaggcagtaatctatttataactga
cgttgagatacagaagcataggtatcgaaca

>af0ef250af55d02406fed5ed336a5a9a

tggggaattttccgcaatgggcgaaagcctgacggagcaatgccgcgtggaggtggaaggcctacgggtcgtcaacttcttttctcggagaaga
aacaatgacggtatctgaggaataagcatcggctaactctgtgccagcagccgcggtaagacagaggatgcaagcgttatccggaatgattgg
gcgtaaagcgtctgtaggtggcttttcaagtccgccgtcaaatcccagggctcaaccctggacaggcggtggaaactaccaagctggagtacgg
taggggcagagggaatttccggtggagcggtgaaatgcattgagatcggaaagaacaccaacggcgaaagcactctgctgggccgacactga
cactgagagacgaaagctaggggagcaaatgggattagataccccagtagt

>affbb89176c9aba1631f271d6c3aea03

tggggaatctcggacaatgggcaaaagcccgatccagcaatatcgcgtgagtgaagaagggcaatgccgcttgtaaagctctttcgtcgagtgc
gcgatcatgacaggactcgaggaagaagcccgggctaactctgtgccggcagccgcggtaatacgggggggggggcaagtgttcttcggaatg
actgggcgtaaagggcacgtaggtggtgaattgggttggaagcgaaagtcgccaaaaagtggccgaatgctctcgaaaccaattcacttgagt
gagacagaggatagtggaatttcgtgtgtaggggtgaaatccgtagatctacgaaggaacaccaaaagcgaaggcagctctctgggtccatac
cgatgctgggttgcgaaagcatggggagcaaaca

>b00f448acaa36e64a82e7cef6a94b985

tggggaatattgcacaatgggcgaaagcctgatgcagcgacgccgcgtgagggatgaaggccttcgggttgtaaacctctttcagtagggaaga
agcgcaagtgacggtacctacagaagaagcaccggctaactacgtgccagcagccgcggtaatacgtagggtgcgagcgttgtccggaattat
tgggcgtaaagagcttgtaggcggtttgtcgcgtctgccgtgaaaatccggggctcaactccggacttgcggtgggtacgggcagactagagtgt
ggtaggggagactggaattcctggtgtagcggtgaaatgcgcagatatcaggaggaacaccgatggcgaaggcaggtctctgggccactactg
acgctgagaagcgaaagcgtggggagcgaaca

>b02a8d33d018119dedb2db15db887bfd

tggggaatattgcacaatgggcggaagcctgatgcagcaacgccgcgtgcgggatgacggccttcgggttgtaaaccgctttcgcctgtgacga
agcgtgagtgacggtaatgggtaaagaagcaccggctaactacgtgccagcagccgcggtgatacgtagggtgcgagcgttgtccggatttatt
gggcgtaaagggctcgtaggtggttgatcgcgtcggaagtgtaatcttggggcttaaccctgagcgtgctttcgatacgggttgacttgaggaag
gtaggggagaatggaattcctggtggagcggtggaatgcgcagatatcaggaggaacaccagtggcgaaggcggttctctgggcctttcctgac
gctgaggagcgaaagcgtggggagcgaaca

>b2cee3e4eaa128047bfa241265b991b9

tggggaatcttggacaatgggcgaaagcccgatccagcaatatcgcgtgagtgaagaagggcaatgccgcttgtaaagctctttcgtcgagtgc
gcgatcatgacaggactcgaggaagaagccccggctaactccgtgccagcagccgcggtaagatgggggcaagtgttcttcggaatgactgg
gcgtaaagggcacgtaggcggtgaatcgggttgaaagtgaaagtcgccaaaaagtggcggaatgctctcgaaaccaattcacttgagtgagac
agaggagagtggaatttcatgtgtaggggtgaaatccgtagatctacgaaggaacgccaaaagcgaaggcagctctctgggtccctaccgacg
ctggggtgcgaaagcatgggagcgaaca

>b315cb5141ac07819043e9fad145d6b2

tagggaatcttgcgcaatgggcgaaagcctgacgcagcaatgccgcgtgcgggacgaaggccctagggtcgtaaaccgctttcagtagggacg
aaaatgacggtacctgcagaagaagctccggccaactacgtgccagcagccgcggtgatacgtagggagcaagcgttgtccggaattactggg
cgtaaagggctcgtaggtggttgagtaagtcagatgtgaaatctcagggcccaaccctgagcctgcatttgatactgctctgactagagtccggt
aggggagtgcggaactcctggtgtagcggtgaaatgcgcagatatcaggaagaacaccgacagcgaaggcagcactctgggccggtactgac
actgaggagcgaaagcatgggtagcaaaca

>b350a52b82312cc2209425da69e892a6

tggggaattttccgcaatgggcgaaagcctgacggagcaatgccgcgtgaaggtagaaggcctacgggtcatgaacttcttttcccggagaaga
agcaatgacggtatccggggaataagcatcggctaactctgtgccagcagccgcggtaagacagaggatgcaagcgttatccggaatgattgg
gcgtaaagcgtctgtaggtggctttttaagttcgccgtcaaatcccagggctcaaccctggacaggcggtggaaactaccaagctggagtacggt
aggggcagagggaatttccggtggaacggtgaaatgcgtagagatcggaaagaacaccaacggcgaaagcactctgctgggccgacactgac
actgagagacgaaagctaggggagcgaatg

>b39a827ea8ca43b654d9adfeefc5b275

tagggaatcttccacaatggacgaaagtctgatggagcaatgccgcgtgcaggatgaatgccttcgggttgtaaactgcttttatgggggagga
atatgacagtaccccatgaataagcttcggctaactccgtgccagcagccgcggtcatacggaggaagcaagcgttatccggaattattgggcg
taaagagatgcgtaggtggttcaccaagcaaagcatgaaatcatgaggctcaacctcatgactgtgctttgaactgatgaacttgagcatgata
gaggtaactggaattccttgtgtaggagtgaaatccgtagatataaggaggaacaccaatggcgaaggcaggttactggatcattgctgacact
gaggctcgaaagcgtggggagcgaacg

>b3ecd38f3d8df213c2127f8e68d3d294

ttggaaacgactgctaattccctatatgctgaggagtaaaacggtattaaccgcctaaagatgagcttgcgtctgattagctagttggtaaggtaa
tggcttaccaaggcgatgatcagtagctggtctgagaggatgatcagccacactgggactgagacacggcccagactcctacgggaggcagca
gtgaggaattttccgcaatgggcgaaagcctgacggagcaataccgcgtggaggatgacggcctgtgggtcttaaactccttttcttagagaaga
agttctgacggtatctaaggaataagcatcggctaactccgtgccagcagccgcggtaatacgggggatgcaagcgttatccggaatgattggg
cgtaaagcgtctgtaggtggctttccaagtcaactgtcaaaaatcagggcttaaccttgagcc

>b4a04b44d15eec86f0042a9cf38c5c38

tagggaatcttccgcaatggacgaaagtctgacggagcaacgccgcgtgtatgaagaaggttttcggatcgtaaagtactgttgttagagaaga
acaaggataagagtaactgcttgtcccttgacggtatctaaccagaaagccacggctaactacgtgccagcagccgcggtaatacgtaggtggc
aagcgttgtccggatttattgggcgtaaagcgcgcgcaggcggtcctttaagtctgatgtgaaatcttgcggctcaaccgcaagcggtcattgga

aactgggggacttgagtgcagaagaggaaagcggaattccacgtgtagcggtgaaatgcgtagagatgtggaggaacaccagtggcgaaggc
ggctttctggtctgtaactgacgctgaggcgcgaaagcgtggggagcaaaca

>b4ed8ec9d3313b1ae0b929812511444b

tggggaatattgcacaatgggggaaaccctgatgcagcaacgccgcgtgagtgatgacggccttcgggttgtaaagctctgtcttcagggacga
taatgacggtacctgaggaggaagccacggctaactacgtgccagcagccgcggtaatacgtaggtggcgagcgttgtccggatttactgggcg
taaagggagcgtaggcggatttttaagtgagatgtgaaatactcgggcttaacctgagtgctgcatttcaaactggaagtctagagtgcaggag
aggagaagggaattcctagtgtagcggtgaaatgcgtagagattaggaagaacaccagtggcgaaggcgcttctctggactgtaactgacgct
gaggctcgaaagcgtggggagcaaaca

>b5253c2c72c07b32a04a07299e586619

tagggaattctggacaatgggcgcaagcttgatccggtgatatcagagaagtgaagaaggttaactccgtaaaacttgattactaaagaggatg
atgacgttatttagtaaggaagccccggctaatcttgtgccagcagccgcggtaatacaggaggggctagtgttactcatcatgattgggcgtaa
agagtatgtaggcggtttgtgaagttgatgaaagtcaaaaccagcaaactagaatataggagaggatagccgtattcctagtggagaggtgaa
atgtgaagatattaggaggacgaacaaaggcgaaggcagctatctggcttataattgacgctgagatacgaaagcacgggtagcgaata

>b56f6a907150e6e385a19b1f280594c6

tggggaatattggacaatgggcgcaagcctgatccagccatgccgcgtgtgtgaagaaggccttttggttgtaaagcactttaagcgaggagga
ggcttacctggttaatacctgggataagtggacgttactcgcagaataagcaccggctaactctgtgccagcagccgcggtaatacagagggtg
cgagcgttaatcggaattactgggcgtaaagcgcgcgtaggcggtttgttaagtcggatgtgaaatccccgggctcaacctgggaattgcattcg
atactggcaagctagagtatgggagaggaaggtagaattccaggtgtagcggtgaaatgcgtagagatctggaggaataccgatggcgaagg
cagccttctggcctaatactgacgctgaggtgcgaaagcatggggagcaaacaggattagatacccgggtag

>b65beee66f71dd29d3334f00a165fa02

tggggaatattggacaatgggcgaaagcctgatccagcaatatcgcgtgagtgaagaaggctattttggttgtaaagctctttcgtcgaggaaga
taatgacggtactcgaagaagaagccccggctaactccgtgccagcagccgcggtaagacggagggggctagtgttattcggaatgactgggc
gtaaagggcacgtaggcggtgaattaagttgaaggtgaaatcccaaagcttaactttggaactgttttcaaaactgattcactggagttcgatag
gggatagtggaatttctagtgtagaaataaaattcataaatattagaaggaacaccagtggcgaaggcgactatctggatcgatactgacgctg
aggtgcgaaagcatggggagcaaaca

>b6f3e929dc0a0c2baec219f6bf4a0ab6

tagggaatcttccacaatggacgcaagtctgatggagcaacgccgcgtgagtgaagaagggtttcggctcgtaaagctctgttggtagtgaaga
aagatagaggtagtaactggcctttatttgacggtaattacttagaaagtcacggctaactacgtgccagcagccgcggtaatacgtaggtggca
agcgttgtccggatttattgggcgtaaagcgagtgcaggcggttcaataagtctgatgtgaaagccttcggctcaaccggagaattgcatcagaa
actgttgaacttgagtgcaggagaggagagtggaactccatgtgtagcggtggaatgcgtagatatatggaagaacaccagtggcgaaggcgg
ctctctggtctgcaactgacgctgaggctcgaaagcatgggtagcgaaca

>b7424fffb8b9c2200ef0862f93ed72a0

tggggaatcttggacaatgggcgaaagcccgatccagtaatatcgcatgagtgaagaagggcaatgccgcttgtaaagctctttcgtcgagtgc
gcgatcataacaggactcgaggaagaagccccagctaactccgtgccagcagccgcggtaagacggggggggggggaagtgttcttcggaatg
actgggcgtaaagggcacgtaggcggtgaatcgggttgaaagtgaaagtcgccaaaaagtggcggaatgctcttgaaaccaattcacttgagt
gagacagaggagagtggaattttgtgtgtaggggtgaaatccgtagatctacgaaggaacgccaaaagcgaatgcagctctctgggtccctac
cgacgctggggtgcgaaagcatggggagcgaaca

>b77b151f7481fd080bedbf415e736539

tagggaatcttccgcaatggacgaaagtctgacggagcaacgccgcgtgtatgaagaaggttttcggatcgtaaagtactgttgttagagaaga
acaaggataagagtaactgcttgtcccttgacggtatctaaccagaaagccacggctaactacgtgccagcagccgcggtaatacgtaggtggc

aagcgttgtccggatttattgggcgtaaagcgcgcgcaggcggtcttttaagtctgatgtgaaagcccccggcttaaccggggagggtcattgga
aactggaagactggagtgcagaagaggagagtggaattccacgtgtagcggtgaaatgcgtagatatgtggaggaacaccagtggcgaaggc
gactctctggtctgtaactgacgctgaggcgcgaaagcgtggggagcaaaca

>b7ae493f11862e60da4e2200e532c7d2

tgaggaatattggtcaatgggcgagagcctgaaccagccaagtagcgtgcaggaagacggccctatgggttgtaaactgcttttgtgcgggaat
aaagcgggtcacgtgtggccctttgcatgtaccgtacgaataaggaccggctaattccgtgccagcagccgcggtaatacggaaggtccgggcg
ttatccggatttattgggtttaaagggagcgtaggccgtctcaaaagcgtgttgtgaaatgtccgggctcaaccggggccgtgcagcgcgaactg
tgagacttgagtgtgcggaaggtatgcggaattcgtggtgtagcggtgaaatgcatagatatcacgaagaactccgattgcgaaggcagcatgc
cgcagcattactgacgctgatgctcgaaggtgcgggtatcgaaca

>b885ce70b6fc10105bb5433a7f64009c

tggggaatattgcacaatgggggaaaccctgatgcagccatgccgcgtgtgtgaagaaggccttcgggttgtaaagcactttcagcgaggagga
aaggttgatacctaatacgtatcagctgtgacgttactcgcagaagaagcaccggctaactccgtgccagcagccgcggtaatacggagggtgc
aagcgttaatcggaattactgggcgtaaagcgcgcgtaggcggcttgttaagccagatgtgaaagccccgggcttaacctgggaactgcatttg
gaactggcaggctagagtacagaagagggtggtggaatttcctgtgtagcggtgaaatgcgtagatataggaaggaacatcagtggcgaaggc
ggccacctggtctgatactgacgctgaggtgcgaaagcgtggggagcaaaca

>b8cf7d81ea474eb84e3f294bd9d80f18

taaggaatattggacaatggagggaactctgatccagccattccgcgtggaggaagaatgccctatgggttgtaaacttcttttggatgggaata
aaatgccccttgcgaggggaactgaaggtaccatcagaataagcaccggctaactccgtgccagcagccgcggtaatacggagggtgcgagcg
ttatccggaattactgggtttaaagggtgcgtaggcggaaatttaagtctggggtgaaatattgtggcttaaccataagattgccttggatactgg
gtttcttgaattgggatgaggatggcggaatgtgacatgtagcggtgaaatgcatagatatgtcatagaacaccaattgcgaaggcagctatctg
gtctcatattgacgctgaggcacgaaagcgtggggagcgaaca

>b92e8e3439bb67597cb6567326e109c9

tgaggaatattggacaatgggcgggagcctgatccagccatgccgcgtgcaggaagacggccctatgggttgtaaactgcttttgcacgggaag
aaacacccctcgcgagggggcctgacggtaccgtgagaataaggatcggctaactccgtgccagcagccgcggtaatacggaggatccgagc
gttatccggaatcattgggtttaaagggtccgtaggtggacggttaagtcaggggtgaaatcccgcagctcaactgtggaattgcccttgatactg
gtcgtcttgagtcgttgtgaagtggttggaatatgtagtgtagcggtgaaatgcatagatattacatagaacaccgattgcgaaggcagaccact
aacgacgtactgacactgatggacgaaagcgtggggagcgaaca

>b96ea3fbd5dff31db3519d849c438acf

tagggaatcttccgcaatggacgaaagtctgacggagcaacgccgcgtgtatgaagaaggttttcggatcgtaaagtactgttgttagagaaga
acaaggataagagtaactgcttgtcccttgacggtatctaaccagaaagccacggctaactacgtgccagcagccgcggtaatacgtaggtggc
aagcgttgtccggatttattgggcgtaaagcgcgcgcaggcggtcttttaagtctgatgtgaaagcccccggcttaaccggggagggtcattgga
aactggaagactggagtgcagaagaggagagtggaattccgagtgtagaggtgaaattcgtagatattcggaggaacaccagtggcgaaggc
ggctcactggctcgatactgacgctgaggtgcgaaagtgtggggagcaaaca

>b9cc3361a2632915ea0bffd368b4a3d6

tggggaatattgcacaatgggcgaaagcctgatgcagccatgccgcgtgtgtgatgaaggccttagggttgtaaaacactttcatcggtgaagat
aatgacggtagccgaagaagaagccccggctaactccgtgccagcagccgcggtaatacggagggggcaagcgttgttcggaattactgggcg
taaagcgcgcgtaggcggactggtcagttgggggtgaaatcccgaggcttaacctcggaactgccttcaatactgccagtcttgagatcggaag
aggtgagtggaattcctagtgtagaggtgaaattcatagatattaggaagaacaccagtggcgaaggcggctcactggtccgatactgacgctg
aggtgcgaaagcgtggggagcgaaca

>b9d614025bf292dfb0db9f43d7357e14

tgaggaatattggacaatgggtgagagcctgatccagccatcccgcgtgaaggacgacggccctatgggttgtaaacttcttttgtatagggata
aacctactctcgtgagagtagctgaaggtactatacgaataagcaccggctaactccgtgccagcagccgcggtaatacggagggtgcaagcgt
tatccggatttattgggtttaaagggtccgtaggcggatctgtaagtcagtggtgaaatctcacagcttaactgtgaaactgccattgatactgca
ggtcttgagtgttgttgaagtagctggaataagtagtgtagcggtgaaatgcatagatattacttagaacaccaattgcgaaggcaggttactaa
gcaacaactgacgctgatggacgaaagcgtggggagcgaaca

>b9edf27fc18aeed5186fafda3a4ef895

tagggaattttccacaatgggcgaaagcctgatggagcaacgccgcgtgcaggatgaaggccctcgggtcgtaaactgcttttatatgtgacga
atatgacggtagcatatgaataaggatcggctaactccgtgccagcagccgcggtcatacggaggatccaagcgttatccggaattactgggcg
taaagagttgcgtaggtggcatagtatgtctgtagtgaaagcgtgtggctcaaccatatacacattacagaaactgctaagctagagggcgaga
gaggtaactagaattcctagtgtaggagtggaatccgtagatattaggaggaataccgatggcgtaggcaggttactggctcgtccctgacacta
aggcacgaaagcgtggggagcaaacg

>ba18621ad8282f2a59949d728a39e949

tggggaattttccgcaatgggcgaaagcctgacggagcaatgccgcgtggaggtagaggcccctgggtcatgaacttcttttcccggagaagaa
aaaatgacggtatccggggaataagcatcggctaactctgtgccagcagccgcggtaagacagaggatgcaagcgttatccggaatgattggg
cgtaaagcgtctgtaggtggctttttaagttcgctgtcaaataccagggctcaaccctggacaggtggtgaaaactactaagctagagtacggta
ggggcagagggaatttccggtggagcgatgaaatgcgtagagatcggaaggaacaccaacggcgaaagcactctgctgggccgacactgaca
ctgagagacgaaagctaggggagcgaatgggattagatacccctgtagt

>ba30115f693ccfea9103a1b04d08c0c2

taaggaatattggacaatggacgaaagtctgatccagccatgccgcgtgaaggattaaggtcctctggattgtaaacttcttttatatgggacga
aaaaagggcattcttgttcacttgacggtaccatatgaataagcaccggctaactccgtgccagcagccgcggtaatacggagggtgcaagcgt
tatccggattcactgggtttaaagggtgcgtaggcgggtagataagtcagtggtgaaatctcggagcttaactccgaaactgccattgatactatt
tatcttgaatattgtggaggtttgcggaatatgtcatgtagcggtgaaatgcttagatatgacatagaacacctattgcgaaggcagcaggctac
gcatatattgacgctgaggcacgaaagcgtggggatcaaaca

>ba36086e9292cf0adc3cc339fe964f1d

tggggaattttccgcaatgggcgaaagcctgacggagcaatgccgcgtggaggtagaaggcctacgggtcctgaacttcttttcccagagaaga
agcaatgacggtatctggggaataagcatcggctaactctgtgccagcagccgcggtaatacagaggatgcaagcgttatccggaatgattggg
cgtaaagcgtctgtaggtggctttttaagtccgccgtcaaatcccagggctcaaccctggacaggcggtggaaactaccaagcttgagtacggta
ggggcagagggaatttccggtggagcggtgaaatgcgtagagatcggaaagaacaccaacggcgaaagcactctgctgggccgacactgaca
ctgagagacgaaagctaggggagcgaatg

>bac3db3ffb89bae882811e6139a4861a

tggggaattttccgcaatgggcgaaagcctgacggagcaatgccgcgtggaggtagaaggcccacgggtcgtgaacttcttttcccggagaaga
agcaatgacggtatctgaggaataagcatcggctaactctgtgccagcagccgcggtaagacagaggatgcaagcgttatctggaatgattgg
gcgtaaggcgtctgtaggtggcttttcaagtccgccgtccaatcccagggctcaaccctggacaggcggtggaaactaccaagctggagtacgg
taggggcagagggaatttccggtggagcggtgaaatgcgtagagatcggaaagaacaccaacggcgaaagcaccctgctgggccgacactga
cactgagagacgaaagctaggggagcgaatgggattagataccccagta

>bcf1ff7652c847af1c4e33b471a8ae4d

tagggaatcttccacaatggacgcaagtctgatggagcaacgccgcgtgagtgaagaaggttttcggatcgtaaagctctgttgttggtgaaga
aggatagaggtagtaactggcctttatttgacggtaatcaaccagaaagtcacggctaactacgtgccagcagccgcggtaatacgcaggtggc
aagcgttgtccggatttattgggcgtaaagcgagcgcaggcggaagaataagtctgatgtgaaagccctcggcttaaccgaggaactgcatcgg
aaactgtttttcttgagtgcagaagaggagagtggaactccatgtgtagcggtggaatgcgtagatatatggaagaacaccagtggcgaaggcg
gctctctggtctgcaactgacgctgaggctcgaaagcatgggtagcgaaca

>bd627be18e8b3b0e6f31fc13897d2d0d

tgaggaatattgggcaatggacgcaagtctgacccagcaacgccgcgtggaggatgaaggtctttggattgtaaactccttttgcaggggaaga
gtgaggacggtaccctgagaataagtcacggctaactacgtgccagcagccgcggtaatacgtaggtgacgagcgttatccggatttattgggc
gtaaagcgcacgcaggcggcttgacaagtctgacgtgaaatctcccggcttaactgggagcggtcgttggaaactgtcgagcttgaggcgatga
gaggggtgtggaattcccggtgtagtggtggaatgcgtagatatcgggggaacatcagtggcgaaagcggcaccctggcattagcctgacgct
catgtgcgaaagcgtggggagcgaacg

>bd79f81fc2537c2a4203a8cac6a44c3c

tggggaattttccgcaatgggcgaaagcctgacggagcaatgccgcgtggaggtagaaggcccacgggtcatgaacttcttttcccggagaaga
agcaatgacggtatctggggaataagcatcggctaactctgtgccagcagccgcggtaatacagaggatgcaagcgttatccggaatgattggg
cgtaaagcgtctgtaggtggctttttaagtccgccgtcaaatcccagggctcaactctggacaggcggtggaaactaccaagctggagtacggta
ggggcagagggaatttccggtggagcggtgaaatgcgtagagatcggaaagaacaccaacggcgaaagcactctgctgggccgacactgaca
ctgagagacgaaagctaggggagcgaatgggattagataccccagtag

>be0979510db98021cb356a50f8b03f5b

tggggaatattgggcaatggacgaaagtctgacccagcaacgccgcgtgggggatgaaggccttagggttgtaaactccttttattagggaaga
tagtgacggtacctaatgaataagcatcggctaacttcgtgccagcagccgcggtaatacgaaggatgctagcgttactcggaattactgggcgt
aaagaatgcgtaggcgggcatttaagttgatagtgcaatccccaagcttaacttaggaattgctatcaaaactaaatgccttgaatatgaaaggg
agcaagggaattcctggtgtaggggtaaaatccgtagatatcaggaggaagaccggaagcgaaagcgcttgcttggttcattattgacgctgag
gcatgaaagcgtggggatcgaaca

>be31ea1de761f3757a78eb2fed4ef9b3

tggggaattttccgcaatgggcgaaagcctgacggagcaatgccgcgtggaggtagaaggcccacgggtcatgaacttcttttcccggagaaga
agcaatgacggtatctggggaataagcatcggctaactctgtgccagcagccgcggtaatacagaggatgcaagcgttatccggaatgattggg
cgtaaagcgtctgtaggtggctttttaagtccgccgtcaaatcccagggctcaactctggacaggcggtggaaactatcaagctggagtacggta
ggggcagagggaatttccggtggagcggtgaaatgcgtagagatcggaaagaacaccaacggcgaaagcactctgctgggccgacactgaca
ctgagagacgaaagctaggggagcgaatg

>bf2da96267afcee99ff82053cd18af28

tggggaatattggacaatgggcgcaagcctgatccagccataccgcgtgggtgaagaaggccttcgggttgtaaagcccttttgttgggaaaga
aaagcagtcggctaatacccgattgttctgacggtacccaaagaataagcaccggctaacttcgtgccagcagccgcggtaatacgaagggtgc
aagcgttactcggaattactgggcgtaaagcgtgcgtaggtggtttgttaagtctgctgtgaaagccctgggctcaacctgggaattgcagtggat
actggctgactagaatgtggcagagggtagcggaattcctggtgtagcagtgaaatgcgtagagatcaggaggaacatccgtggcgaaggcgg
ctacctgggccaacattgacactgaggcacgaaagcgtggggagcaaaca

>bf369c9bcb5d821045ed2cb045eb99ff

tgggggatattgcacaatgggcgcaagcctgatgcagcgacgccgcgtgagggatgacggctttcgggttgtaaacctctttcgctaccgaaca
aggtcccatattggtggggttgagggtaggtggataagaagcgccggctaactacgtgccagcagccgcggtaatacgtagggcgcgagcgtt
gtccggaattattgggcgtaaagagctcgtaggcggcttgtcgcgtctgctgtgaaaacgcggggcttaactccgcgcgtgcagtgggtacgggc
aggctagagtgtggtaggggtgactggaattcctggtgtagcggtggaatgcgcagatatcaggaggaacaccgatggcgaaggcaggtcact
gggccattactgacgctgaggagcgaaagcatggggagcgaaca

>c01af9824c4da0da698e80bdda2f9f7e

tggggaatattgcacaatgggggaaaccctgatgcagccatgccgcgtgtgtgaagaaggccttcgggttgtaaagcactttcagcgaggagga
aaggttagtagttaataactgctagctgtgacgttactcgcagaagaaggaccggctaactccgtgccagcagccgcggtaatacgggggatgc
aagcgttatccggaatgattgggcgtaaagcgtctgtaggtggctttttaagtccgccgtcaaatcccagggctcaactctggacaggcggtgga

aactaccaagctggagtacggtaggggcagagggaatttccggtggagcggtgaaatgcgtagagatcggaaagaacaccaacggcgaaag
cactctgctgggccgacactgacactgagagacgaaagctaggggagcgaatg

>c05eb66ecce9bbb304ac580f4d29fdf2

tagggaattttccgcaatgggcgaaagcctgacggagcaataccgcgtgagggatgacggcctatgggttgtaaacctctttttcagggagga
ataaatgacgtgtacctgaagaataagcatcggctaactccgtgccagcagccgcggtaagacggaggatgcaagtgttatccggaatcactg
ggcgtaaagcgtctgtaggtggtcaaataagtcaactgttaaatcttgaggctcaacctcaaaaccgcagtcgaaactattagactagagtatag
taggggtaaagggaatttccagtggagcggtgaaatgcgtagagattggaaggaacaccgatggcgaaggcactttactgggctattactaac
actcagagacgaaagctagggtagcaaatg

>c0975f7ca5988524fffc584723730284

tggggaattttccgcaatgggcgaaagcctgacggagcaatgccgcgtggaggtggaaggcctacgggtcgtcaacttcttttctcggagaaga
aacaatgacggtatctgaggaataagcatcggctaactctgtgccagcagccgcggtaagacagaggatgcaagcgttatccggaatgattgg
gcgtaaagcgtctgtaggtggcttttcaagtccgccgtcaaatcccagggctcaaccctggacaggcggtggaaactaccaagctggagtacgg
taggggcagagggaatttccggtggagcggtgaaatgcattgagatcggaaagaacaccaacggcgaaagcactctgctgggccgacactga
cactgagagacgaaagctaggggagcaaatg

>c0c1191d62430ccb0ca9d73a686a0896

taaggaatattggtcaatggagggaactctgaaccagccatcccgcgtgcaggatgaaggggctctgccctgtaaactgcttttgaatgggacg
aaaaagcctcacttgtgaggaactgacggtaccattagaataagcaccggctaactccgtgccagcagccgcggtaatacggagggtgcaagc
gttatccggaatcactgggtttaaagggtgcgtaggcggttgtataagtcagtggtgaaaggccgtagcttaactatgggattgccattgatactg
tatgacttgaatgaggttgaggttggcggaatgtggcatgtagcggtgaaatgcttagatatgccatggaacatcgattgcgaaggcagctgact
ggacctgaattgacgctgaggcacgaaagcgtggggagcgaaca

>c0c787f2760226e8a202506f0f0a65a1

tggggaattttccgcaatgggcgaaagcctgacggagcgacaccgcgtggaggatgaaatttttcggaatgtaaactcctttttggcaggaatga
aacaaatgacagtacctgcagaataagcatcggctaactacgtgccagcagccgcggtaatacgtaggatgcaagcgttgttcggaattactgg
gcgtaaagagtacgtaggcggtaatgaaagtctgatattaaagactggggcttaacctcaggagtgtatcggaaacttcataactagaggacag
tagaggaaattggaattcccagtgtagcggtgaaatgcgtagatattgggaggaacaccagtggcgaaagcgaatttctgggctgttactgacg
ctgaggtacgaaagtgtggggagcaaaca

>c1a0da0199a33d0c570b5e086c8ebc8a

tggggaattttccgcaatgggcgaaagcctgacggagcaatgccgtgtggaggtggaaggcctacgggtcgtcaacttcttttctcggagaaga
aacaatgacggtatctgaggaataagcatcggctaactctgtgccagcagccgcggtaagacagaggatgcaagcgttatccggaatgattgg
gcgtaaagcgtctgtaggtggcttttcaagtccgccgtcaaatcccagggctcaaccctggacaggcggtggaaactaccaagctggagtacgg
taggggcagagggaatttccggtggagcggtgaaatgcattgagatcggaaagaacaccaacggcgaaagcactctgctgggccgacactga
cactgagagacgaaagctaggggagcaaatg

>c3b6ad1498373be0fca2a0eb8eed482d

tgaggaattttccgcaatgggcgaaagcctgacggagcaataccgcgtgaaggatgaaggcctatgggttgtaaacttcttttctcagagaaga
attttgacggtatctgaggaataagcatcggctaactctgtgccagcagccgcggtaagacagaggatgcaagcgttatccggattaattgggc
gtaaagcgtctgtaggtggtttatcaagtcttttgtcaaagatcagggcctaaccctgggcaggcagaagaaactaatagacttgagttcggtag
ggcagagggaattcccggtggagcggtgaaatgcgtagagatcgggaggaacaccaaaggcgaaggcactctgctgggccgtaactgacac
tgagagacgaaagcgagggtagcaaatg

>c4e200f2a1ad9a8651964946dccf41c9

tagggaattttccacaatgggcgaaagcctgatggagcaacgccgcgtgcaggatgaatgccttcgggttgtaaactgcttttattagtgaagaa
tatgacggtaactaatgaataagggtcggctaactacgtgccagcagccgcggtcatacgtaggacccaagcgttatccggaattactgggcgt
aaagagttgcgtaggtggcatgataagtggagtgtgaaagcgtgcggctcaaccgcatatccgcattccaaactattaagcttgagtatgagag
aggtagatggaatttctagtgtaggagtgaaatccgtagatattagaaggaacaccgatggcgtaggcagtctactggctcattactgacactaa
ggcacgaaagcgtggggagcgaaca

>c50cc6d44589d46a41a1ea21be2db653

tggggaatattggacaatgggcgcaagcctgatccagccatgccgcgtgtgtgaagaaggccttcgggttgtaaagcacttttgttggggaaga
aaccgaccggattaatacttcggttgaatgacggtacccaaagaataagcaccggctaacttcgtgccagcagccgcggtaatacgaagggtgc
aagcgttactcggaattactgggcgtaaagcgtgcgtaggcggtttgttaagtctgtcgtgaaagccccgggctcaacctgggaatggcgatgga
tactggcaagctagagtgtgtcagaggatggtggaattcccggtgtagcggtgaaatgcgtagagatcgggaggaacaccagtggcgaaggcg
gccatctgggacaacactgacgctgaggcacgaaagcgtggggagcaaaca

>c70dfc304c4f13c14c44e516b8165dbc

tgaggaatattggacaatggacgcaagtctgatccagccatgccgcgtgcaggaagaatgccctatgggttgtaaactgcttttatttgggaata
aacccctctacgtgtagagggctgaaggtaccaaacgaataagcaccggctaactccgtgccagcagccgcggtaatacggagggtgcaagcg
ttatccggaatcattgggtttaaagggtccgcaggcggacgtataagtcagtggtgaaagcctacagcttaactgtagaactgccattgatactgt
acgtcttgaattcggtcgaagtgggcggaatgtgtcatgtagcggtgaaatgcatagatatgacacagaacaccgatagcgaaggcagctcact
aggcctgaattgacgctcagggacgaaagcgtggggagcgaaca

>c7db868cd7f9b9b7cf55b2fa9bb51e7f

tggggaattttccgcaatgggcgaaagcctgacggagcaatgccgcgtgaaggtagaaggcctacgggtcatgaacttcttttcccggagaaga
agcaatgacggtatccggggaataagcatcggctaactctgtgccagcagccgcggtaagacagaggatgcaagcgttatccggaatgattgg
gcgtaaagcgtctgtaggtggctttttaagttcgccgtcaaatcccagggctcaaccctggacaggcggtggaaactaccaagctggagtacggt
aggggcagagggaatttccggtggagcggtgaaatgcgtagagatcggaaagaacaccaacggcgaaagcactctgctgggccgacactgac
actgagagacgaaagctaggggagcgaatg

>c80ffca7f4952e3e9f8d0e1b3b405702

cgaggaattttccgcaatgggcgaaagcctgacggagcaataccgcgtggaggatgacggcctgtgggtcttaaactccttttcttagagaaga
agttctgacggtatctaaggaataagcatcggctaactccgtgccagcagccgcggtaatacgggggatgcaagcgttatccggaatgattggg
cgtaaagcgtctgtaggtggctttccaagtcaactgtcaaaaatcagggcttaaccttgagccggcagttgaaactatgaagcttgagttaggta
ggggcagagggaattcctagtgtagcggtgaaatgcgtagatattaggaagaacaccaatcgcgaaagcactctgctgggccatgactgacac
tgagagacgaaagctaggggagcgaatg

>c8101d4116a4b2ca40592e2f0ca8d845

tagggaatcttccgcaatggacgaaagtctgacggagcaacgccgcgtgtatgaagaaggttttcggatcgtaaagtactgttgttagagaaga
acaaggataagagtaactgcttgtcccttgacggtatctaaccagaaagccacggctaactacgtgccagcagccgcggtaatacgtaggtggc
aagcgttgtccggatttattgggcgtaaagcgcgcgcaggcggtcttttaagtctgatgtgaaagcccccggcttaaccggggagggtcattgga
aactggaagactggagtgcagaagaggagagtggaactccatgtgtagcggtggaatgcgtagatatatggaagaacaccagtggcgaaggc
ggctctctggtctgcaactgacgctgaggctcgaaagcatgggtagcgaaca

>c84530ba77f10f52ddc0d7636863cc48

tagggaatcttccgcaatggacgaaagtctgacggagcaacgccgcgtgtatgaagaaggcctgcgggttgtaaagcacttttagtggggagg
aaggccgccgacctaatacgtcggaggattgacttaacccacagaaaaagcaccggctaactctgtgccagcagccgcggtaatacagagggt
gcgagcgttaatcggaattactgggcgtaaagcgtgcgtaggcggtttcgtaagtcgggtgtgaaagccccgggctcaacctgggaattgcattc
gagactgcggagctggagtgcggaagagggaagtggaatttccggtgtagcggtgaaatgcgtagatatcggaaggaacatcagtggcgaaa
gcgacttcctggtccagcactgacgctcaggcacgaaagcgtggggagcaaaca

>c8501fa5cc4855dfe465623dc2c73870

tggggaattttccgcaatgggcgaaagcctgacggagcaatgccgcgtggaggtggaaggcctacgggtcgtcaacttcttttctcggagaaga
aacaatgacggtacctgaggaataagcatcggctaactctgtgccagcagccgcggtaagacagaggatgcaagcgttatccggaatgattgg
gcgtaaagcgtctgtaggtggcttttcaagtccgccgtcaaatcccagggctcaaccctggacaggcggtggaaactaccaagctggagtacgg
taggggcagagggaatttccggtggagcggtgaaatgcattgagatcggaaagaacaccaacggcgaaagcactctgctgggccgacactga
cactgagagacgaaagctaggggagcaaatg

>caf9ced72cd2c9f89c4e5cde99148906

tggggaattttccgcaatgggcgaaagcctgacggagcaatgccgcgtggaggtagaaggcccacgggtcgtgaacttcttttcccggagaaga
agcaatgacggtatctgaggaataagcatcggctaactctgtgccagcagccgcggtaagacagaggatgcaagcgttatctggaatgattgg
gcgtaaggcgtctgtaggtggcttttcaagtccgccgtccaatcccagggctcaaccctggacaggcggtggaaactaccaagctggagtacgg
taggggcagagggaatttccggtggagcggtgaaatgcgtagagatcggaaagaacaccaacggcgaaagcaccctgctgggccgacactga
cactgagagacgaaagctaggggagcgaatgggattagatacccgagta

>cb3a451b5f9718c07b491a77b14ef21f

tggggaatattgcacaatggggggaaaccctgatgcagccatgccgcgtgtgtgaagaaggccttcgggttgtaaagcactttcagcgaggagga
aaggttgatacctaatacgtatcagctgtgacgttactcgcagaagaagcaccggctaactccgtgccagcagccgcggtaatacggagggtgc
aagcgttaatcggaattactgggcgtaaagcgcacgcaggcggttggataagttagatgtgaaagccccgggctcaacctgggaattgcattta
aaactgtccagctagagtcttgtagaggggggtagaattccaggtgtagcggtgaaatgcgtagagatctggaggaataccggtggcgaaggc
ggccccctggacaaagactgacgctcaggtgcgaaagcgtggggagcaaaca

>cba8447928c66779a8044e22687ec0d4

tggggaatcttagacaatgggggaaaccctgatctagccatgccgcgtgagcgatgaaggccttagggttgtaaagctctttcagtggggaaga
taatgacggtacccacagaagaagccccggctaactccgtgccagcagccgcggtaatacggagggggctagcgttgttcggaattactgggc
gtaaagcgcacgtaggcggactggaaagtcagaggtgaaatcccagggctcaaccttggaactgcctttgaaactcctggtcttgaggtcgaga
gaggtgagtggaattccgagtgtagaggtgaaattcgtagatattcggaggaacaccagtggcgaaggcggctcactggctcgatactgacgct
gaggtgcgaaagcgtggggagcaaaca

>cd16616c1eb974160069008dab2002b0

ctaggaatattggacaatgggcgaaagcctgatccagcaacaccgcgtggaggacgaaggccttcgggtcgtaaactcctttttggggggatga
gaacggacagtacccccagaataaggcccggctaactacgtgccagcagccgcggtaatacgtaggggccaagcgttatccggatttactggg
cgtaaagcgcatgtaggcggtctactaagtcggacgtgaaagctcctggctcaactaggagaggtcgttcgaaactagtagactagagggtggt
agaggagagcggaattccaggtgtagcggtgaaatgcgtagatatctggaggaacaccagtggcgaaagcggctctctggcccactcctgacg
ctgagatgcgaaagccaggggagagaacg

>ce269bc5e6f5fb15085bb532e1c2d276

ttaggaattttgcgcaatgggggaaaccctgacgcagcaacgccgcgtgaaggatgaaggctttcgggtcgtaaacttcttttagcagggaaga
aaaaaatgacggtatctgcagaaaaagcaccggctaactctgtgccagcagccgcggtaatacagagggtgcaagcgttattcggaatcactg
ggcgtaaagagctcgtaggcggttaattaagtcgaatgtgaaagccctgggctcaatccaggaagtgcattcgatactgattgactggagtgca
ggagaggaaagcggaattccaggtgtagaggtgaaattcgtagatatctggaggaacaacagaagcgaaggcggctttctggactgtcactga
cgctgaggagcgaaagcgtggggagcaaaca

>cf2e322a70d56572cbb6f07c13572a06

caaggaattttccgcaatgggcgaaagcctgacggagcaacgccgcgtgggtgatgacgcttttcggagtgtaaagcccttttctcagggaaga
acacaatgacggtacctgaggaataaggaccggctaactacgtgccagcagccgcggtaatacgtagggtccaagcgttgtccggagttactg
ggcgtaaagcgcgcgcaggcggttcgataagactggcgtgaaagcccccggctcaaccggggagggtcgttggtgactgtcggacttgaagac

agcaggggcaggtggaattcccggtgtagtggtgaaatgcgtagagatcgggaggaacacccgtggcgaaggcggcctgctgggctgttcttg
acgctgaggcgcgaaggcgtggggagcgaacg

>cf327843fd016333cfe51889752981a9

tggggaattttggacaatgggcgaaagcctgatccagcaatgccgcgtgcaggacgaaggccttcgggttgtaaactgcttttgtacggaacga
aacggtctcttctaatacaaggggctaatgacggtaccgtaagaataagcaccggctaactacgtgccagcagccgcggtaatacgtagggtgc
gagcgttaatcggaattactgggcgtaaagcgtgcgcaggcggttatataagacagatgtgaaatccccgggctcaacctgggacctgcatttgt
gactgtatagctagagtacggtagaggggggatggaattccgcgtgtagcagtgaaatgcgtagatatgcggaggaacaccgatggcgaaggca
atcccctggacctgtactgacgctcatgcacgaaagcgtggggagcaaaca

>d026ba8391312cd4726993268770b541

tggggaattttggacaatgggggcaaccctgatccagcaatgccgcgtgtgtgaagaaggccttcgggttgtaaagcacttttgtccgggaaga
aatcgcacctgataataccgggtgtggatgacggtaccggaagaataaggaccggctaactacgtgccagcagccgcggtaatacgtagggtc
caagcgttaatcggaattactgggcgtaaagcgtgcgcaggcggttgtgcaagaccgatgtgaaatccccgggcttaacctgggaattgcattg
gtgactgcacggctagagtgtgtcagaggggggtagaattccacgtgtagcagtgaaatgcgtagagatgtggaggaataccgatggcgaagg
cagccccctgggataacactgacgctcatgcacgaaagcgtggggagcaaaca

>d09be871b5a4eb743216a6382df2aa50

tagggaatcttccgcaatggacgaaagtctgacggagcaacgccgcgtgtatgaagaaggttttcggatcgtaaagtactgttgttagagaaga
acaaggataagagtaactgcttgtcccttgacggtatctaaccagaaagccacggctaactacgtgccagcagccgcggtaatacgtagggtgc
gagcgttaatcggaattactgggcgtaaagcgtgcgcaggcggtcatgcaagacagaggtgaaatccccgggctcaacctgggaactgcctttg
tgactgcatggctggagtgcggcagaggggggatggaattccgcgtgtagcagtgaaatgcgtagatatgcggaggaacaccgatggcgaagg
caatcccctgggcctgcactgacgctcatgcacgaaagcgtggggagcaaaca

>d10cb323cc534bb09847cc22d4467f75

tagggaatcttccgcaatggacgaaagtctgacggagcaacgccgcgtgtatgaagaaggttttcggatcgtaaagtactgttgttagagaaga
acaaggataagagtaactgcttgtcccttgacggtatctaaccagaaagccacggctaactacgtgccagcagccgcggtaatacgtaggtggc
aagcgttgtccggatttattgggtttaaagggtgcgtaggcggctgtttaagtcagtggtgaaagactccggctcaaccggagcagtgccattgat
actggacagcttgagtattggaggggtacatggaattgatggtgtagcggtgaaatgcatagataccatcaggaacaccgatagcgaaggcatt
gtactggccaataactgacgctgatgcacgaaagcatgggtagcgaaca

>d1e7dc710b2c81174163cc4bd364f4ec

tggggaatattttgcaatgagcgcaagcttgacaaagtatcgtcacgtgtaggaagaaggcttttataagttgtaaacttcttttgcttaaaagtg
aatattgacacttaagtataaaaagtatcggcaaactccgtgccagcagccgcggtaatacgggggatacaagcgttatccggaattactgggc
gtaaagcgtatgtaggtggttttatctatttttaattaaataaaactatataaatgttttttttaaaaatattattaaacttgagtatgttaggggtaa
aaggaattctttgagaagtggtgaaatgcgaagatacaaagaggaagaccgaaagcgaaagcattttactggagcattactgacactgagata
cgaaagttaaggtagcgaatg

>d2a847b8ff36676ce1bb763b7afa7d60

tggggaatattggacaatgggcgaaagcctgatccagcaatgccgcgtgtgtgaagaaggcccgagggttgtaaagcacttttatgagtaaag
aaaagttaaagattaatactctttaaccctgacggtaactcaggaataagcaccggctaactccgtgccagcagccgcggtaatacggagggtg
caagcgttaatcggatttactgggcgtaaagcgtccgtaggtggcttgtcaagttggatgtgaaagcccagggctcaaccttggaattgcatcca
aaactggctcgctagagtatgtgagaggggggtagaatttctggtgtagcggtgaaatgcgtagagatcagaaagaataccgatggcgaaggc
aaccccctggcgcaatactgacactgagggacgaaagcgtggggagcaaaca

>d2d2476686676334f7f06d3a89bbfcd7

tggggaattttccgcaatgggcgaaagcctgacggagcaatgccgcgtggaggtagaaggcccacgggtcgtgaacttcttttcccggagaaga
agcaatgacggtatctggggaataagcatcggctaactctgtgccagcagccgcggtaatacagaggatgcaagcgttatccggaattattggg
cgtaaagcgtctgtaggtggctttttaagtccgccgtcaaatcccagggctcaaccctggacaggcggtggaaactaccaagctggagtacggt
aggggcagagggaatttccggtggagcggtgaaatgcgtagagatcggaaagaacaccaacggcgaaagcactctgctgggccgacactgac
actgagagacgaaagctagggtagcgaatg

>d385f1d5c383b338966e78c8682f5d25

tagggaatcttccgcaatggacgaaagtctgacggagcaacgccgcgtgtatgaagaaggttttcggatcgtaaagtactgttgttagagaaga
acaaggataagagtaactgcttgtcccttgacggtatctaaccagaaagccacggctaactacgtgccagcagccgcggtaacacgaacccga
caaacgttattcggaattattgggcttaaagggtgcgtaggcggtgcggtaagtgaggtgtgaaatccctcggctcaaccgaggaattgcgcttc
atactgccgtgctggagggagacaggggtaagcggaactgatggtggagcggtgaaatgcgttgatatcatcaggaacaccggtggcgaaag
cggcttactgggtctcttctgacgctgaggcacgaaagctagggtagcgaacg

>d4a11d35318fb9ee1f6faca38687f563

tggggaatattggacaatgggcgcaagcctgatccagccatgccgcgtgagtgatgaaggccttagggttgtaaagctcttttgtccgggacgat
aatgacggtaccggaagaataagccccggctaacttcgtgccagcagccgcggtaatacgaagggggctagcgttgctcggaatcactgggcg
taaagggcgcgtaggcggccttgtaagttgggggtgaaagcccgtggctcaaccacggaattgccttcgatactgcttggcttgagtgtggtaga
ggttggtggaactgcgagtgtagaggtgaaattcgtagatattcgcaagaacaccggtggcgaaggcggccaactggaccatcactgacgctg
aggcgcgaaagcgtggggagcaaaca

>d589e65202082f7f71dcaa9c10829fdf

tggggaatcttagacaatgggggaaaccctgatctagccatgccgcgtgagtgatgaaggccttagggttgtaaagctctttcagctgggaagat
aatgacggtaccagcagaagaagccccggctaactccgtgccagcagccgcggtaatacggagggggctagcgttgttcggaattactgggcg
taaagcgcacgtaggcggactgaaaagttggaggtgaaatcccagggctcaaccttggaactgccttcaaaactatcagtctggagttcgagag
aggtgagtggaattccgagtgtagaggtgaaattcgtagatattcggaggaacaccagtggcgaaggcggctcactggctcgatactgacgctg
aggtgcgaaagcgtggggagcaaaca

>d6bc012209f57dc63c548dc423183f2f

tgaggaatattggacaatgggcgagagcctgatccagccatgccgcgtgcaggaagactgccctatgggttgtaaactgcttttatacaggaag
aaaccactttacgtgtagagtgctgacggtactgtaagaataaggatcggctaactccgtgccagcagccgcggtaatacgggggatgcaagcg
ttatccggaatgattgggcgtaaagcgtctgtaggtggctttttaagtccgccgtcaaatcccagggctcaactctggacaggcggtggaaacta
ccaagctggagtacggtaggggcagagggaatttccggtggagcggtgaaatgcgtagagatcggaaagaacaccaacggcgaaagcactct
gctgggccgacactgacactgagagacgaaagctaggggagcgaatg

>d6eb6ac8ab8ea7a5de3ced31fc51500a

tagggaatcttccacaatggacgcaagtctgatggagcaacgccgcgtgagtgaagaaggttttcggatcgtaaagctctgttgttggtgaaga
aggatagaggtagtaactggcctttatttgacggtaatcaaccagaaagtcacggctaactacgtgccagcagccgcggtaatacgtaggtggc
aagcgttgtccggatttattgggcgtaaagcgagcgcaggcggaaaaataagtctaatgtgaaagccctcggcttaaccgaggaactgcatcgg
aaactgtttttcttgagtgcagaagaggagagtggaactccatgtgtagcggtggaatgcgtagatatatggaagaacaccagtggcgaaggcg
gctctctggtctgcaactgacgctgaggctcgaaagcatgggtagcgaaca

>d744fb02a7dba0b10878ad783c980f1b

tagggaatattggacaatggacgaaagtctgatccagcaacgccgcgtgaaggatgaaggtcttaggattgtaaacttcttttgacagggagga
taatgactgtacctgtagaataagcaccggctaacttcgtgccagcagccgcggtaatacgaagggtgccagcattactcggaatcactgggcg
taaagaatgcgtaggcggacttgcaagttaggagtgaaatccctgggctcaacctaggaactgcttttaaaactgcttgtcttgaatgctagagt
gattaggggaattcttggtgtaggggtaaaatccgtagatatcaggaggaagaccggaagcgaaagcgcttaattagctagatattgacgctga
tgcatgaaagcgtggggagcaaacaggattagatacccgggtag

>d7e0bc436fc872df32bf245f73b65a28

tggggaattttccgcaatgggcgaaagcctgacggagcaatgccgcgtggaggtggaaggcctacgggtcgtcaacttcttttctcggagaaga
aacaatgacggtatctgaggaataagcatcggctaactctgtgccagcagccgcggtaagacagaggatgcaagcgttatccggaatgattgg
gcgtaaagcgtctgtaggtggcttttcaagtccaccgtcaaatcccagggctcaaccctggacaggcggtggaaactaccaagatggagtacgg
taggggcagagggaatttccggtggagcggtgaaatgcattgagatcggaaagaacaccaatggcaaaagcactctgctgggccgacactga
cactgagagacgaaagctaggggagcaaatg

>d84065c2d950637296fd8d2110047cb9

tggggaattttccgcaatgggcgaaagcctgacggagcaatgccgcgtggaggtagaaggcccacgggtcatgaacttcttttcccggagaaga
agcaatgacggtatctggggaataagcatcggctaactctgtgccagcagccgcggtaatacagaggatgcaagcgttatccggaatgattggg
cgtaaagcgtctgtaggtggctttttaagtccgccgtcaaatcccagggctcaactctggacaggcggtggaaactaccaagctggagtacggta
ggggcagagggaatttccggtggagcggtgaaatgcgtagagatcggaaagaacaccaacggcgaaagcactctgctgggccgacactgaca
ctgagagacgaaagctaggggagcgaatg

>d89562295d5f0058f04bce4ffe763344

taaggaatattggtcaatggacgaaagtctgaaccagccatgccgcgtggaggatgaaggtcctctggattgtaaacttcttttatatgggacga
aaaaaggcttttctaagtcgtctgacggtaccatatgaataagcaccggctaactccgtgccagcagccgcggtaatacggagggtgcaagcgt
tatccggattcactgggtttaaagggagcgtaggtgggctgataagtcagtggtgaaatctccgagcttaactcggaaattgccgttgatactatt
agtcttgaatattgtggaggtcagcggaatatgtcatgtagcggtgaaatgcttagatatgacatggaacaccaattgcgaaggcagctggctac
gcatatattgacactgaggctcgaaagcgtggggatcaaaca

>d907f8a6295f731a1c79e17d4d990911

tggggaattttccgcaatgggcgaaagcctgacggagcaatgccgcgtggaggtagaaggcccacgggtcatgaacttcttttcccggagaaga
agcaatgacggtatctggggaataagcatcggctaactctgtgccagcagccgcggtaatacagaggatgcaagcgttatccggaatgattggg
cgtaaagcgtctgtaggtggctttttaagtccgccgtcaaatcccagggctcaactctggacaggcggtggaaactaccaagctggagtacggta
ggggcagagggaatttccggtggagcggtgaaatgcgtagagatcggaaagaacaccaacggcgaaagcactctgctgggccgacactgaca
ctgagagacgaaagctaggggagcgaatgggattagataccccctgtagt

>d92eeff90aec306248a73713ac09b0eb

tagggaattttccgcaatgggcgaaagcctgacggagcaataccgcgtgagggatgacggcctgtgggttgtaaacctcttttctcaaggaaga
agttctgacggtacttgaggaataagcatcggctaactctgtgccagcagccgcggtaatacagaggatgcaagcgttatccggaatcactggg
cataaagcgtctgtaggttgtttagtaagtctgctgttaaagactggggctcaaccccagaaaagcagtggaaactgctagacttgagtgtggta
gaggtagagggaattcctagtgtagcggtgaaatgcgtagatattaggaagaacaccaatggcgaaggcactctactgggccataactgacac
tgagagacgacagctaggggagcaaatg

>d992d93456ed538c4098b57fded59ef3

tgaggaattttctgcaatgggcgaaagcctgacagagcaataccgcgtgagggatgaagacttactgagttgtaaacctcggtaccttaaggaa
gaagatctgacggtacttaaggtggaaagcatcggctaactccgtgccagcagccgcggtaagacgggggatgcaagtgttatccggatttact
gggcgtaaagcgtctgcaggtggttttctaagtctactgttaaatcttgaggctcaacctcaaatctgcagtagaaactagaagacttgagtatag
taggggtagagggaatttccagtggagcggtgaaatgcgtagatattggaaagaacaccgatggcgaaggcactctactgggctattactgac
actcagagacgaaagctaggggagcaaatg

>daad36a97029257a0e7d45a0e4c91723

tggggaatattgcacaatgggcgaaagcctgatgcagcaacgccgcgtgagggacgacggccttcgggttgtaaacctcttttagcagggaag
aagcgaaagtgacggtacctgcagaaaaagcgccggctaactacgtgccagcagccgcggtaatacgtagggcgcaagcgttatccggaatta
ttgggcgtaaagagctcgtaggcggtttgtcgcgtctgctgtgaaaccccgaggctcaacctcgggcctgcagtgggtacgggcagactagagt

gcggtagaggagattggaattcctggtgtagcggtggaatgcgcagatatcaggaggaacaccgatggcgaaggcagatctctgggccgtaac
tgacgctgaggagcgaaagggtggggagcaaaca

>db2a5104f2b7e6dfa1288cf38775327e

tggggaattttggacaatgggcgaaagcctgatccagcaatgccgcgtgtgtgaagaaggccttcgggttgtaaagcacttttgtccggaaaga
aatccttggctctaatacagtcgggggatgacggtaccggaagaataagcaccggctaactacgtgccagcagccgcggtaatacgtagggtg
cgagcgttaatcggaattactgggcgtaaagcgtgcgcaggcggttgtgtaagacaggtgtgaaatccccgggcttaacctgggaactgcgctt
gtgactgcacagctagagtacggcagaggggggtggaattccacgtgtagcagtgaaatgcgtagagatgtggaggaacaccgatggcgaag
gcagccccctgggcctgtactgacgctcatgcacgaaagcgtggggagcaaaca

>dbb9bc8820ba90527a8780d8b1f5fb47

tgaggaattttccgcaatgagcgaaagcttgacggagcaataccgcgtggaggacgaaggctcatgggttgtaaactccttttcttaggaaaga
attttgacggtacctaaggaataagcatcggctaattccgtgccagcagccgcggtaagacgggagatgcaagcgttattcggatttattgggcg
taaagggtctgtaggttgtcaattaagtctatggtcaaattccgaagctcaacttcgggccggcactagaaactaattgacttgagtacgttagag
gcagggggaattcccagtgtagcggtgaaatgcgtagatattgggaggaacatcaatggcgaaggcaccctgctaggccgatactgacactga
aagacgaaagctaggggagcaaatg

>dc3163df0acc6285fa82db782747565f

ccaccttacctacgggggctgcagtgaggaattttccgcaatgggcgaaagcctgacggagcaataccgcgtggaggatgacggcctgtgggt
cttaaactccttttcttagagaagaagttctgacggtatctaaggaataagcatcggctaactccgtgccagcagccgcggtaatacgggggatg
caagcgttatccggaatgattgggcgtaaagcgtctgtaggtggctttccaagtcaactgtcaaaaatcagggcttaaccttgagccggcagttg
aaactatgaagcttgagttaggtaggggcagagggaattcctagtgtagcggtgaaatgcgtagatattaggaagaacaccaatcgcgaaagc
actctgctgggccatgactgacactgagagacgaaagctaggggagcgaatg

>dd308dfe1cc4bb7b84174931ac5e39a8

tggggaattttggacaatgggggcaaccctgatccagccatgccgcgtgagtgaagaaggccttcgggttgtaaagctctttcaggtggaaaga
aatcactaccccgaatacgggtggtggatgacggtaccatcagaagaagcaccggctaactacgtgccagcagccgcggtaatacgtagggtg
cgagcgttaatcggaattactgggcgtaaagcgtgcgcaggcggttgtgtaagacaggtgtgaaatccccgggcttaacctgggaactgcgctt
gtgactgcacagctagagtacggcagaggggggtggaattccacgtgtagcagtgaaatgcgtagagatgtggaggaacaccgatggcgaag
gcagccccctgggcctgtactgacgctcatgcacgaaagcgtggggagcaaaca

>dd68c7b3d6b665f362d388f2f1be6663

tagggaatcttccgcaatggacgcaagtctgacggagcaacgccgcgtgagtgaagaaggatttcggttcgtaaaactctgttgttagagaaga
acagcgcatagagtaactgttatgcgtgtgacggtatctaaccagaaagccacggctaactacgtgccagcagccgcggtaatacgtaggtggc
aagcgttgtccggatttattgggcgtaaagcgagcgcaggcggtcaattaagtctgatgtgaaagcccccggctcaaccggggagggtcattgg
aaactggttgacttgagtgcagaagaggagagtggaattccatgtgtagcggtgaaatgcgtagatatatggaggaacaccagtggcgaaggc
gactctctggtctgtaactgacgctgaggctcgaaagcgtgggtagcaaaca

>dde5c80a3ae5f9f4fc154fca53d47c65

tagggaatcttccgcaatggacgaaagtctgacggagcaacgccgcgtgtatgaagaaggttttcggatcgtaaagtactgttgttagagaaga
acaaggataagagtaactgcttgtcccttgacggtatctaaccagaaagccacggctaactacgtgccagcagccgcggtaatacggagggtgc
gagcgttaatcggaattactgggcgtaaagcgcgcgtaggcggctaagtcagtcagatgtgaaagccccgggctcaacctgggaattgcacctg
atactgcttggctagagtacagaagagggtggtggaatttcctgtgtagcggtgaaatgcgtagatataggaaggaacatcagtggcgaaggc
ggccacctggactgatactgacgctgaggtgcgaaagcgtggggagcaaaca

>de9c002e5dfaa2513a2f534e7801b528

tagggaatcttccgcaatggacgaaagtctgacggagcaacgccgcgtgtgtgaagaaggtcttcggatcgtaaagcactgttattagagaaga
acacccggtagagtaactgttaccggtctgacggtatctaaccagaaagccacggctaactacgtgccagcagccgcggtaatacgtaggtggc
aagcgttgtccggatttattgggcgtaaagggagcgcaggcggtgatttaagtctgatgtgaaagcccacggctcaaccgtggaaggtcattgg
aaactggatcacttgagtgcagaagaggaaagtggaattccatgtgtagcggtgaaatgcgtagatatatggaggaacaccagtggcgaaggc
gactttctggtctgtaactgacgctgaggctcgaaagcatggggagcaaaca

>df13d60ffb78f06aac8b366bd669191a

tggggaatattggacaatgggcgaaagcctgatccagtgatatcgctttggggatgacggcgacatacgttgtaaacccaatgaagtacgttta
gattatgaccgtacgtattttagaagtcccggctaatctcgtgccagcagccgcggtaatacgagaggggcaagcgttattcaaaatgattgggc
gtaaagcgcacgtaggtggtgatatgtatccataattattcaggctacaagtcttgtcgatatttatgatgtatatgtatttcttgagggtgtgaga
ggagcatggaatttcctaggaagcggtagaatgtgttgatctaggaaggaacaccaatagcgaaagcagtgctctggcacattcctgacattga
ggtgcgaaagcgtggggatcaaaag

>df3d6113f855e22f2a6d44e60f01baa7

tggggaatattgcacaatgggcgaaagcctgatgcagcaacgccgcgtgagcgatgaaggccttcgggtcgtaaagctctgtcctcaaggaag
ataatgacggtacttgaggaggaagccccggctaactacgtgccagcagccgcggtaatacgtagggggctagcgttatccggaattactgggc
gtaaagggtgcgtaggtggtttcttaagtcagaggtgaaaggctacggctcaaccgtagtaagcctttgaaactgggaaacttgagtgcaggag
aggagagtggaattcctagtgtagcggtgaaatgcgtagatattaggaggaacaccagttgcgaaggcggctctctggactgtaactgacactg
aggcacgaaagcgtggggagcaaaca

>df829f8d3288011e9fbef9d3511e2e4c

tagggaatcttccacaatgggcgaaagcctgatggagcaacgccgcgtgtgtgatgaagggtttcggctcgtaaaacactgttgtaagagaaga
atgacattgagagtaactgttcaatgtgtgacggtatcttaccagaaaggaacggctaaatacgtgccagcagccgcggtaatacgtatgttcca
agcgttatccggatttattgggcgtaaagcgagcgcagacggttatttaagtctgaagtgaaagccctcagctcaactgaggaattgctttggaa
actggatgacttgagtgcagtagaggaaagtggaactccatgtgtagcggtgaaatgcgtagatatatggaagaacaccagtggcgaaggcgg
ctttctggactgtaactgacgttgaggctcgaaagtgtgggtagcaaaca

>e00229e46bf3f66c4ffd1b34f3d28b95

tggggaatattgcgcaatgggcgaaagcctgacgcagccacgccgcgtgagcgatgaaggccttagggtcgtaaagctctgtggagggagaag
aaacctgacggtatccctttagcaagcaccggctaactccgtgccagcagccgcggtaatacggagggtgcaaacgttgctcggaatcattggg
cgtaaagcacatgtaggcggctacgcaagttggatgtgaaagcccagggctcaaccctggaagtgcatccaaaactgcgtggcttgagttccgg
aggggcccagagaattcccggtgtagaggtgaaattcgtagatatcgggaggaataccagtggcgaaggcgctgggctggacggatactgacg
ctgagatgtgaaagcgtggggagcaaaca

>e08e7bbe61ff67455f02b7db83918945

tagggaatcttggacaatgggcgaaagcctgatccagcaataccatgtgagtgatgaaggctatttagctgtaaagctctttcatcaaggaagat
aatgacggtacttgaagaagaagccccggctaaccctgtgccagcagccgcggtaagacaggggggctagtgttattcggaatgactgggcg
taaagggtgcgtaggcggtaaataaagttgaaggtgaaatcccaaagcttaactttggaagtgcgttcaaaacttatttgctagagtatgataga
ggatagtggaatttctagtgtagagataaaattcgcaaagactagaaggaacaccagtggcgaaggcgactatctgggtcaatactgacgctg
aggcacgaaagcatggggagcaaacg

>e094c0f86adf02b88862d68fa900feed

tagggaatcttccgcaatggacgaaagtctgacggagcaacgccgcgtgtatgaagaaggttttcggatcgtaaagtactgttgttagagaaga
acaaggataagagtaactgcttgtcccttgacggtatctaaccagaaagccacggctaactacgtgccagcagccgcggtaatacgtaggtggc
aagcgttgtccggatttattgggcgtaaagcgcgcgcaggcggtcttttaagtctgatgtgaaagcccccggcttaaccggggagggtcattgga
aactggaagactggagtgcagaagaggagagtggaattccacgtgtagcggtgaaatgcgtagagatgggaaggaacatcagtggcgaagg
cgactcactggactgatactgacactgaggtgcgaaagcgtgggtagcaaaca

>e1058c7ff3fb0b864735b0d3d2cdee72

tgaggaattttccgcaatgggcgaaagcctgacggagcaataccgcgtggaggatgacggcctgtgggtcttaaactcctttttcttagagaaga agttctgacggtatctaaggaataagcatcggctaactccgtgccagcagccgcggtaatacgggggatgcaagcgttatccggaatgattggg cgtaaagcgtctgtaggtggctttccaagtcaactgtcaaaaatcagggcttaaccttgagccggcagttgaaactatgaagcttgagttaggta ggggcagagggaattcctagtgtagcggtgaaatgcgtagatattaggaagaacaccaatcgcgaaagcactctgctgggccatgactgacac tgagagacgaaagctaggggagcgaatgggattagataccccggtag

>e208c28191054d655aea79022ef47a06

tagggaatcttccgcaatggacgaaagtctgacggagcaacgccgcgtgtatgaagaaggttttcggatcgtaaagtactgttgttagagaaga acaaggataagagtaactgcttgtcccttgacggtatctaaccagaaagccacggctaactacgtgccagcagccgcggtaatacggagggtgc gagcgttaatcggaattactgggcgtaaagcgcgcgtaggcggctttgtaagtcggatgtgaaagccctgggctcaacctgggaattgcactcg atactgcagagctagagtatggtagagggaagtggaatttccggtgtagcggtgaaatgcgtagatatcggaaggaacaccagtggcgaaggc gacttcctgggccaatactgacgctgaggtgcgaaagcgtggggagcaaaca

>e22b45cc5c2b9e6cbdcd849f8b00036c

tggggaatattggacaatgggcgaaagcctgatccagcaatatcgcgtgagtgaagaaggctattttggtcgtaaagctctttcgtcgaggaag ataatgacggtactcgaagaagaagtcccggctaactccgtgccagcagccgcggtaagacggaggggacgagtgttattcggaatgactggg cgtaaagggcacgtaggcggtgaattaagttgaaagcgaaatcccaaagcttaactttggaactgttttcaaaactgatttactggagttcgata ggggatagtggaatttctagtgtagaaatagaattcataaatattagaaggaacaccagtggcgaaggcgactatctggatcgatactgacgct gaggtgcgaaagcatggggagcaaaca

>e293a4a9c3d5e6cfc55621b8c058045b

tgaggaatattggacaatgggcgcaagcctgatccagccatgccgcgtgcaggatgaagcatctatggtgtgtaaactgcttttgtacgagaag aaacactgcaacgtgttgcagcttgacggtatcgtaagaataaggatcggctaactccgtgccagcagccgcggtaatacggaggatccaagc gttatccggaatcattgggtttaaagggtccgtaggcgggccagtaagtcagtggtgaaatctcccggctcaaccgggaaatggccattgatact gctggtcttgaattattaggaagtaactagaatatgtagtgtagcggtgaaatgcttagagattacatggaataccaattgcgaaggcaggttac tactaatggattgacgctgatggacgaaagcgtgggtagcgaaca

>e2a5a5b2e0703a3656f8a0e9795a031a

tttcgaatcattcacaatgggcgaaagcctgatggtgcgacgccgcgtgagggatgaaggccttcgggttgtaaacctctgtcaccggggaaga aacgcttcaagttaatagcttgaagcctgacttaacccggagaggaagcagtggctaactctgtgccagcagccgcggtaatacagagactgca agcgttattcggattcactgggcgtaaagggtgcgcaggcggccgagtgtgtgaggcgtgaaagcccggagcttaactccggaattgcacctca aactacacggctagagcattggagagggtagcagaattcacggtgtagcagtgaaatgcgtagatatcgtgaggaataccagaggcgaaggc ggctacctggacaattgctgacgctcaggcacgaaagcgtggggagcaaaag

>e301db4993d117481300e9f0d72d7bb7

caaggaattttcgtcaatgggcgcaagcctgaacgagcaacgccgcgtggaggatgacggccttcgggttgtaaactccttttccgggggatgat agtgaccgtaccctgggaataaggctcggctaactctgtgccagcagccgcggtaagacagaggagccaagcgttgtccggattgactgggcgt aaagcgcacgcaggcggttgggcacgtggggtgtgaaagcggcccgcttaacgggccgaggccatcccatacggcccgactggagcctggca gaggttcgtggaattgccggtgtagtggtgaaatgcgtagagatcggcaggaacaccaagggggaaaccagcgaactgggccagagctgacg ctcaggtgcgaaagcgtggggagcgaacc

>e349404d219f149d829618cd9650bf32

tggggaattttccgcaatgggcgaaagcctgacggagcaatgccgcgtggaggtagaaggcccacgggtcatgaacttcttttctcagagaaga agcaatgacggtatctgaggaataagcatcggctaactctgtgccagcagccgcggtaagacagaggatgcaagcgttatccggaatgattgg gcgtaaagcgtctgtaggtggcttttcaagtccgccgtcaaatcccagggctcaaccctggacaggcggtggaaactaccaagctggagtacgg

taggggcagagggaatttccggtggagcggtgaaatgcgtagagatcgggaagaacaccaacggcgaaagcactctgctgggccgacactga
cactgagagacgaaagctaggggagcaaatg

>e38f9e2b18a62d89a2dd8511cc8a43ad

tggggaatattgggcaatggacgaaagtctgacccagcaacgccgcgtgggggatgaaggccttagggttgtaaactccttttattagggaaga
tagtgacggtacctaatgaataagcatcggctaacttcgtgccagcagccgcggtaatacgaaggatgctagcgttactcggaattactgggcgt
aaagaatgcgtaggcgggcatttaagttgatagtgcaatccccaagcttaacttaggaattgctatcaaaactaaatgccttgaatatgaaaggg
agcaagggaattcctggtgtaggggtaaaatccgtagatatcaggaggaagaccggaagcgaaagcgcttgcttggttcattattgacgctgag
gcatgaaagcgtggggatcgaacaggattagatacccggtagtc

>e49ced5d648cd008cb8fdbb84b219c2d

cagggaatattgcacaatgggcgaaagcctgatgcagcaacgccgcgtgggggatgaaggccttcgggttgtaaacccctttcaggagggaag
aaattgacggtacctccaaaagaagccccggccaactacgtgccagcagccgcggtaatacgtagggggcgagcgttgtccggaatcattggg
cgtaaagggctcgtaggcggctcagttagtcggctgtgaaatcccagggctcaaccctggaactgcagtcgatactgctgtggctagagtttggt
agaggagaatggaattcccggtgtagcggtggaatgcgcagatatcgggaggaacaccagtagcgaaggcggttctctgggccaatactgacg
ctgaggagcgaaagcgtggggagcaaaca

>e6632ac9c710d04aa57f542d933fdd7c

tggggaatcttggacaatgggcgcaagcctgatccagccatgccgcgtgagtgatgaaggccttagggtcgtaaagctctttcgccagggatga
taatgacagtacctggtaaagaaaccccggctaactccgtgccagcagccgcggtaatacggagggggttagcgttgttcggaattactgggcg
taaagcgcacgtaggcggatcagaaagttgggggtgaaatcccggggctcaaccccggaactgcctccaaaactcctggtcttgagttcgagag
aggtgagtggaattccgagtgtagaggtgaaattcgtagatattcggaggaacaccagtggcgaaggcggctcactggctcgatactgacgctg
aggtgcgaaagtgtggggagcaaaca

>e68f9542b5b4ef063c352c3973e8521c

tgaggaatattcggcaatgggggcaaccctgaccgagcaacgccgcgtggaggatgacggttttcggattgtaaactcctttttctcagggaaga
gcgaggacggtacctggggaataagtcacggctaactacgtgccagcagccgcggtaatacgtaggtggcaagcgttatccggaattactggg
cgtaaagcgcacgcaggtggctgcttaagtctgacgtgaaagctcctggcttaactgggagaggtcgttggagactgggcggcttgaggcgagg
agaggggtgtagaattcccggtgtagtggtggaatgcgtagagatcgggaggaataccagtggcgaaagcggcaccctggccttggcctgacg
ctcaggtgcgaaagcgtggggagcgaacg

>e6c461ee005018c33202161446917049

tggggaatcttggacaatgggcgaaagcccgatccagcaatatcgcgtgagtgaagaagggcaatgccgcttgtaaagctctttcgtcgagtgc
gcgatcatgacaggactcgaggaagaagccccggctaactccgtgccagcagccgcggtaagacgggggggcaagtgttcttcggaatgact
gggcgtaaagggcacgtaggcggtgaatcgggttgaaagtgaaagtcgccaaaaagtggcggaatgctctcgaaaccaattcacttgagtgag
acagaggagagtggaatttcgtgtgtaggggtgaaatccgtagatctacgaaggaacgccaaaagcgaaggcagctctctgggtccctaccga
cgctggggtgcgaaagcatggggagcgaacaggattagataccccggtagt

>e70ea086af7238e1d7addef62a9e2fde

tagggaatcttgcgcaatgggcgaaagcctgacgcagcaatgccgcgtgcgggacgaaggccctagggtcgtaaaccgctttcagtagggacg
aaaatgacggtacctgcagaagaagctccggccaactacgtgccagcagccgcggtgatacgtagggagcaagcgttgtccggaattactggg
cgtaaagggctcgtaggtggttgagtaagtcagatgtgaaatctcagggcccaaccctgagcgtgcatttgatactgctctgactagagtccggt
aggggagtgcggaattcctggtgtagcggtgaaatgcgcagatatcaggaggaacaccgacagcgaaggcagcactctgggccggtactgac
actgaggagcgaaagcatgggtagcaaaca

>e78b3edf997b6046c03ad4c3e640cdd4

tggggaatattgcgcaatgggcgaaagcctgacgcagcgacgccgcgtgagggacgaaggtcttcggattgtaaacctctttcagcagggatg
aagcgaaagtgacagtacctgcagaagaagcaccggctaactacgtgccagcagccgcggtaatacgtagggtgcaagcgttgtccggattta
ttgggcgtaaagagctcgtaggcggtctgtcgcgtcggatgtgaaaacccaatggcttaaccttgggcctgcatacgatacgggcagactagag
tgcggtaggggagactggaattcctggtgtagcggtggaatgcgcagatatcaggaggaacaccgatggcgaaggcaggtctctgggccgtaa
ctgacgctgaggagcgaaagcgtggggagcaaaca

>e7de1381c9f32010c03fb6b777b416c0

tggggaattttccgcaatgggcgaaagcctgacggagcaatgccgcgtggaggtggaaggcctacgggtcgtcaacttcttttctcggagaaga
aacaatgacggtatctgaggaataagcatcggctaactctgtgccagcagccgcggtaagacagaggatgcaagcgttatccggaatgattgg
gcgtaaagcgtctgtaggtggcttttcaagtccgccgtcaaatcccagggctcaaccctggacaggcggtggaaactaccaagctggagtacgg
taggggcagagggaatttccggtggagcggtgaaatgcattgagatcggaaagaacactaacggcgaaagcactctgctgggccgacactga
cactgagagacgaaagctaggggagcaaatg

>e871f2c7a13331aad8aba52c17b4b3ea

tcgagaatcttcggcaatgggcgcaagcctgaccgagcgacgccgcgtggaggacgaaggccttcgggttgtaaactcctgtcgaggggaaga
aaggccgagcaatcggcttgatcgacccctggaggaagcacgggctaagttcgtgccagcagccgcggtaagacgaaccgtgcgaacgttatt
cggaatcactgggcttaaagcgcgtgtaggcggattggtgcgtcggcgcctgaaatcccccggctcaaccggggaacgtggcccgaaacgacc
agtctggaggggcgtaggggggaactggaacttccggtggagcggtgatatgcgttgagatcggaaggaacgcccgtggcgaaagcgagttcct
ggacgcctgctgacgctgagacgcgaaagccagggtagcgaacg

>e89f13d4476a6aac75bc5c27fcb1d7fe

tggggaattttccgcaatgggcgaaagcctgacggagcaatgccgcgtggaggtggaaggcctatgggtcgtcaacttcttttctcggagaaga
aacaatgacggtatctgaggaataagcatcggctaactctgtgccagcagcccgcggtaagacagaggatgcaagcgttatccggaatgattg
ggcgtaaagcgtctgtaggtggcttttcaagtccgccgtcaaatcccagggctcaaccgtggacaggcggtggaaactaccaagctggagtacg
gtaggggcagagggaatttctggtggagcggtgaaatgcattgagatcggaaagaacaccaacggcgaaagcactctgctgggccgacactg
acactgagagacgaaagctaggggagcaaatg

>e8b7de4c07f308c714246ef5c937f9c9

tggggaatcttggacaatgggcgaaagcccgatccagcaatatcgcgtgagtgaagaagggcaatgccgcttgtaaagctctttcgtcgagtgc
gcgatcatgacaggactcgaggaagaagccccggctaactccgtgccagcagccgcggtaagacgggggggggcaagtgttcttcggaatgact
gggcgtaaagggcacgtaggcggtgaatcgggttgaaagtgaaagtcgccaaaaagtggcggaatgctctcgaaaccaattcacttgagtgag
acagaggagagtggaatttcgtgtgtaggggtgaaatccgtagatctacgaaggaacgccaaaagcgaaggcagctctctgggtccctaccga
cgctggggtgcgaaagcatggggagcgaaca

>e8e068470c9fa4a80add9d39995d4252

taaggaatattggacaatgggcgaaagcctgatccagccatcccgcgtggaggatgactgccctacgggttgtaaactccttttatatgagaaga
aacgcctggatttatccgggcctgacggtatcttatgaataagcaccggctaactccgtgccagcagccgcggtaatacggagggtgcaagcgtt
atccggaatcactgggtttaaagggtgcgtaggcggttagttaagtcagaggtgaaagcccggcgctcaacgtcggaattgcctttgatactggc
taacttgaatcaggttgaggtgggcggaatgtggcatgtagcggtgaaatgcatagatatgccatagaacaccaattgcgaaggcagctcactg
gccctgcattgacgctgaggcacgaaagcgtggggagcgaaca

>e94a13a5e4c68e630850a0d843b36e7e

tggggaattttggacaatgggcgcaagcctgatccagccatgccgcgtgtgggaagaaggccttcgggttgtaaaccacttttgtcagggaaga
aacggtttgacttaatacgttgaactaatgacggtacctgaagaataagcaccggctaactacgtgccagcagccgcggtaatacgtagggtgc
aagcgttaatcggaattactgggcgtaaagcgtgcgcaggcggctttgcaagacagatgtgaaatccccgggctcaacctgggaactgcatttg
tgactgcatggctagagtacggtagagggggatggaattccgcgtgtagcagtgaaatgcgtagatatgcggaggaacaccgatggcgaaggc
aatcccctggacctgtactgacgctcatgcacgaaagcgtggggagcaaaca

>eabef75613caeaaf66d8f72ece33ff98

tggggaatattgcacaatgggcgcaagcctgatgcagccatgccgcgtgtgtgaagaaggccttcgggttgtaaagcactttcagtcgtgagga
aggtggtagtgttaatagcactatcatttgacgttagcgacagaagaagcaccggctaactccgtgccagcagccgcggtaatacggagggtgc
gagcgttaatcggaattactgggcgtaaagcgtacgcaggcggtttgttaagcgagatgtgaaagccccgggctcaacctgggaactgcatttc
gaactggcaaactagagtgtgatagagggtggtagaatttcaggtgtagcggtgaaatgcgtagagatctgaaggaataccgatggcgaaggc
agccacctgggtcaacactgacgctcatgtacgaaagcgtggggagcaaaca

>eaeaed05611e993f4383055a77762c33

tggggaattttccgcaatgggcgaaagcctgacggagcaatgccgcgtggaggtggaaggcctacgggtcgtcaacttcttttctcggagaaga
aacaatgacggtatctgaggaataagcatcggctaactctgtgccagcagccgcggtaagacagaggatgcaagcgttatccggaatgattgg
gcgtaaagcgcctgtaggtggcttttcaagtccgccgtcaaatcccagggctcaaccctggacaggcggtggaaactaccaagctggagtacgg
taggggcagagggaatttccggtggagcggtgaaatgcattgagatcggaaagaacaccaacggcgaaagcactctgctgggccgacactga
cactgagagacgaaagctaggggagcaaatg

>ebbee8345c0db5ed47362d0bd541402e

tggggaattttccgcaatgggcgaaagcctgacggagcaataccgcgtgagggatgacggcctatgggttgtaaacctctttttcagggagga
atcaatgacgtgtacctgaagaataagcatcggctaactccgtgccagcagccgcggtaagacggaggatgcaagtgttatccggaatcactgg
gcgtaaagcgtctgtaggtggtccaataagtcaactgttaaatcttgaggctcaacctcaaaatcgcagtcgaaactattagactagagtatagt
aggggtaaagggaatttccagtggagcggtgaaatgcgtagagattggaaagaacaccgatggcgaaggcactttactgggctattactaaca
ctcagagacgaaagctagggtagcaaatg

>ec098ad12ef2923b449a01762462578b

tagggaatcttcggcaatggacgaaagtctgaccgagcaacgccgcgtgagtgaagaaggttttcggatcgtaaaactctgttggtagagaaga
acgttggtgagagtggaaagctcatcaagtgacggtaactacccagaaagggacggctaactacgtgccagcagccgcggtaatacgtaggtc
ccgagcgttgtccggatttattgggcgtaaagcgagcgcaggtggtttattaagtctggtgtaaaaggcagtggctcaaccattgtatgcattgga
aactggtagacttgagtgcaggagaggagagtggaattccatgtgtagcggtgaaatgcgtagatatatggaggaacaccggtggcgaaagcg
gctctctggcctgtaactgacactgaggctcgaaagcgtggggagcaaaca

>ec7e081e7a9c7a218c1ba9d999cadc46

tggggaatcttggacaatgggcgaaagcccgatccagcaatatcgcgtgagtgaagaagggcaatgccgcttgtaaagctctttcgttgagtgc
gcgatcatgacaagactcgaggaagaagccccggctaactccgtgccagtagccgcggtaagacggggggggggggcaagtgttcttcgaaat
gactgggcgtaaagggcacgtaggcggtgaatcgggttgaaagtgaaagtcgccaaaaagtggcggaattctctcgaaaccaattcacttgag
tgagacagaggagagtggaatttcgtgtgtaggggtgaaatctgtagatctacgaaggaacaccaaaagcgaaggcagctctctgggtccctа
ccgacgctggggtgcgaaagcatggggagcgaaca

>eca6f7993f6fc5a4be346d425d8eb8e6

tggggaatattgcgcaatgggcgaaagcctgacgcagcgacgccgcgtgagggacgacggccttcgggttgtaaacctctttcagcagggacg
aaaatgacggtacctgcagaagaagcaccggctaactacgtgccagcagccgcggtaatacgtagggtgcaagcgttgtccggatttattgggc
gtaaagagctcgtaggcggtctgtcgcgtcggatgtgaaaacttggggctcaaccctgagcttgcattcgatacgggcagactagaggattgca
ggggagactggaattcctggtgtagcggtggaatgcgcagatatcaggaggaacaccggtggcgaaggcgggtctctgggcaattactgacgc
tgaggagcgaaagcgtggggagcaaaca

>ed26e6a1f558f75e04f278bfea71336c

tggggaatattggacaatgggcgcaagcctgatccagcaatgccgcgtgagtgatgaaggccttcgggttgtaaagctctttcgcacgcgacga
tgatgacggtagcgtgagaagaagccccggctaacttcgtgccagcagccgcggtaatacgaagggggctagcgttgttcggaattactgggcg
taaagggcgcgtaggcggtgtgtcaagtcaggcgtgaaagccccgggctcaacctgggaaccgcgcttgagactggcacgctcgagttcggga

gaggatggtggaattcccagtgtagaggtgaaattcgtagatattgggaagaacaccgatggcgaaggcagccatctggaccgacactgacgc
tgaggcgcgaaagcgtggggagcaaaca

>edca45bc748f10fbd6a8314634401453

tggggaatattgcgcaatgggcgaaagcctgacgcagcgacgccgcgtgagggacgaaggtcttcggattgtaaacctctttcagcagggacg
aagcgaaagtgactgtacctgcagaagaagcaccggctaactacgtgccagcagccgcggtaatacgtagggtgcaagcgttgtccggatttat
tgggcgtaaagagctcgtaggcggtctgtcgcgtcggatgtgaaaacccaatggcttaaccttgggcctgcattcgatacgggcagactagagt
gtggtaggggaaactggaattcctggtgtagcggtggaatgcgcagatatcaggaggaacaccgatggcgaaggcaggtttctgggccataac
tgacgctgaggagcgaaagcgtggggagcaaaca

>ede5e8c8c62c4609d98e2c130d1c4e90

tggggaattttccgcaatgggcgaaagcctgacggagcaatgccgcgtggaggtagaaggcccacgggtcatgaacttcctttcccggagaag
aagcaatgacggtatctggggaataagcatcggctaactctgtgccagcagccgcggtaatacagaggatgcaagcgttatccggaatgattgg
gcgtaaagcgtctgtaggtggctttttaagtccgccgtcaaatcccagggctcaactctggacaggcggtggaaactaccaagctggagtacggt
aggggcagagggaatttccggtggagcggtgaaatgcgtagagatcggaaagaacaccaacggcgaaagcactctgctgggccgacactgac
actgagagacgaaagctaggggagcgaatg

>eefe9cbaae27d5ee618e0fd3e48dbb14

tggggaatattggacaatgggggaaaccctgatccagcaatgccgcgtgtgtgaagaaggcctgcgggttgtaaagcactttcagtagagaag
aaaagcctatggttaataaccgtgggtcttgacgtaacctacagaagaagctccggctaactccgtgccagcagccgcggtaatacggaggga
gcgagcgttaatcggaattactgggcgtaaagcgcgcgtaggcggctttgtaagtcggatgtgaaagccctgggctcaacctgggaattgcact
cgatactgcagagctagagtatggtagagggaagtggaatttccggtgtagcggtgaaatgcgtagatatcggaaggaacaccagtggcgaag
gcgacttcctgggccaatactgacgctgaggtgcgaaagcgtggggagcaaaca

>f13e19ab6677af0872877e771d828137

tggggaattttccgcaatgggcgaaagcctgacggagcaatgccgcgtggaggtagaggcctctgggtcatgaacttcttttcccggagaagaa
aaaatgacggtatccggggaataagcatcggctaactctgtgccagcagccgcggtaagacagaggatgcaagcgttatccggaatgattggg
cgtaaagcgtctgtaggtggctttttaagttcgctgtcaaataccagggctcaaccctggacaggtggtgaaaactactaagctagagtacggta
ggggcagagggaatttccggtggagcgatgaaatgcgtagagatcggaaggaacaccaacggcgaaagcactctgctgggccgacactgaca
ctgagagacgaaagctaggggagcgaatg

>f14141d03c23f3658014090c72c23866

tagggaatcttccacaatggacgcaagtctgatggagcaacgccgcgtgagtgaagaaggttttcggatcgtaaagctctgttgttggtgaaga
aggatagaggtagtaactggcctttatttgacggtaatcaaccagaaagtcacggctaactacgtgccagcagccgcggtaatacgtaggtggc
aagcgttgtccggatttattgggcgtaaagcgagcgcaggcggaagaataagtctgatgtgaaagccctcggcttaaccgaggaactgcatcgg
aaactgtttttcttgagtgcagaagaggagagtggaactccatgtgtagcggtggaatgcgtagatatatggaagaacaccagtggcgaaggcg
gctctctggtctgcaactgacgctgaggctcgaaagcatgggtagcgaaca

>f1c0b9b1939edb9092f76db8621ea38f

tggggaatattggacaatgggcgaaagcctgatccagccatgccgcgtgtgtgaagaaggtcttcggattgtaaagcactttaagttgggagga
agggttgtagcttaatacgctgcaaccttgacgttaccaacagaataagcaccggctaactctgtgccagcagccgcggtaatacagagggtgc
aagcgttaatcggaattactgggcgtaaagcgcgcgtaggcggcttgataagatgggtgtgaaatccccgggcttaacctgggaactgcatcca
taactgtctggctagagtacagtagagggtggtggaatttcctgtgtagcggtgaaatgcgtagatataggaaggaacaccagtggcgaaggcg
accacctggactgatactgacgctgaggtgcgaaagcgtggggagcaaaca

>f21040166a8a29c0a2e34173c51e4bb5

tggggaattttccgcaatgggcgaaagcctgacggagcaatgccgcgtggaggtagaaggcccacgggtcatgaacttcttttcccggagaaga
agcaatgacggtatctggggaataagcatcggctaactctgtgccagcagccgcggtaatacagaggatgcaagcgttatccggaatgattggg
cgtaaagcgtctgtaggtggctttttaagtccgccgtcaaatcccagggctcaactctggacaggcggtggaaactaccaagctggagtacggta
ggggcagagggaatttccggtggagcggtgaaatgcgtagagatcggaaagaacaccaacggcgaaagcactctgctgggccgacactgaca
ct

>f279ba5e72083ec5c385be0aa40168c2

tggggaatcttggacaatgggcgaaagcccgatccagcaatatcgcgtgaatgaagaagggcaatgccgcttgtaaagctctttcgtcgagtgc
gcgatcatgacaggactcgaggaagaagccccggctaactccgtgccagcagccgcggtaagacgggggggcaagtgttcttcggaatgact
gggcgtaaagggcacgtaggcggtgaatcgggttgaaagtgaaagtcgccaaaaagtggcggaatgctctcgaaaccaattcacttgagtgag
acagaggagagtggaatttcgtgtgtaggggtgaaatccgtagatctacgaaggaacgccaaaagcgaaggcagctctctgggtccctaccga
cgctggggtgcgaaagcatggggagcgaaca

>f2c12d84846d66c69e5d3b6f247b687f

tagggaatcttccgcaatggacgaaagtctgacggagcaacgccgcgtgtatgaagaaggccttttggttgtaaagcactttaagcgaggagga
ggctaccgagattaatactcttggatagtggacgttactcgcagaataagcaccggctaactctgtgccagcagccgcggtaatacagagggtg
caagcgttaatcggatttactgggcgtaaagcgcgcgtaggtggccaattaagtcaaatgtgaaatccccgagcttaacttgggaattgcattcg
atactggttggctagagtatgggagaggatggtagaattccaggtgtagcggtgaaatgcgtagagatctggaggaataccgatggcgaaggc
agccatctggcctaatactgacactgaggtgcgaaagcatggggagcaaaca

>f2f9aa93e31efe482f836c89e44f5635

tggggaatcttggacaatgggcgaaagcccgatccagcaatatcgcgtgagtgaagaagggcaatgccgcttgtaaagctctttcgtcgagtgc
gcgatcatgacaggactcgaggaagaagccccggctaactccgtgccagcagccgcggtaagacgggggggcaagtgttcttcggaatgact
gggcgtaaagggcacgtaggcggtgaatcgggttgaaagtgaaagtcgccaaaaagtggcggaatgctctcgaaaccaattcacttgagtgag
acagaggagagtggaatttcgtgtgtaggggtgaaatccgtagatctacgaaggaacgccaaaagcgaaggcagctctctgggtccctaccga
cgctggggtgcgaaagcatggggagcgaacaggattagatacccgtgtag

>f33d52caf28dd26a8da9b90d8d6374ab

tggggaatcttggacaatgggcgaaagcccgatccagcaatatcgcgtgagtgaagaagggcaatgccgcttgtaaagctctttcgtcgagtgc
gcgatcatgacaggactcgaggaagaagccccggctaactccgtgccagcagccgcggtaagacgggggggcaagtgttcttcggaatgact
gggcgtaaagggcacgtaggcggtgaatcgggttgaaagtgaaagtcgccaaaaagtggcggaatgctctcgaaaccaattcacttgagtgag
acagaggagagtggaatttcgtgtgtaggggtgaaatccgtagatctacgaaggaacgccaaaagcgaaggcagctctctgggtccctaccga
cgctggggtgcgaaagcatggggagcgaacaggattagataccccgggtagtc

>f487f0b5f9e5ec05065c687066960220

tagggaatcttccacaatggacgcaagtctgatggagcaacgccgcgtgagtgaagaaggttttcggatcgtaaagctctgttgttggtgaaga
aggatagaggtagtaactggcctttatttgacggtaatcaaccagaaagtcacggctaactacgtgccagcagccgcggtaatacgtaggtggc
aagcgttgtccggatttattgggcgtaaagcgagcgcaggcggaagaataagtctgatgtgaaagccctcggcttaaccgaggaactgcatcgg
aaactgtttttcttgagtgcagaagaggagagtggaactccatgtgtagcggtggaatgcgtagatatatggaagaacaccagtggcgaaggcg
gctctctggtctgcaactgacgctgaggctcgaaagcatgggtagtgaaca

>f4a18715487abbc3431cc939c4e66d8b

tggggaattttccgcaatgggcgaaagcctgacggagcaatgccgcgtggaggtagaaggcccacgggtcatgaacttcttttcccggagaaga
agcaatggcggtatctggggaataagcatcggctaactctgtgccagcagccgcggtaatacagaggatgcaagcgttatccggaatgattggg
cgtaaagcgtctgtaggtggctttttaagtccgccgtcaaatcccagggctcaactctggacaggcggtggaaactaccaagctggagtacggta
ggggcagagggaatttccggtggagcggtgaaatgcgtagagatcggaaagaacaccaacggcgaaagcactctgctgggccgacactgaca
ctgagagacgaaagctaggggagcgaatg

>f4ad46eb826b9b885a5b2a4237bb2435

tgaggaattttctgcaatgggcgaaagcctgacagagcaataccgcgtgggggatgaagacttactgagttgtaaacctcggtaccctaaggaa
gaagatatgacggtacttagggtggaaagcatcggctaactccgtgccagcagccgcggtaagacgggggatgcaagtgttatccggatttact
gggcgtaaagcgtctgcaggtggtttcttaagtctactgttaaatcttgaggctcaacctcaaatctgcagtagaaactaggagacttgagtatag
taggggtagagggaatttccagtggagcggtgaaatgcgtagatattggaaagaacaccgatggcgaaggcactctactgggctattactgac
actcagagacgaaagctaggggagcaaatg

>f579c8348a414fa66afc693b36cd87d1

tggggaatattgcacaatgggcgaaagcctgatgcagccatgccgcgtgtatgaagaaggccttcgggttgtaaagtactttcagttgtgagga
agggtgtgtagttaatagctgcgcatcttgacgttagcaacagaagaagcaccggctaactccgtgccagcagccgcggtaatacgggggatgc
aagcgttatccggaatgattgggcgtaaagcgtctgtaggtggctttttaagtccgccgtcaaatcccagggctcaactctggacaggcggtgga
aactaccaagctggagtacggtaggggcagagggaatttccggtggagcggtgaaatgcgtagagatcggaaagaacaccaacggcgaaag
cactctgctgggccgacactgacactgagagacgaaagctaggggagcgaatg

>f5dfca2eea90cca7c7e732418a9fa5fa

taaggaatattggacaatggacgcaagtctgatccagccatgccgcgtggtggatgacggccctctgggttgtaaacaccttttatgtggggata
atgtggttctatatgggccattgcaggtaccacatgaataagcaccggctaactacgtgccagcagccgcggtaatacgtagggtgcaagcgttg
tccggatttattgggtttaaagggtgcgcaggcggcttattaagttagtggtgaaatcacccggctcaaccgggtacccgccattgatactggtaa
gcttgagtatgttcgaggagattggaacgtgtagtgtagcggtgaaatgcatagatattacgcagaacaccaattgcgaaggcagatctctggg
acataactgacgctgaggcacgaaagcgtggggagcgaaca

>f67d7ff7cf8b9b950e802bc307642b82

tggggaattttccgcaatgggcgaaagcctgacggagcaatgccgcgtggaggtagaaggcccacgggtcgtgaacttcttttcccggagaaga
agcaatgacggtatctgaggaataagcatcggctaactctgtgccagcagccgcggtaagacagaggatgcaagcgttatctggaatgattgg
gcgtaaggcgtctgtaggtggcttttcaagtccgccgtccaatcccagggctcaaccctggacaggcggtggaaactaccaagctggagtacgg
taggggcagagggaatttccggtggagcggtgaaatgcgtagagatcggaaagaacaccaacggcgaaagcaccctgctgggccgacactga
cactgagagacgaaagctaggggagcgaatg

>f7935da54a9f99b58a368c52bc9871b7

tggggaatattgcacaatgggcggaagcctgatgcagcaacgccgcgtgcgggatgacggccttcgggttgtaaaccgctttcatccatgacga
agcgagagtgacggtagtgggagaagaagcaccggctaactacgtgccagcagccgcggtgatacgtagggtgcgagcgttgtccggatttat
tgggcgtaaagagcttgtaggcggtttgtcgcgtcgggagtgaaaactcagggcttaaccctgagcctgctttcgatacgggcagacttgaggaa
ggtaggggagaatggaatttctggtgaagcggtggaatgcgtagatatcagaaggaacaccagtggcgaaggcggttctctggacctttcctga
cgctgagaagcgaaagcgtggggagcaaaca

>f798c7b55301016fd559eaa70cf87900

tcgagaatcttcggcaatgggcgcaagcctgaccgagcgacgccgcgtgtgggatgaaggcccttgggttgtaaaccactgtcagaggggatg
aaatgcgggtgggttctcccatccgtttgacatagcctcagaggaagcacgggctaagttcgtgccagcagccgcggtaatacgaactgtgcaa
acgttattcggaatcactgggcttaaagggtgcgtaggcggccagataagtagggtgtgaaagcccccggctcaaccggggaatggcgctctaa
actgtctggctggagtgagataggggtgtgcggaacttccggtggagcggtgaaatgtgttgatatcggaaggaacgccggtggcgaaagcgg
cacactggatctcaactgacgctgaggcacgaaagccaggggagcaaacg

>f849e5d3fd5ae6d42d6e7fcf1bc243ee

tgggggatattgcacaatgggcgcaagcctgatgcagcgacgccgcgtgagggatgacggctttcgggttgtaaacctctttcgctaccgaaca
aggctccatattggtggggttgagggtaggtggataagaagcgccggctaactacgtgccagcagccgcggtaatacgtagggcgcgagcgtt
gtccggaattattgggcgtaaagagctcgtaggcggcttgtcgcgtctgctgtgaaaacgcggggcttaactccgcgcgtgcagtgggtacgggc

aggctagagtgtggtaggggtgactggaattcctggtgtagcggtggaatgcgcagatatcaggaggaacaccgatggcgaaggcaggtcact
gggccattactgacgctgaggagcgaaagcatggggagcgaaca

>f8785a63a5bcea2f3adb57194d11bcb9

tagggaatattgggcaatggaggaaactctgacccagccatgccgcgtgaaggatgaaggtcctacggattgtaaacttcttttattagggaag
aaacgtctcgattcatcgggatttgacggtacctaaggaataagcaccggctaactccgtgccagcagccgcggtaatacggagggtgcaagcg
ttatccggattcactgggtttaaagggtgcgtaggtggattgataagtcagtggtgaaagccccgagctcaacttgggaattgccattgatactgt
tagtcttgagttcggttgaggtaggcggaataagtcatgtagcggtgaaatgcttagatatgacttagaacaccgattgcgaaggcagcttacca
agccgacactgacactgaggcacgaaagcgtggggatcaaaca

>f8b1222d060b5192a5b0f025b2aa44bb

tccggaatcttggacaattggcgaaagccagatccagccatattacgtgaaggatgaagactttttggttgtaaacttctttactttttaaagtta
aagagtataaaaagccccggctaaatctgtgccagcagccgcggtaatacagatggggcaagtgttattcggaatgattgggcgtaaagggta
cgtaggtggttttattagtcagttgtcacaacctttttagaaaagaatcgcagctttaaaagactacaaaacttgagtttgatagaggaaagcgg
aattttgagtggagagatagaattcatagatattcaaaggaacaccgaaaacgaaggtagctttctgggtcaacactgacgctcatgtacgaa

>f91b00381116d19f841d5805847ee161

tggggaattttccgcaatgggcgaaagcctgacggagcaatgccgcgtgaaggcagaaggcccacgggtcatgaacttcttttctcggagaaga
aaaaatgacggtatctgaggaataagcatcggctaactctgtgccagcagccgcggtaagacagaggatgcaagcgttatccggaatgattgg
gcgtaaagcgtctgtaggtggcttttcaagtccgccgtcaaatcccagggctcaaccctggacaggcggtggaaactaccaagctggagtacgg
taggggcagagggaatttccggtggagcggtgaaatgcgttgagatcggaaagaacaccaacggcgaaagcactctgctgggccgacactga
cactgagagacgaaagctaggggagcaaatg

>f9c27874a3bd6b3338bb3f4741b1d337

tggggaattttccgcaatgggcgaaagcctgacggagcaatgccgcgtggaggtagaggcccctgggtcatgaacttcttttcccggagaagaa
aaaatgacggtatccggggaataagcatcggctaactctgtgccagcagccgcggtaagacagaggatgcaagcgttatccggaatgattggg
cgtaaagcgtctgtaggtggctttttaagttcgttgtcaaataccagggctcaaccctggacaggtggtgaaaactactaagctagagtacggta
ggggcagagggaatttccggtggagcgatgaaatgcgtagagatcggaaggaacaccaacggcgaaagcactctgctgggccgacactgaca
ctgagagacgaaagctaggggagcgaatg

>fa232cdccf10ffa5f666dfa77563e84e

tggggaattttccgcaatgggcgaaagcctgacggagcaatgccgcgtggaggtggaaggcctacgagtcgtcaacttcttttctcggagaaga
aacaatgacggtatctgaggaataagcatcggctaactctgtgccagcagccgcggtaagacagaggatgcaagcgttatccggaatgattgg
gcgtaaagcgtctgtaggtggcttttcaagtccgccgtcaaatcccagggctcaaccctggacaggcggtggaaactaccaagctggagtacgg
taggggcagagggaatttccggtggagcggtgaaatgcattgagatcggaaagaacaccaacggcgaaagcactctgctgggccgacactga
cactgagagacgaaagctaggggagcaaatg

>fa23a2f9dee1fc701797ae68535a98d5

tggggaatattggacaatgggcgcaagcctgatccagccatgccgcgtgagtgatgaaggccttagggttgtaaagctctttcgtccgggacgat
aatgacggtaccggaagaagaagccccggctaacttcgtgccagcagccgcggtaatacgaagggggctagcgttgttcggaatcactgggcg
taaagggcgcgtaggcggccattcaagtcgggggtgaaagcctgtggcttaaccacagaattgccttcgatactgtttggcttgagaccggaag
aggtaagtggaactgcgagtgtagaggtgaaattcgtagatattcgcaagaacaccagtggcgaaggcggcttactggtccggttctgacgctg
aggcgcgaaagcgtggggagcaaaca

>fe8125f8bd994e1de94dc689dcd87b93

tgaggaatattgggcaatggaggcaactctgacccagccatgccgcgtgcaggaagacggccctatgggttgtaaactgcttttatacaggaag
aaacactgctacgtgtagcagcttgacggtactgtaagaataagcaccggctaactccgtgccagcagccgcggtaatacgggggatgcaagc

gttatccggaatgattgggcgtaaagcgtctgtaggtggctttttaagtccgccgtcaaatcccagggctcaactctggacaggcggtggaaact
accaagctggagtacggtaggggcagagggaatttccggtggagcggtgaaatgcgtagagatcggaaagaacaccaacggcgaaagcact
ctgctgggccgacactgacactgagagacgaaagctaggggagcgaatg

>fea58baacaed7fed052727e0dc6fca86

tagggaatcttccgcaatggacgaaagtctgacggagcaacgccgcgtgtatgaagaaggttttcggatcgtaaagtactgttgttagagaaga
acaaggataagagtaactgcttgtcccttgacggtatctaaccagaaagccacggctaactccgtgccagcagccgcggtaatacggagggtgc
gagcgttaatcggaattactgggcgtaaagcgcacgcaggcggtttgttaagctagatgtgaaagccccgagctcaacttgggatggtcatttag
aactggcagactagagtcttggagaggggagtggaattccaggtgtagcggtgaaatgcgtagatatctggaggaacatcagtggcgaaggcg
actccctggccaaagactgacgctcatgtgcgaaagtgtgggtagcgaaca

>ff581c00dc3966b25d46a2e627dc18df

tgaggaattttccgcaatgggcgaaagcctgacggagcaataccgcgtgagggatgaaggattttggtctgtaaacctcttttctcaagaaagaa
gttctgacggtacttgaggaataagcatcggctaactccgtgccagcagccgcggtaatacgggggatgcaagcgttatccggatttattgggcg
taaagcgcctgtaggttgtttaataagtctgttgttaaagactagggcttaaccctaggaaagcaatggaaactactagacttgagtatgatagg
ggtagagggaatttctagtgtagcggtgaaatgcgtagatattagaaagaacaccggtggcgaaggcgctctactggatcattactgacactca
gaggcgaaagctagggtagcaaaag

>ffa5266b3d35614dbc0f2f0c9ea2924f

tggggaatattgcacaatgggcgcaagcctgatgcagccatgccgcgtgtatgaagaaggccttcgggttgtaaagtactttcagtcgtgagga
aggcatttgcgttaatagcgtaagtgtttgacgttagcgacagaagaagcaccggctaactccgtgccagcagccgcggtaatacggagggtgc
gagcgttaatcggaattactgggcgtaaagcgcatgcaggtggttcgttaagtcagatgtgaaagcccggggctcaacctcggaattgcatttga
aactggcggactagagtgctgtagagggggtagaatttcaggtgtagcggtgaaatgcgtagagatctgaaggaataccggtggcgaaggcg
gcccccctggacagacactgacactcagatgcgaaagcgtggggagcaaaca
