# Supplementary material for: Assessing the Effects of Rotifer Feed Enrichments on Turbot (Scophthalmus maximus) Larvae and Post-Larvae Gut-Associated Bacterial Communities
Source: Microorganisms. 2023 Feb 17;11(2):520. doi: 10.3390/microorganisms11020520 (PMC9962078; doi:10.3390/microorganisms11020520)
Supplement: Supplementary file 1 [file microorganisms-11-00520-s001.zip › Supplementary File S2.pdf]

Assessing the Effects of Rotifer Feed Enrichments on Turbot (Scophthalmus maximus) Larvae and Post-Larvae Gut-Associated Bacterial Communities

Antonio Louvado[1], Carolina Castro[2], Davide A. M. Silva[1], Vanessa Oliveira[1], Luís E. C. Conceição[3], Daniel F. R. Cleary[1] and Newton C. M. Gomes[1]*

[1]Department of Biology and Centre for Environmental and Marine Studies (CESAM), University of Aveiro, 3810-193 Aveiro, Portugal

[2]Flatlantic—Actividades Piscícolas, SA 3070-732 Praia de Mira, Portugal

[3]SPAROS Lda., 8700-221 Olhao Portugal

Email: gomesncm@ua.pt

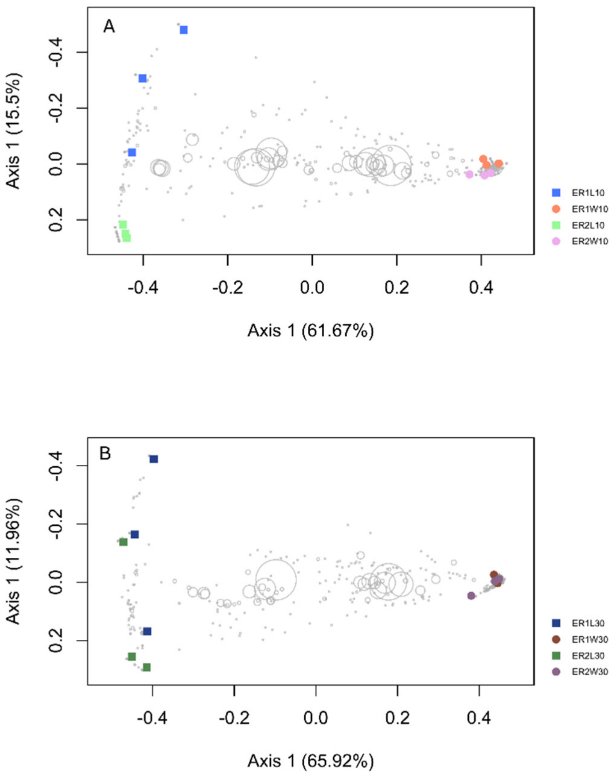

Supplementary Figure S1- First two axis of PCO of samples of the host and water associated bacterial communities at 10 DAH (A) and 30 DAH (B) . Colored symbols are samples. Grey symbols are weighted averages scores for ASV (size is proportional to abundance). ER1 and ER2 corresponds to the different rotifers diets. ER1L10 and ER2L10 (whole larvae at 10 DAH); ER1L30 and ER2L30 (post-larvae gut at 30 DAH); ER1W10 and ER2W10 (rearing water at 10DAH); ER1W30 and ER2W30 (rearing water at 30 DAH).

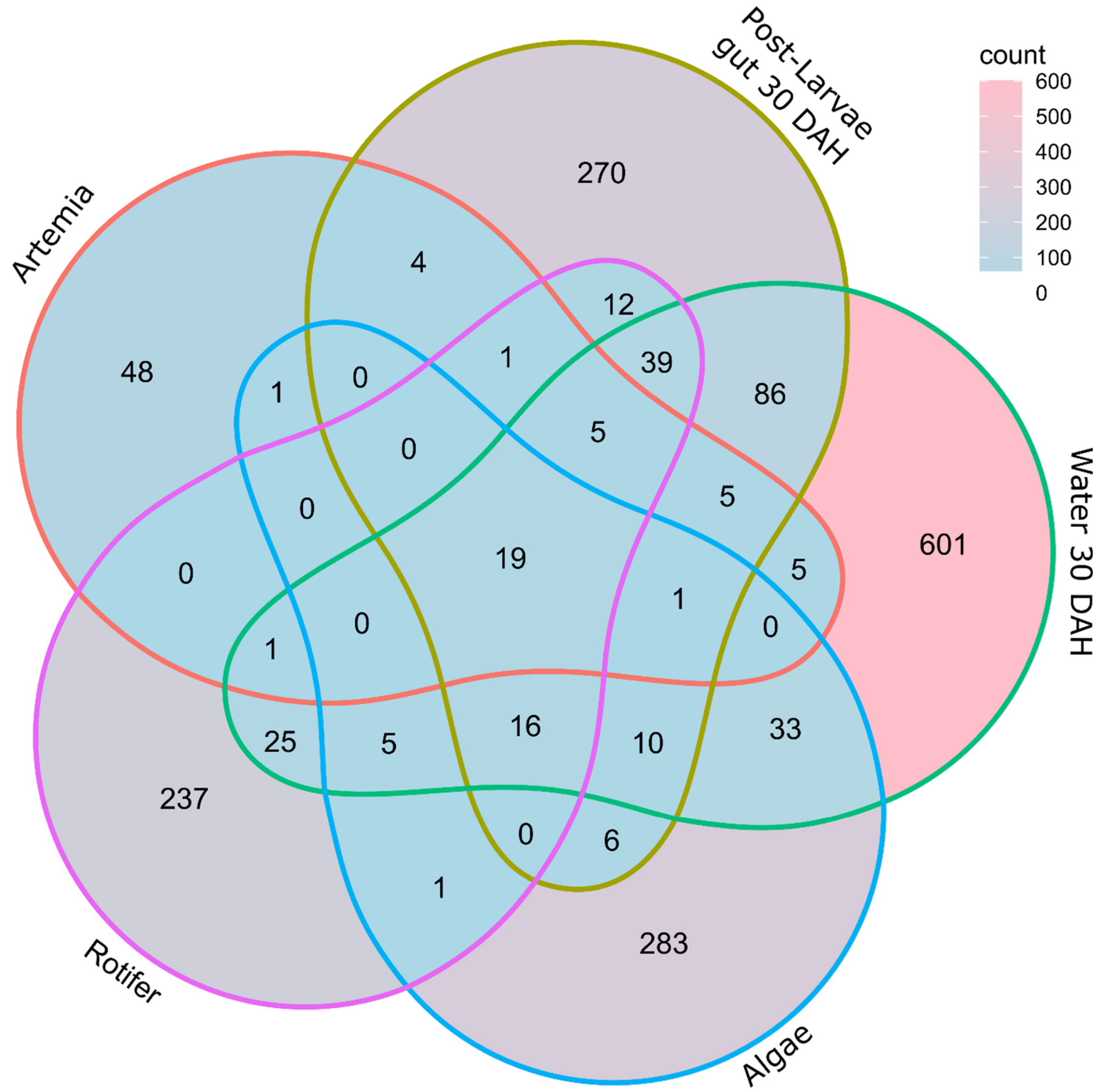

Supplementary Figure S2 - Venn diagram of whole larvae at 10DAH (Larvae), post-larvae gut at 30 DAH (Post-larvae gut), feed samples (Artemia, Rotifer and Algae) and water samples (Water) at 10 and 30 DAH, irrespective of diet.

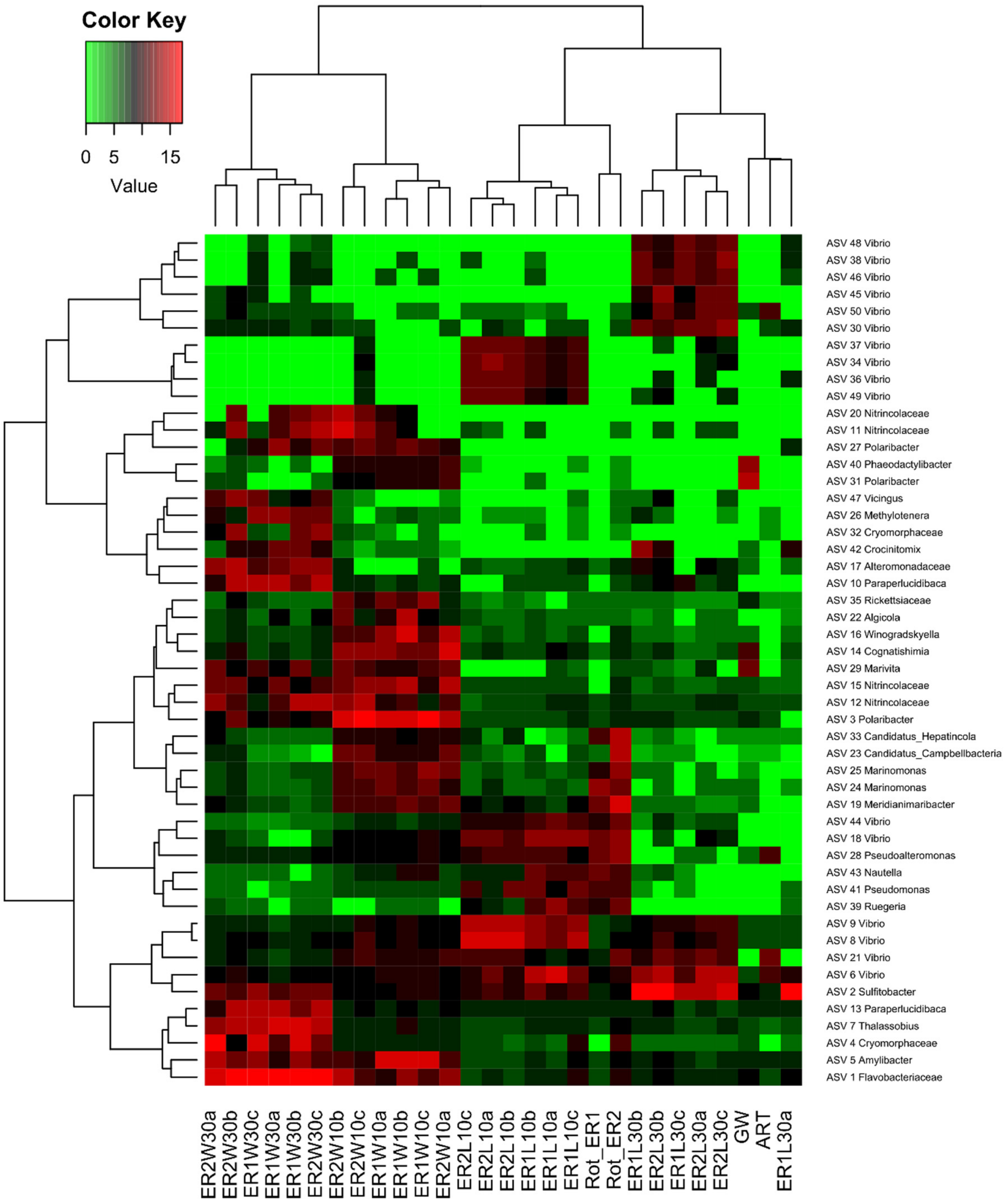

Supplementary Figure S3 - Heatmap analysis of the 50 most abundant ASVs. ASVs are represented by respective numbering (Supplementary Table S2) and the most conclusive taxonomic affiliation. ER1 and ER2 corresponds to the different rotifers diets. ART (Artemia at 30 DAH); GW (Algae at 10 DAH); Rot (Rotifers); ER1L10 and ER2L10 (whole larvae at 10 DAH); ER1L30 and ER2L30 (post-larvae gut at 30 DAH); ER1W10 and ER2W10 (rearing water at 10DAH); ER1W30 and ER2W30 (rearing water at 30 DAH). Small letters indicate tank-replicates.

Supplementary Table S1 - Detailed description of the 50 most abundant ASVs. Sim.: Sequence similarity; Ref: Reference.

		Affiliation base SILVA based classifier			Sequence Similarity on NCBI RefSeq database using BLAST (Altschul et al., 1990)				
ASV	ASV Nº	Family	Genus	Species	Closest relatives	Acc. Nº	Sim.(%)	Source	Ref
f068d1b23ce6707fc7b98827e7c03943	1	Flavobacteriaceae	Unclassified	Unclassified	*Tenacibaculum aestuariivivum* JDTF-79<br>*Tenacibaculum ovolyticum* NBRC 15947<br>*Polaribacter dokdonensis* DSW-5<br>*Tenacibaculum aiptasiae* a4	NR_159126<br>NR_113826<br>NR_043456<br>NR_044202	97.16	Tidal flat;<br>Atlantic Halibut eggs (*Hippoglossus hippoglossus*)<br>Seawater;<br>Sea anemone<br>*Aiptasia pulchella*	(Hansen et al., 1992; Park et al., 2017; Wang et al., 2008; Yoon et al., 2006)
c115905e9fcddb82a523014b244586e9	2	Rhodobacteraceae	*Sulfitobacter*	Unclassified	*Sulfitobacter profundi* SAORIC-263<br>*Sulfitobacter marinus* SW-265<br>*Sulfitobacter indolifex* DSM 14862	NR_165714<br>NR_043936<br>NR_115898	97.76	Deep sea seawater;<br>Seawater;<br>Seawater	(Song et al., 2019; Wagner-Döbler et al., 2004; Yoon et al., 2007)
c1c435c1a7911f20fbb7c0c15bb3d407	3	Flavobacteriaceae	*Polaribacter*	Unclassified	*Polaribacter dokdonensis* DSW-5	NR_043456	98.82	Seawater	(Yoon et al., 2006)
9da32dfb58ea8ef0a1ccee05ecf0b4b7	4	Cryomorphaceae	uncultured	Unclassified	*Phaeocystidibacter marisrubri* G18	NR_136475	90.8	Marine Sediment	(Zheng et al., 2015)
e688e029724fdfdd9cd096df2c443740	5	Rhodobacteraceae	*Amylibacter*	Unclassified	*Amylibacter ulvae* KMM 6515	NR_146351	100	Green alga *Ulva fenestrata*	(Nedashkovskaya et al., 2016)
a2c61bfd131f18c4fd375410fc0049f4	6	Vibrionaceae	*Vibrio*	Unclassified	*Vibrio anguillarum* NBRC 13266	NR_113609	100	Ulcerous lesion in cod (*Gadus morhua*)	(Thompson et al., 2011)
25865e882d21e0aa6fffa300375fc3ac	7	Rhodobacteraceae	*Thalassobius*	*Thalassobius mediterraneus*	*Thalassobius mediterraneus* XSM19	NR_042377	99.25	Seawater	(Arahal et al., 2005)
4a5ef3b18f362cc8c3d5ea749ca7831e	8	Vibrionaceae	*Vibrio*	Unclassified	*Vibrio lentus* CIP 107166	NR_114982	100	Oyster (*Crassostrea gigas*)	(Macián et al., 2001)
6d66f24ce1ac56865b36295b131385f5	9	Vibrionaceae	*Vibrio*	Unclassified	*Vibrio cyclitrophicus* LMG 21359	NR_115806	100	Creosote-contaminated marine sediments	(Hedlund and Staley, 2001)
4a43c52c99691294c452ec83619d039e	10	Moraxellaceae	*Paraperlucidibaca*	*Paraperlucidibaca baekdonensis*	*Paraperlucidibaca baekdonensis* RL-2	NR_117543	99.53	Seawater	(Oh et al., 2011)
9de3ab2c6c64ff335830f8a250d1e710	11	Nitrincolaceae	uncultured	uncultured bacterium	*Amphritea ceti* RA1	NR_133961	92.76	Beluga whale (*Delphinapterus leucas*) faeces	(Kim et al., 2014)

56e48910168e7044dff111da2e51d2d4	12	Nitrincolaceae	uncultured	uncultured bacterium	*Amphritea ceti* RA1	NR_133961	92.99	Beluga whale (*Delphinapterus leucas*) faeces	(Kim et al., 2014)
6afe589b6e0e15c66643b36ede16c14b	13	Moraxellaceae	*Paraperlucidibaca*	*Paraperlucidibaca baekdonensis*	*Paraperlucidibaca baekdonensis* RL-2	NR_117543	100	Seawater	(Oh et al., 2011)
c0de2aca9d901a366c09c136da891332	14	Rhodobacteraceae	*Cognatishimia*	uncultured bacterium	*Celeribacter ethanolicus* NH195<br>*Celeribacter baekdonensis* L-6	NR_146678<br>NR_117908	98.26	Seawater	(Lee et al., 2012; Shu-Ling et al., 2016)
1bef2c8662671b4d49e4d6877cb116cc	15	Nitrincolaceae	uncultured	uncultured bacterium	*Marinobacterium boryeongense* DMHB-2	NR_171449	93.21	Seawater	(Kang et al., 2019)
b794c29434fdd47d21f1ea42c9f09e1e	16	Flavobacteriaceae	*Winogradskyella*	Unclassified	*Winogradskyella echinorum* KMM 6211	NR_044564	98.82	Sea urchins	(Nedashkovskaya et al., 2009)
6907fa7b6a720b79b874a2127027e858	17	Alteromonadaceae	Unclassified	Unclassified	*Moraxella oblonga* IAM 14971	NR_112188	87.18	Oral cavity of sheep	(Xie and Yokota, 2005)
c2c6067d787e7e87047ff0a20673bdcd	18	Vibrionaceae	*Vibrio*	*Vibrio diazotrophicus*	*Vibrio diazotrophicus* NS<br>*Vibrio plantisponsor* MSSRF60	NR_026123<br>NR_108600	100	Sea urchin gastrointestinal tract<br>Mangrove	(Rameshkumar et al., 2011; Ruimy et al., 1994)
7843e3c8f3cb59b0c63b1337733ad7a6	19	Flavobacteriaceae	*Meridianimaribacter*	Unclassified	*Gaetbulibacter jejuensis* CNURIC014<br>*Meridianimaribacter flavus* NH57N	NR_116705<br>NR_116617	99.76	Seawater<br>Marine sediment	(Oh et al., 2010; Wang et al., 2010)
9d06939aa0a80b5a3bd483f4ec563d9f	20	Nitrincolaceae	uncultured	uncultured bacterium	*Amphritea ceti* RA1	NR_133961	92.76	Beluga whale (*Delphinapterus leucas*) faeces	(Kim et al., 2014)
8b4e1dd8ce89f7406fe32819e1f336b6	21	Vibrionaceae	*Vibrio*	Unclassified	*Vibrio hyugaensis* 090810a<br>*Vibrio alginolyticus* ATCC 17749<br>*Vibrio natriegens* DSM 759<br>*Vibrio galatheae* S2757<br>*Vibrio harveyi* NBRC 15634	NR_145569<br>NR_044825<br>NR_117890<br>NR_147758<br>NR_113784	100	Seawater<br>Spoiled horse mackerel<br>Marine sponge *Scleritoderma cyanea*<br>Mussel<br>Luminescing amphipod (*Talorchestria* sp.)	(Hoffmann et al., 2012; Urbanczyk et al., 2015)<br>(Giubergia et al., 2016; Hoffmann et al., 2012; Urbanczyk et al., 2013)
7fc64535448fdd7bd6f0ffa1de3edfec	22	Pseudoalteromonadaceae	*Algicola*	*Algicola bacteriolytica*	*Algicola bacteriolytica* E8R	NR_036838	99.3	*Laminaria japonica* with red-spot disease	(Sawabe et al., 1998)
564c9a3544155ce4ac6b88052911bb23	23	*Ca.*Campbellbacteria	*Ca.*Campbellbacteria	uncultured bacterium	*Parasynechococcus marenigrum* WH 8102	NR_176791	75.12	Seawater	(Palenik et al., 2003)
f563a708667c398dd5983fe8e08f064f	24	Marinomonadaceae	*Marinomonas*	uncultured bacterium	*Maribrevibacterium harenarium* HB171799	NR_173646	97.89	Coastal sand	(Mo et al., 2021)

1802662da29b8dfbdd7ccdd08a1df57b	25	Marinomonadaceae	*Marinomonas*	*Marinomonas* sp.	*Marinomonas pontica* 46-16	NR_042965	99.06	Seawater	(Ivanova et al., 2005)
99d50e0cd2405dd20e8a463874db4716	26	Methylophilaceae	*Methylotenera*	uncultured beta	*Methylotenera oryzisoli* La3113	NR_175447	95.78	Rice rhizosphere and soil	(Lv et al., 2018)
01f97950da8ddff76d1e2fe4b006b6c9	27	Flavobacteriaceae	*Polaribacter*	Unclassified	*Polaribacter* dokdonensis DSW-5	NR_043456	99.05	Seawater	(Yoon et al., 2006)
3dfb3c6cbb1c5b7e6290532430050d0e	28	Pseudoalteromonadaceae	*Pseudoalteromonas*	Unclassified	*Pseudoalteromonas gelatinilytica* NH153<br>*Pseudoalteromonas arabiensis* k53<br>*Pseudoalteromonas mariniglutinosa* KMM 3635	NR_152003<br>NR_113220<br>NR_028992	100	Seawater<br>Marine sediment<br>diatom *Chaetoceros lauderi*	(Matsuyama et al., 2013; Romanenko et al., 2003; Yan et al., 2016)
ddd6a46dc8af7661b360f3f9c33da991	29	Rhodobacteraceae	*Marivita*	Unclassified	*Marivita roseacus* CB1052<br>*Marivita cryptomonadis* CL-SK44<br>*Marivita litorea* CL-JM1	NR_132662<br>NR_044514<br>NR_044513	100	Estuary<br>Phytoplankton *Cryptomonas* sp.<br>Coastal seawater	(Budinoff et al., 2011; Hwang et al., 2009)
425de3fa772e20f85bc482817841792e	30	Vibrionaceae	*Vibrio*	Unclassified	*Vibrio gigantis* LGP 13	NR_044079	99.77	Oysters (*Crassostrea gigas*) haemolymph	(Le Roux et al., 2005)
3dffc1372f8aca1f92d0fc968cd797cf	31	Flavobacteriaceae	*Polaribacter*	Unclassified	*Polaribacter dokdonensis* DSW-5	NR_043456	99.53	Seawater	(Yoon et al., 2006)
624953349a47e0a2972534803c3dbb1b	32	Cryomorphaceae	uncultured	uncultured *Owenweeksia*	*Vicingus serpentipes* ANORD5	NR_159281	90.76	Epilithic biofilm marine	(Wiese et al., 2018)
074446dfe202a77ff094742b2f978f73	33	*Ca.*Hepatincola	*Ca.*Hepatincola	*Cherax quadricarinatus*	*Pseudaminobacter granuli* Gr-2	NR_165691	83.33	Wastewater treatment plant	(Hahn et al., 2017)
a34cdfc67699ad0b7b9141389684639a	34	Vibrionaceae	*Vibrio*	Unclassified	*Vibrio lentus* CIP 107166<br>*Vibrio splendidus* LMG 4042	NR_114982<br>NR_042156	99.77	Oyster (*Crassostrea gigas*)	(Le Roux et al., 2004; Macián et al., 2001)
b634bed192f76a84fdddf0bfc9b72f8d	35	Rickettsiaceae	uncultured	metagenome	*Orientia* tsutsugamushi Karp<br>*Orientia chuto* str. Dubai	NR_025860<br>NR_117903	90.07	Human infected	(Derrick and Brown, 1949; Izzard et al., 2010)
1f2946719ed27a9967dfbe65deb7f3eb	36	Vibrionaceae	*Vibrio*	Unclassified	*Vibrio cyclitrophicus* LMG 21359	NR_115806	99.77	Creosote-contaminated marine sediments	(Hedlund and Staley, 2001)
5d81207da0d0a7234a8b1ab7bc7e15b4	37	Vibrionaceae	*Vibrio*	Unclassified	*Vibrio lentus* CIP 107166	NR_114982	99.77	Oyster (*Crassostrea gigas*)	(Macián et al., 2001)

2751464a7b61ed160db5fa53bf800056	38	Vibrionaceae	*Vibrio*	Unclassified	*Vibrio toranzoniae* Vb 10.8	NR_117680	99.53	Clams (Venerupis philippinarum and V. decussata)	(Lasa et al., 2013)
386c723a1301c09364f5283533e33ba4	39	Rhodobacteraceae	*Ruegeria*	Unclassified	*Ruegeria arenilitoris* G-M8	NR_109635	99.75	Coastal sand near seaweed farm	(Park and Yoon, 2012)
9cc02ddcf035ba6736436e7418bc84de	40	Saprospiraceae	*Phaeodactylibacter*	*Phaeodactylibacter xiamenensis*	*Phaeodactylibacter xiamenensis* KD52	NR_134132	99.76	Marine algae *Phaeodactylum* tricornutum	(Chen et al., 2014)
52d9269c1a06765708b14aa1343cf02d	41	Pseudomonadaceae	*Pseudomonas*	*Pseudomonas oleovorans*	*Pseudomonas khazarica* TBZ2	NR_169334	99.77	Sediments Caspian Sea	(Tarhriz et al., 2020)
5735128e79ecd7e544dbc404550f1e27	42	Crocinitomicaceae	*Crocinitomix*	uncultured Crocinitomix	*Crocinitomix algicola* 0182	NR_158097	94.08	Marine algae *Gracilaria blodgettii*	(Shi et al., 2017)
762c63c2523466e5c18168ed06a73211	43	Rhodobacteraceae	*Nautella*	Unclassified	*Phaeobacter italicus* LMG 24365	NR_042673	100	Biofilm on cathode immersed in seawater	(Vandecandelaere et al., 2009)
a50c49d26c83479691d4d1e9d9055d46	44	Vibrionaceae	*Vibrio*	Unclassified	*Vibrio parahaemolyticus* ATCC 17802	NR_117893	99.3	Shirasu food poisoned victim	(Fujino et al., 1974; Yang et al., 2015)
4f7891e3f49e6a1603df14f964fe4889	45	Vibrionaceae	*Vibrio*	*Vibrio fortis*	*Vibrio fortis* CAIM 629	NR_025575	100	Shrimp larvae (*Litopenaeus vannamei*)	(Thompson et al., 2003a)
1a9301a03c70f53dc126bbf6b1aada9e	46	Vibrionaceae	*Vibrio*	Unclassified	*Vibrio gigantis* LGP 13	NR_044079	99.77	Oysters (*Crassostrea gigas*) haemolymph	(Le Roux et al., 2005)
43a5e3686c50b190009288f80a42646a	47	Cryomorphaceae	*Vicingus*	uncultured bacterium	*Acidiluteibacter ferrifornacis* S-15	NR_174308	94.34	Mangrove near port environment	(Gui et al., 2020)
e126d0d3db2858c647557595da6b24e9	48	Vibrionaceae	*Vibrio*	Unclassified	*Vibrio gigantis* LGP 13	NR_044079	99.53	Oysters (*Crassostrea gigas*) haemolymph	(Le Roux et al., 2005)
946f1b3bf75d231e55cd9294801a6c41	49	Vibrionaceae	*Vibrio*	Unclassified	*Vibrio kanaloae* LMG 20539	NR_042468	100	Diseased oyster larvae (*Ostrea edulis*)	(Thompson et al., 2003b)
8ac510326bc676a42e83bb237a1386ab	50	Vibrionaceae	*Vibrio*	Unclassified	*Vibrio parahaemolyticus* ATCC 17802	NR_117893	99.3	Shirasu food poisoned victim	(Fujino et al., 1974; Yang et al., 2015)
